# Predictors of survival among cervical cancer patients in Swaziland (Eswatini): A population-based analysis in a resource-limited setting (2016-2024)

**DOI:** 10.1371/journal.pgph.0006309

**Published:** 2026-04-16

**Authors:** Sithembiso S. Msibi, Citlalli Lopez, Fezokuhle N. Khumalo, L. Joseph Su

**Affiliations:** 1 O’Donnell School of Public Health, University of Texas Southwestern Medical Center, Dallas, Texas, United States of America; 2 Center for Global Health Practice and Impact, Georgetown University, Mbabane, Swaziland; PLOS: Public Library of Science, UNITED STATES OF AMERICA

## Abstract

Eswatini (formerly Swaziland) has one of the highest prevalence rates of HIV at 25.4% and only recently implemented an HPV vaccine in 2023. The country also reported the highest incidence and mortality rates of cervical cancer in 2021, estimated at 84.5 and 55.7 per 100,000 female population, respectively. This study serves as the first survival analysis of cervical cancer patients in Eswatini, aimed at examining the impact of clinical and demographic factors on cervical cancer survival outcomes. The Eswatini Cancer Registry was screened for cancer patients from 2016 to 2024, yielding a total of 2,349 cervical cancer patients, of whom 603 deaths were analyzed using the Cox proportional hazards model. The primary analysis assessed the association between cancer stage at diagnosis and survival, adjusting for potential confounders (age, smoking status, HIV status, treatment, and basis of diagnosis). Most patients (44.27%) were aged 50 years or older at diagnosis, whereas 31.42% were between 40 and 49 years and 24.31% were 39 years or younger. A significantly higher proportion of patients did not receive any treatment (59.05%, p < 0.001). Advanced cancer stages (Stages 3 and 4) were associated with significantly reduced survival times, with Stage 4 patients experiencing a median survival of about 10 months compared to about 40 months for Stage 1. Treatments (chemotherapy, radiation, surgery) reduced the hazard of death by 30% (HR: 0.70, 95% CI: 0.59-0.84, p < 0.001). In this first population-based survival analysis of cervical cancer in Eswatini, advanced stage at diagnosis and lack of treatment emerged as the strongest predictors of mortality, consistent with evidence in survival of cancer patients. These findings highlight the urgent need to expand access to screening, ensure early detection, and strengthen treatment capacity.

## Introduction

Cervical cancer continues to be a significant public health challenge globally [1]. Between 2018 and 2030, the global annual number of new cervical cancer cases is projected to rise from 570,000–700,000, with annual deaths expected to increase from 311,000–400,000 [2]. The Sub-Saharan African region bears a particularly heavy burden, with the highest reported incidence and mortality rates [3,4]. The age-standardized incidence rate of cervical cancer in sub-Saharan Africa in 2020 was 131.1 per 100,000 women, and this is the most common cause of cancer death among women in 21 of the 48 sub-Saharan African countries [1]. Despite being recognized as highly preventable and, if detected early, highly treatable, cervical cancer continues to claim the lives of thousands of women each year, with low- and middle-income countries experiencing the highest rates of incidence and mortality. In Eswatini (formerly Swaziland), the impact of cervical cancer is especially severe, exacerbated by the country’s high human immunodeficiency virus (HIV) prevalence rate of 25.4% [5] – one of the highest in the world. Cervical cancer is the leading cause of death amongst women in Eswatini, with an estimated incidence rate of 84.5 per 100,000 women and a death rate of 55.7 per 100,000 women [6]. The economic burden of the disease is equally significant. The total annual cost of cervical cancer treatment in Eswatini is estimated at $19 million from a societal perspective, with a majority of these costs attributable to late diagnosis and productivity loss due to premature mortality. When juxtaposed against the country’s annual GDP of approximately $5 billion for a population of 1.2 million [7], these figures make evident the substantial economic burden that cervical cancer places on Eswatini.

HIV infection, a known risk factor for persistent human papillomavirus (HPV) infection and the progression of cervical lesions to cancer, further intensifies the cervical cancer crisis in the country [8]. Although Eswatini has made significant progress in controlling the HIV epidemic, achieving the UNAIDS 95-95-95 goals ahead of the 2030 target, some gaps still remain. Women are disproportionately affected, with an incidence rate that is seven times higher than that of men (1.11% vs 0.17%) [5]. On the basis that women living with HIV are six times more likely to develop cervical cancer compared to women who are HIV negative [2], it is critical that this dual burden is eliminated. As the HIV epidemic in Eswatini continues to evolve, so do associated diseases like cervical cancer, emphasizing the critical need to focus on survival outcomes to improve long-term health and care strategies. In 2020, the World Health Organization (WHO) introduced the Global Cervical Cancer Elimination Initiative, aiming to expedite efforts to eradicate cervical cancer [2]. The initiative seeks to lower the incidence to fewer than 4 cases per 100,000 women annually in all countries, thereby addressing global disparities in cervical cancer outcomes. Central to this initiative is the 90–70–90 target, which includes vaccinating 90% of girls by the age of 15, ensuring 70% of women undergo high-performance screening at least twice by age 45, and providing treatment for 90% of women diagnosed with cervical precancer or cancer. In line with achieving the goal of cervical cancer elimination, Eswatini initiated its first-ever HPV vaccination campaign for young girls in June 2023 with coverage reaching approximately 59% amongst girls aged 9 – 14 years [9]. This is far from the 90% target signaling the need for more targeted efforts towards reaching the 2030 targets.

Persistently low cervical cancer screening rates, coupled with numerous barriers to achieving full vaccine coverage, contribute to the ongoing risk of cervical cancer-related morbidity [6,10,11]. Without comprehensive screening and prevention strategies, cervical cancer remains a looming threat to the health and well-being of women in Eswatini. While the global community has made strides in the prevention and early detection of cervical cancer through HPV vaccination and screening programs, these initiatives often face implementation challenges in resource-limited settings like Eswatini [6,12]. Barriers such as inadequate health infrastructure, limited human resources, stigma, and low awareness hinder the effective rollout and uptake of these life-saving interventions [11]. Moreover, disparities in access to care mean that many women in Eswatini present with advanced stages of cervical cancer [13,14], where survival outcomes are poor, and treatment options are limited.

Strategic Objective 4 of the Eswatini National Cancer Prevention and Control Strategy (2019) focuses on enhancing cancer surveillance, advancing research, and improving strategic information systems [14]. To date, research on cervical cancer in Eswatini has primarily focused on prevalence, risk factors, and screening uptake. However, significant knowledge gaps remain in understanding the survival patterns of cervical cancer patients, particularly in relation to key factors such as HIV status, stage at diagnosis, and access to treatment. Survival analysis is a powerful epidemiological tool that can provide critical insights into the time-to-event outcomes, such as death or disease recurrence, and the associated predictors [15,16]. Despite its potential to inform policy and programmatic decisions, no published study has applied survival analysis to cervical cancer patients in Eswatini, highlighting an urgent need for locally relevant data to drive improvements in patient care and health system performance.

This study seeks to fill this critical gap by conducting the first survival analysis of cervical cancer patients in Eswatini. Analyzing survival outcomes and their determinants will contribute to the growing body of knowledge on cervical cancer in the region and provide actionable insights to improve health outcomes for women. The findings will support national efforts to strengthen cervical cancer prevention and management, aligning with the global targets set forth by the WHO's Global Strategy to Accelerate the Elimination of Cervical Cancer. Ultimately, this study aims to serve as a foundational resource for future research and interventions to address cervical cancer in Eswatini.

## Materials and methods

### Ethics statement

This study was conducted in accordance with ethical standards of the Declaration of Helsinki. The authors analyzed de-identified data provided by the Eswatini National Cancer Registry (ENCR) in June 2025, in compliance with relevant ethical considerations and guidelines. The ethics approval committee was the Eswatini Health and Human Research Review Board (FWA00026661/IRB00011253). As the study involved a retrospective review of de-identified data, informed consent from individual patients was not required.

**Fig 1:**
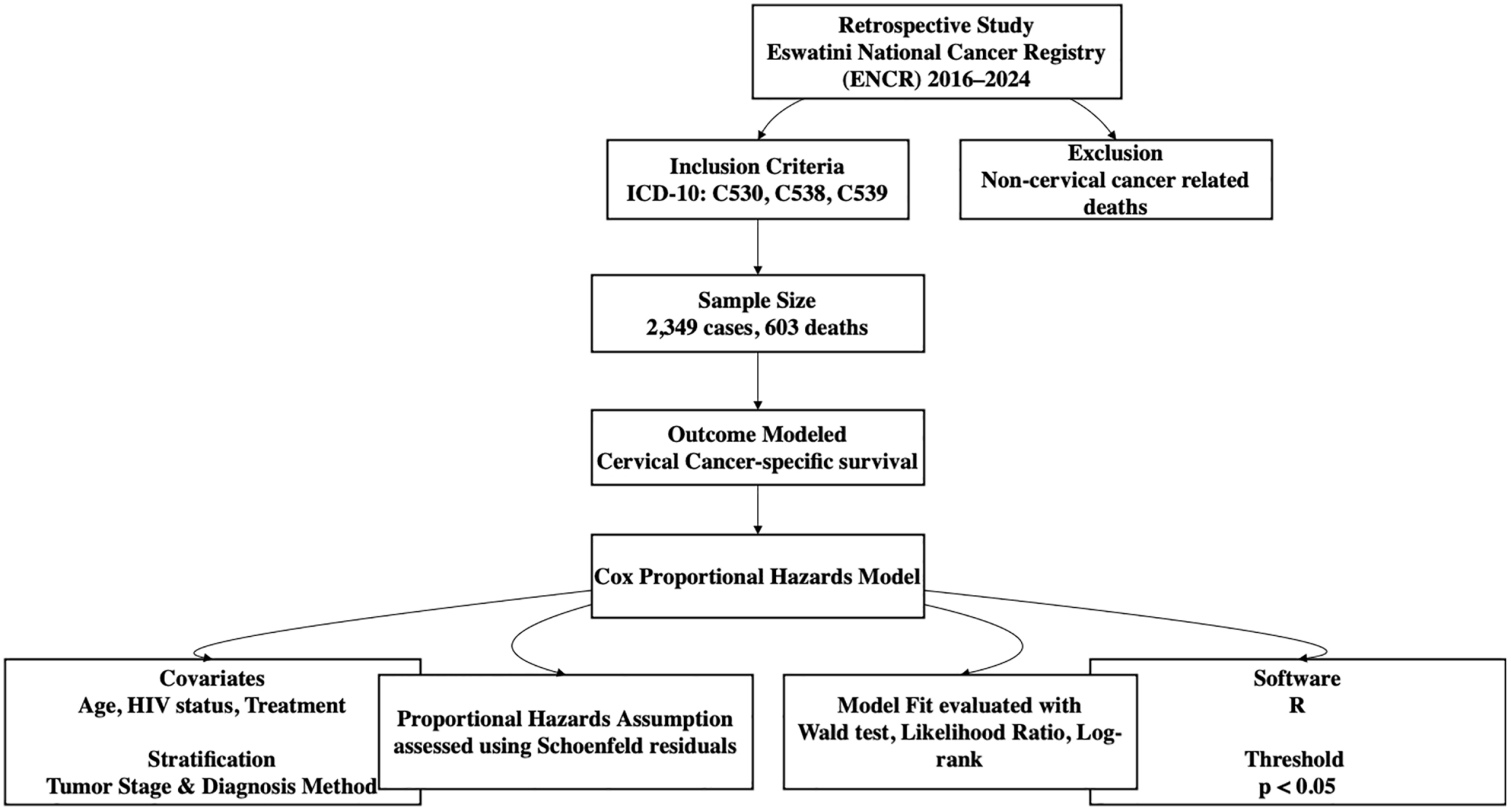
Flow chart showing the data analysis process.

In this study, we included patients diagnosed with cervical cancer from the Eswatini National Cancer Registry (ENCR) from the inception of the registry in 2016–2024. ENCR is a population-based cancer registry that collects data from various sources such as clinical records, pathology reports and death registries [17]. The data we received from ENCR included the demographic and pathologic characteristics of patients and tumors. The study included patients with confirmed diagnosis of cervical cancer, classified according to the International Classification of Diseases for Oncology (ICD-O) codes. All patients with ICD-10 codes C539, C538, and C530 were included in our analysis. The primary outcome was cause-specific survival, defined as the time from diagnosis to death from cervical cancer. Patients who had cervical cancer but had a different cause of death were not included in our analysis. Covariates included demographic variables, tumor characteristics (stage, histology, behavior), treatment modalities (surgery, radiation, chemotherapy), smoking status, alcohol consumption, and HIV status. We began with an initial cohort of 2,570 cervical cancer patients extracted from the registry. However, upon reviewing the dataset, we identified cases with missing critical data, such as the cause of death and time-to-event variables, which are essential for survival analysis. To ensure data completeness, we excluded patients with incomplete variables of interest. After this refinement process, the final study population was reduced to 2,349 patients (Fig 1).

### Statistical analysis

Descriptive statistics were employed to summarize patients’ demographic statistics. The patients were categorized into three age groups (≤39, 40–49, and ≥50 years). The data were censored using the binary outcome of survival status (alive vs. cervical cancer-related death) and Kaplan-Meier survival curves were generated to estimate survival probabilities, with comparisons across groups assessed using the log-rank test. For Cox proportional models, we used the proportional hazards test and further implemented the 10% rule for confounding in determining which covariates to include in the multivariable models. Cox proportional hazards regression models were used to identify factors associated with survival outcomes, with results expressed as hazard ratios (HRs) and 95% confidence intervals (CIs). To assess the impact of missing HIV status on our model estimates, we conducted sensitivity analyses using different assumptions in imputing the missing data. Given that 46% of the cohort had missing HIV status, a sensitivity analysis was conducted to assess the robustness of the findings under varying assumptions. Five alternative models were tested: (1) imputing missing HIV status based on observed cohort prevalence, (2) assuming all unknowns were HIV-negative, (3) assuming all were HIV-positive, (4) omitting HIV status entirely, and (5) the original model with an “unknown” category. For each scenario, we compared the estimated HRs, CIs, and model concordance using Harrell’s C-index with the main model. The proportional hazards assumption was assessed using Schoenfeld residuals. Due to a high proportion of missing data, smoking status, alcohol use, and family history of cancer were excluded from the final Cox proportional hazard model. Statistical analyses were performed using R [18].

## Results

A total of 2,349 cervical cancer patients were included in this analysis. The majority of patients (44.27%) were aged 50 years or older, while 31.42% were between the ages of 40 and 49, and 24.31% were 39 years or younger at the time of diagnosis (Table 1).

**Table 1:** Patients’ demographic and clinical characteristics.

Variable	Category	N = 2349 (%)	Kaplan-Meier Log-Rank p-value
**Age Group**	≤39	571 (24.31%)	0.061
	40-49	738 (31.42%)	
	≥50	1040 (44.27%)	
**Smoking Status**	No	319 (13.58%)	<0.001
	Yes	31 (1.32%)	
	Unknown	1999 (85.10%)	
**Alcohol Use**	No	297 (12.64%)	<0.001
	Yes	50 (2.13%)	
	Unknown	2002 (85.23%)	
**HIV Status**	Negative	253 (10.77%)	0.12
	Positive	1014 (43.17%)	
	Unknown	1082 (46.06%)	
**Family History**	No	253 (10.77%)	<0.001
	Yes	61 (2.60%)	
	Unknown	2035 (86.63%)	
**Cancer Stage**	Stage 1	383 (16.30%)	<0.001
	Stage 2	527 (22.44%)	
	Stage 3	448 (19.07%)	
	Stage 4	269 (11.45%)	
	Stage not determined	722 (30.74%)	
**Treatment Received**	No	1387 (59.05%)	<0.001
	Yes	962 (40.95%)	

The distribution of cancer stages at diagnosis showed patterns of late presentation, with 19.07% diagnosed at Stage 3 and 11.45% at Stage 4 (Table 1). Notably, 30.74% of patients lacked documented stage information. Only 16.3% of cases were diagnosed at Stage 1. With respect to treatment, 40.95% of patients received some form of cancer-directed therapy, which included surgery, chemotherapy, and/or radiation, while 59.05% did not receive treatment

**Fig 2:**
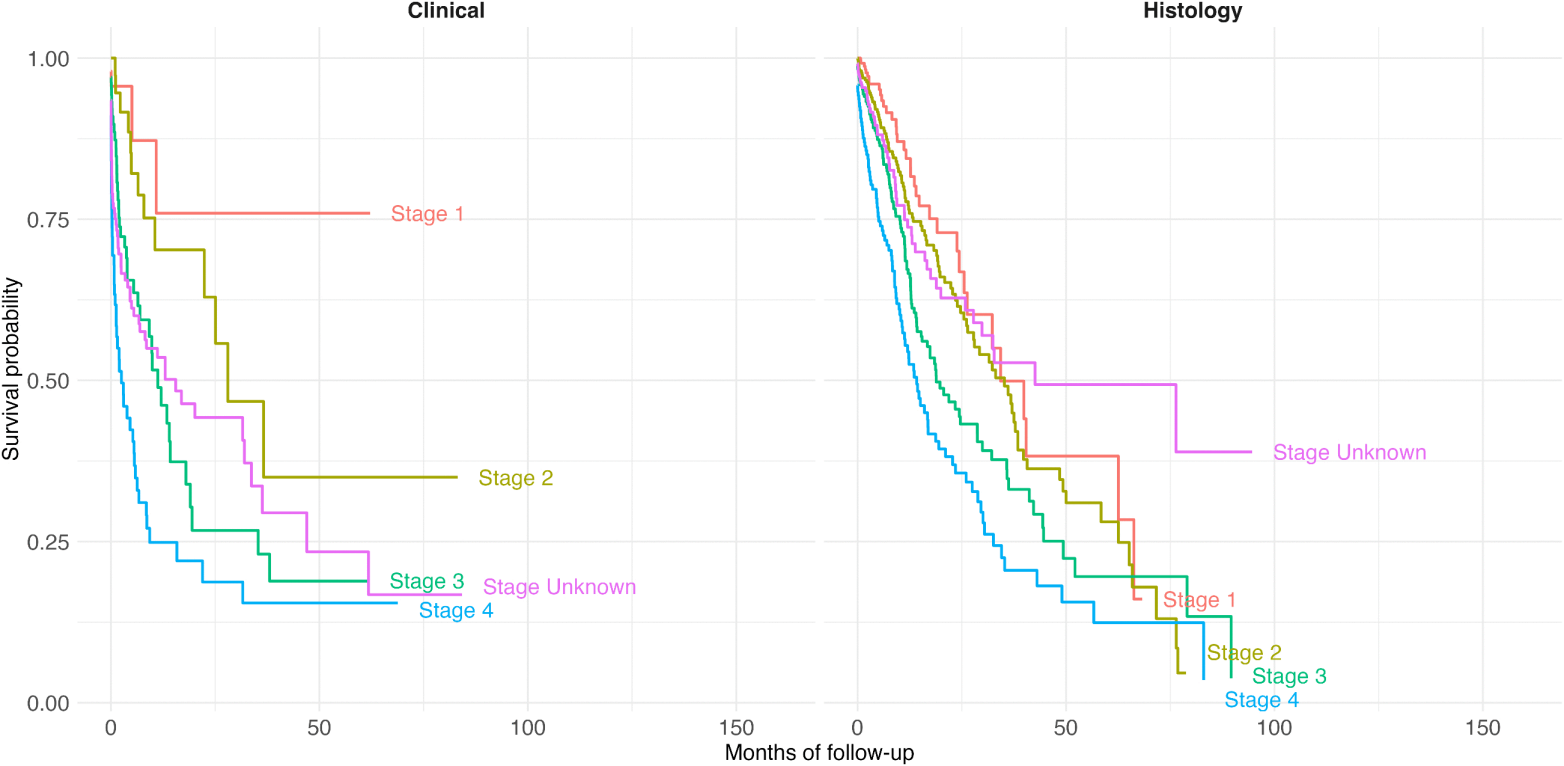
Survival curve of cervical cancer patients, stratified by cancer stage at diagnosis and basis of diagnosis.

The Kaplan-Meier survival analysis revealed significant differences in survival across cancer stages (log-rank p < 0.001), with more advanced stages associated with reduced survival probabilities (Fig 2). Median survival time for patients diagnosed at Stage 1 was approximately 40 months, compared to only 10 months for patients diagnosed at Stage 4 (S1 Table). Survival curves also indicate meaningful variation by treatment status, with treatment significantly associated with prolonged survival. Compared to patients diagnosed at Stage 1, those diagnosed at Stage 3 had a 2.57-fold increased risk of mortality, and those diagnosed at Stage 4 had a 4.12-fold increased risk.

Cox proportional hazards regression analysis identified several factors significantly associated with survival outcomes. Receiving any form of treatment reduced the hazard of death by 30% (HR = 0.70, 95% CI: 0.59–0.84, p < 0.001) (Table 2). Cancer stage at diagnosis was a strong predictor of survival, with advanced stages associated with substantially higher hazards of death. Smoking and alcohol consumption were also associated with significantly poorer survival outcomes (p < 0.001), though the proportion of patients with documented smoking and alcohol use was small, with the majority of records listing these behaviors as unknown.

**Table 2:** Multivariable Cox Proportional Hazards Model Stratified by stage at diagnosis and basis of diagnosis.

Variable	HR	95% CI	p-value
**Age Group** (Ref: ≤ 39)			
40–49	0.96	0.76 – 1.20	0.71
≥50	1.13	0.91 – 1.40	0.27
**HIV Status** (Ref: Negative)			
Positive	1.20	0.93 – 1.55	0.17
Unknown	1.27	0.97 – 1.66	0.083
**Treatment (Yes vs. No)**	0.70	0.59 – 0.84	<0.001

The analysis also assessed the role of HIV status in influencing survival outcomes. While no statistically significant interaction was observed between HIV status and either treatment or cancer stage, there was a marginally significant survival benefit for HIV-positive patients diagnosed at Stage 1 (HR = 0.38, 95% CI: 0.13–1.07, p = 0.068).

Across all models, the estimated hazard ratios for treatment and age groups remained consistent, with treatment continuing to confer a significant survival benefit (HR range: 0.68–0.70, all p < 0.001). The HR for HIV-positive status varied modestly between 1.02 and 1.23 across models and remained statistically non-significant in all scenarios (p-values: 0.10–0.58). The model concordance statistics ranged narrowly from 0.5638 to 0.5703, indicating minimal changes in the model's predictive performance [19]. This is shown in Table 3.

**Table 3:** Sensitivity analyses evaluating the effect of different assumptions regarding missing HIV status.

Model	Variable	HR	95% CI	p-value	Concordance
**Main Model**	Age: 40–49	0.96	(0.76, 1.20)	0.71	
	Age: ≥ 50	1.13	(0.90, 1.40)	0.27	
	HIV: Positive	1.20	(0.93, 1.55)	0.17	
	HIV: Unknown	1.27	(0.97, 1.66)	0.083	0.57
	TRT: Yes	0.70	(0.59, 0.84)	< 0.001	
**Prevalence - Imputed**	Age: 40–49	0.95	(0.76, 1.19)	0.66	
	Age: ≥ 50	1.11	(0.90, 1.38)	0.33	
	HIV: Positive	1.06	(0.86, 1.29)	0.58	
	TRT: Yes	0.69	(0.58, 0.82)	< 0.001	0.57
**All Unknown = Negative**	Age: 40–49	0.95	(0.76, 1.19)	0.64	
	Age: ≥ 50	1.10	(0.89, 1.37)	0.37	
	HIV: Positive	1.02	(0.86, 1.21)	0.84	
	TRT: Yes	0.69	(0.57, 0.82)	< 0.001	0.56
**All Unknown = Positive**	Age: 40–49	0.96	(0.76, 1.20)	0.70	
	Age: ≥ 50	1.14	(0.92, 1.41)	0.23	
	HIV: Positive	1.23	(0.96, 1.57)	0.10	
	TRT: Yes	0.70	(0.58, 0.83)	< 0.001	0.57
**Omit HIVSTAT**	Age: 40–49	0.98	(0.76, 1.19)	0.64	
	Age: ≥ 50	1.10	(0.89, 1.35)	0.38	
	TRT: Yes	0.69	(0.58, 0.82)	< 0.001	0.56

## Discussion

This study offers the first national survival analysis of cervical cancer patients in Eswatini, identifying late-stage diagnosis and lack of treatment as the primary drivers of mortality. Patients diagnosed at early stages had significantly better survival outcomes, while treatment was strongly associated with improved prognosis. The findings reinforce global and regional patterns [20,21] and underscore the urgent need to strengthen early detection, pathology services and access to treatment within Eswatini’s health system.

Cancer stage at diagnosis emerged as a dominant predictor of survival, with markedly worse outcomes observed among patients diagnosed at advanced stages. The association between the cervical cancer stage at diagnosis and survival is well-documented, with previous studies indicating that patients diagnosed at a later stage have lower treatment success rate and survival [15,22]. This pattern is also consistent with survival studies from sub-Saharan Africa and other LMICs, where delayed presentation remains a common barrier to favourable outcomes [4,16]. In Eswatini, this is further compounded by persistently low screening uptake and barriers to diagnostic services [10,11]. The survival gradient across stages observed in this study underscores the urgent need to scale up early detection efforts, particularly for women outside of routine HIV care, who may be less likely to undergo screening. Strengthening community-based outreach initiatives, integrating HPV testing into primary care and improving access to diagnostic technologies, will be critical for expanding screening coverage at population level and facilitating earlier stage detection.

Treatment received was the only significant predictor of survival, with treated patients experiencing a 30% lower hazard of cervical cancer-specific mortality (HR:0.70, 95% CI: 0.59-0.84), p < 0.001) compared to untreated patients. Alarmingly, nearly 60% of patients had no documented treatment, reflecting severe limitations in oncology service capacity in Eswatini. Similar challenges have been reported in Malawi, Uganda, and Lesotho [3],and may include infrastructure deficits, cost barriers, centralization of services, sociocultural barriers, and delays in referral pathways [23–26]. Addressing these gaps will require targeted investments in human resources, decentralization of oncology services, and enhanced patient navigation systems. Furthermore, the low prevalence of treatment may be due the cases presenting at late stage where only some form of palliative care can be offered.

The sensitivity analysis conducted suggests that the primary findings are robust to assumptions about missing HIV data and that HIV status, although epidemiologically important, did not significantly influence survival in this analysis. This aligns with findings from a prospective cohort study in Côte d'Ivoire, which reported no significant difference in overall survival between HIV-positive and HIV-negative women with invasive cervical cancer, despite higher treatment initiation rates among women living with HIV [27]. Another study at the Uganda Cancer Institute found that while HIV-positive women tended to present at a younger age and had shorter median overall survival than their HIV-negative counterparts (13.7 vs 24.3 months), HIV status was weakly associated with mortality after adjusting for age and cancer stage (HR: 1.3; 95% CI: 0.8–2.2) [28]. In contrast, several studies from the region have demonstrated that HIV status can significantly impact cervical cancer outcomes [29,30]. For example, research from Nigeria found that HIV-positive women were diagnosed at a younger age and had lower overall survival probabilities than HIV-negative counterparts. Similarly, a retrospective cohort study in Botswana found that HIV infection was associated with an almost twofold increased hazard of death among cervical cancer patients, even after adjusting for other covariates [31]. These differences may be attributed to variability in health system contexts, ART coverage, and the degree of service integration. In Eswatini, the attenuated impact of HIV on cervical cancer survival may be explained by the strength of the national HIV response, which has achieved the UNAIDS 95-95-95 targets and integrated cancer screening into HIV care platforms [5].

This integration may facilitate earlier detection and improved follow-up among women living with HIV, as demonstrated by findings from other settings where integrated services have led to improved uptake of cervical cancer screening among women living with HIV [32–34]. However, challenges remain, particularly in ensuring that women who test positive for cervical lesions receive timely and effective treatment, a gap that has been identified in both Eswatini and across the region [35–37].

Together, these findings reinforce that late-stage diagnosis and lack of treatment are the strongest predictors of mortality in cervical cancer patients in Eswatini. While HIV status was not a statistically significant determinant of survival in this context, the high proportion of missing data and the modest differences in hazard ratios across sensitivity scenarios underscore the need for more granular studies. Future research should explore the nuanced interactions between HIV status, ART adherence, and access to oncology services to fully understand the role of HIV in cervical cancer survival across diverse settings in sub-Saharan Africa.

Our study has its limitations. The analysis was constrained by data completeness challenges, particularly in lifestyle risk factors (e.g., smoking, alcohol), family history, HIV status, staging documentation, and other variables that may affect the precision of survival estimates. Over 30% of patients lacked cancer stage information, highlighting systemic gaps in clinical documentation. Strengthening registry systems through digitized data capture and improved integration with clinical records will be essential to improve data quality.

## Conclusion

This study confirms that advanced cervical cancer stage and lack of treatment are the strongest predictors of mortality. Early diagnosis through screening and ensuring treatment accessibility are the most effective strategies for improving survival. While age and HIV status were not significantly associated with survival, targeted interventions for older and HIV-positive patients should be explored to ensure equitable access to care. Addressing data limitations and improving the assessment of lifestyle and genetic risk factors will be crucial for refining future survival models. Further, it highlights the central role of tumor stage and diagnostic methods in determining cervical cancer survival outcomes. Patients diagnosed at earlier stages show significantly better survival, and histological confirmation of stage may offer enhanced prognostic accuracy over clinical staging. Importantly, receiving treatment was strongly associated with improved survival, while age and HIV status were not significant predictors in the adjusted model.

Our findings directly support the goals of Eswatini’s National Cancer Prevention and Control Strategy, particularly its objectives to improve surveillance, treatment access, early detection and improve the consistency and accuracy of clinical data collection. They also align with the WHO's global initiative to eliminate cervical cancer as a public health problem, reinforcing the importance of expanding HPV vaccination, increasing screening coverage, and addressing treatment bottlenecks.

## Supporting information

S1 Table Summary of survival differences by cancer stage and treatment status. (DOCX)

S1 Text All raw statistical outputs, model results and diagnostic checks generated during analyses. (PDF)

S1 Data Raw de-identified dataset used for all analyses. (XLSX)

## Abbreviations

HIV: Human Immunodeficiency Virus
HPV: Human papillomavirus
ICD-O: International Classification of Diseases for Oncology
WHO: World Health Organization
ENCR: Eswatini National Cancer Registry
