# Supplementary material for: Predictors of survival among cervical cancer patients in Swaziland (Eswatini): A population-based analysis in a resource-limited setting (2016-2024)
Source: PLOS Glob Public Health. 2026 Apr 16;6(4):e0006309. doi: 10.1371/journal.pgph.0006309 (PMC13086319; doi:10.1371/journal.pgph.0006309)
Supplement: S1 Table — (DOCX) [file pgph.0006309.s001.docx]

**S1 Table.** Summary of Survival Differences by Cancer Stage and Treatment Status

| **Survival Metric** | **Finding** | **Interpretation** |
| --- | --- | --- |
| Overall survival differences by cancer stage | Log-rank p < 0.001 | Significant variation in survival across stages (Figure 2). |
| Median survival time – Stage 1 | ≈ 40 months | KM curve shows survival probability crossing 0.5 at ~40 months. |
| Median survival time – Stage 4 | ≈ 10 months | KM curve drops below 0.5 at ~10–12 months. |
| Effect of treatment on survival | Treatment associated with prolonged survival | Supported by KM curves and Cox models. |
| HR for treatment (Yes vs No) | 0.70 | 95% CI: 0.59–0.84, p < 0.001. |
| HR for Stage 3 vs Stage 1 | 2.57 | Patients diagnosed at Stage 3 have ~2.6× higher mortality risk. |
| HR for Stage 4 vs Stage 1 | 4.12 | Patients diagnosed at Stage 4 have ~4.1× higher mortality risk. |
