# Supplementary material for: Predictors of survival among cervical cancer patients in Swaziland (Eswatini): A population-based analysis in a resource-limited setting (2016-2024)
Source: PLOS Glob Public Health. 2026 Apr 16;6(4):e0006309. doi: 10.1371/journal.pgph.0006309 (PMC13086319; doi:10.1371/journal.pgph.0006309)
Supplement: S1 Text — (PDF) [file pgph.0006309.s002.pdf]

Cervical Cancer Survival Analysis

2025-12-02

DESCRIPTIVE STATISTICS

```
# Define categorical variables
categorical_vars <- c("Age_Group", "SMOKE", "ALCOHOL", "HIVSTAT", "FAMHIST","CAUSEOFDEATH", "STAT", "ST

# Loop through categorical variables
for (var in categorical_vars) {
  cat("\nVariable:", var, "\n")
  freq <- table(cervical[[var]])  # Frequency
  pct <- prop.table(freq) * 100  # Percentage
  print(freq)
  print(round(pct, 2))
}
```

```
##
## Variable: Age_Group
##
##   <39   >50 40-49
##   571  1040   738
##
##   <39   >50 40-49
## 24.31 44.27 31.42
##
## Variable: SMOKE
##
##      No Unknown     Yes
##     319    1999      31
##
##      No Unknown     Yes
##   13.58   85.10    1.32
##
## Variable: ALCOHOL
##
##      No Unknown     Yes
##     297    2002      50
##
##      No Unknown     Yes
##   12.64   85.23    2.13
##
## Variable: HIVSTAT
```

```
##
## Negative Positive  Unknown
##      253     1014     1082
##
## Negative Positive  Unknown
##    10.77    43.17    46.06
##
## Variable: FAMHIST
##
##      No Unknown     Yes
##     253    2035      61
##
##      No Unknown     Yes
##   10.77   86.63    2.60
##
## Variable: CAUSEOFDEATH
##
## This cancer
##         605
##
## This cancer
##         100
##
## Variable: STAT
##
##   Alive    Dead Unknown
##    1750     589       4
##
##   Alive    Dead Unknown
##   74.69   25.14    0.17
##
## Variable: STAGE
##
##              Stage 1              Stage 2              Stage 3
##                  383                  527                  448
##              Stage 4 Stage not determined
##                  269                  722
##
##              Stage 1              Stage 2              Stage 3
##                16.30                22.44                19.07
##              Stage 4 Stage not determined
##                11.45                30.74
##
## Variable: BEH
##
## Malignant
##      2349
##
## Malignant
##       100
##
## Variable: TRT
##
##   No  Yes
```

```
## 1387  962
##
##    No   Yes
## 59.05 40.95
```

```r
# time2death for those deceased

# Filter for individuals who have died (Censor == 1)
deceased <- cervical[cervical$Censor == 1, ]

summary(deceased$time2death)
```

```
##    Min. 1st Qu.  Median    Mean 3rd Qu.    Max.
##   0.000   1.002   6.012  11.123  14.160  89.626
```

KAPLAN-MEIER & LOG-RANK TEST

KM - AGE

```r
# AGE
km_age <- survfit(Surv(time2death, Censor) ~ Age_Group, data = cervical)
summary(km_age)$table
```

```
##                  records n.max n.start events    rmean se(rmean)   median
## Age_Group=<39        571   571     571    134 34.42488  3.318206 23.85216
## Age_Group=>50       1040  1040    1040    286 32.60594  1.916341 23.49076
## Age_Group=40-49      738   738     738    183 38.00577  2.770749 25.49487
##                   0.95LCL  0.95UCL
## Age_Group=<39   17.97125 32.59138
## Age_Group=>50   19.18686 29.24025
## Age_Group=40-49 19.28542 35.84394
```

```r
dage <- data.frame(time = km_age$time,
                  n.risk = km_age$n.risk,
                  n.event = km_age$n.event,
                  n.censor = km_age$n.censor,
                  surv = km_age$surv,
                  upper = km_age$upper,
                  lower = km_age$lower
                  )
head(dage)
```

```
##         time n.risk n.event n.censor      surv     upper     lower
## 1 0.00000000    571       4       74 0.9929947 0.9998593 0.9861773
## 2 0.03285421    493       3       12 0.9869522 0.9966274 0.9773709
## 3 0.06570842    478       1       15 0.9848874 0.9953666 0.9745186
## 4 0.09856263    462       2        9 0.9806238 0.9926333 0.9687597
## 5 0.13141684    451       1        8 0.9784495 0.9911796 0.9658830
## 6 0.16427105    442       5        6 0.9673811 0.9833041 0.9517159
```

```r
ggsurvplot(km_age,
           pval = TRUE, conf.int = FALSE,
           risk.table = TRUE,
           risk.table.col = "strata",
           linetype = "strata",
           surv.median.line = "hv",
           ggtheme = theme_bw(),
          title = "Survival Curve by Age"
)
```

```
## Warning in geom_segment(aes(x = 0, y = max(y2), xend = max(x1), yend = max(y2)), : All aesthetics ha
## i Please consider using `annotate()` or provide this layer with data containing
##   a single row.
## All aesthetics have length 1, but the data has 3 rows.
## i Please consider using `annotate()` or provide this layer with data containing
##   a single row.
## All aesthetics have length 1, but the data has 3 rows.
## i Please consider using `annotate()` or provide this layer with data containing
##   a single row.
## All aesthetics have length 1, but the data has 3 rows.
## i Please consider using `annotate()` or provide this layer with data containing
##   a single row.
```

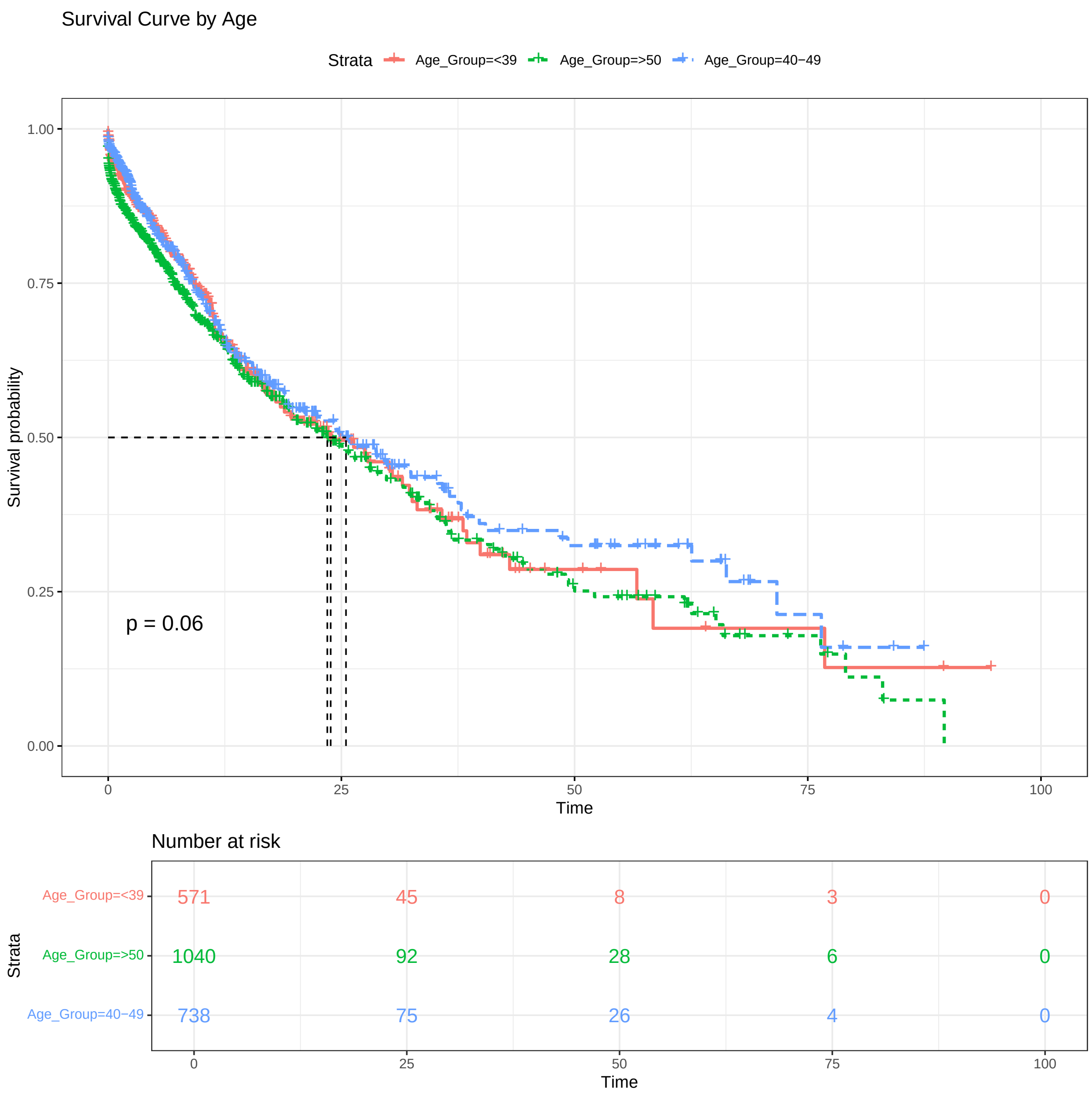


```r
ggsurvplot(km_age,
           conf.int = FALSE,
           risk.table.col = "strata",
           ggtheme = theme_bw(),
           fun = "cumhaz",
           title = "Cumulative Hazard Plot by Age"
)
```

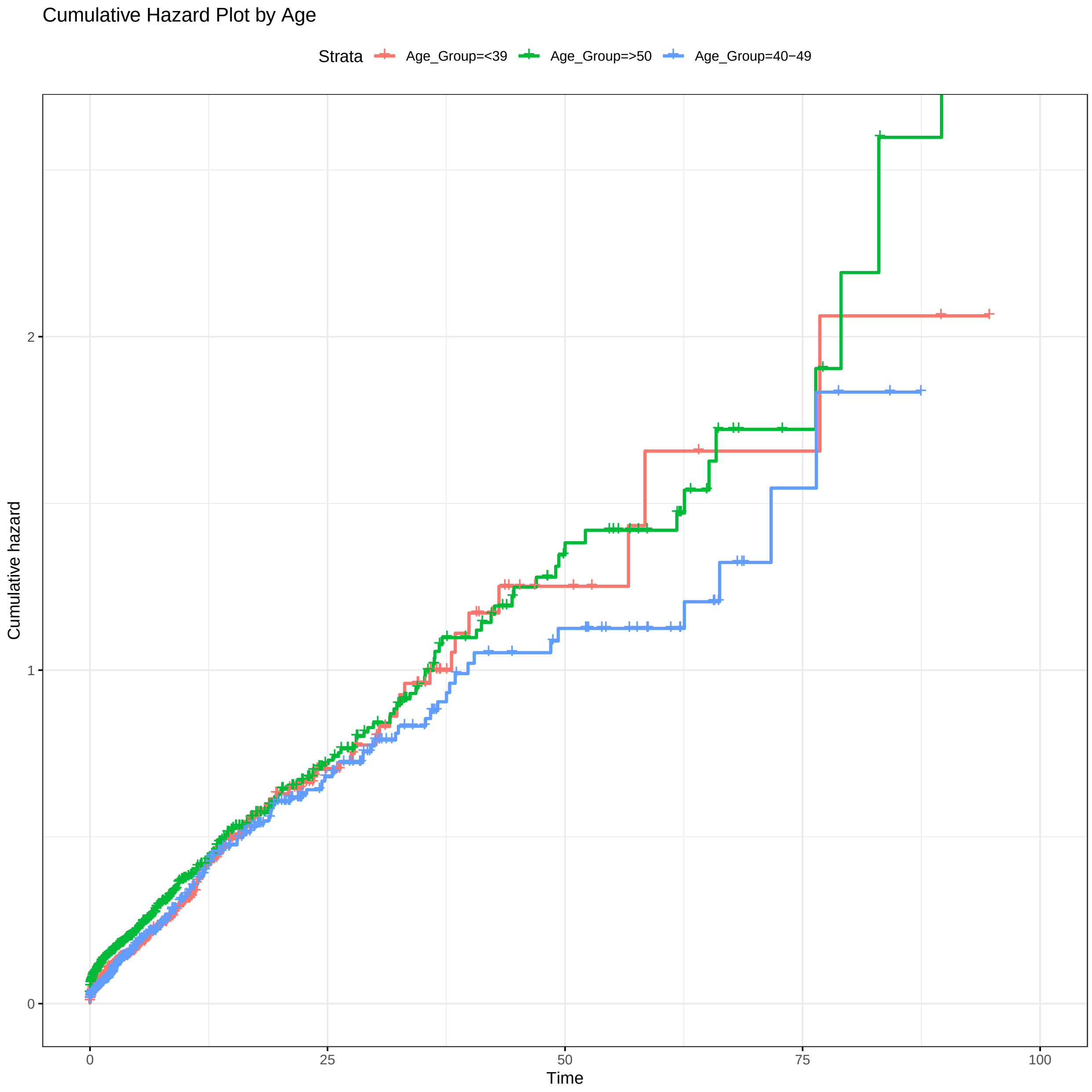


```r
# Log-rank test for Age_Group
agelrt <- survdiff(Surv(time2death, Censor) ~ Age_Group, data = cervical)

# View the test result
print(agelrt)
```

```
## Call:
## survdiff(formula = Surv(time2death, Censor) ~ Age_Group, data = cervical)
##
##                        N Observed Expected (O-E)^2/E (O-E)^2/V
## Age_Group=<39        571      134      141     0.364     0.478
## Age_Group=>50       1040      286      258     3.074     5.393
## Age_Group=40-49      738      183      204     2.160     3.280
```

```
##
##  Chisq= 5.6  on 2 degrees of freedom, p= 0.06
```

KM - STAGE

```
# STAGE
km_STAGE <- survfit(Surv(time2death, Censor) ~ STAGE, data = cervical)
summary(km_STAGE)$table
```

```
##                              records n.max n.start events    rmean se(rmean)
## STAGE=Stage 1                    383   383     383     40 45.92160  5.684885
## STAGE=Stage 2                    527   527     527    119 39.83901  2.699339
## STAGE=Stage 3                    448   448     448    158 30.56067  2.633723
## STAGE=Stage 4                    269   269     269    148 22.00534  2.390570
## STAGE=Stage not determined       722   722     722    138 39.39848  3.642619
##                                median   0.95LCL  0.95UCL
## STAGE=Stage 1              39.88501 32.295688       NA
## STAGE=Stage 2              36.10678 27.827515 39.78645
## STAGE=Stage 3              17.37988 13.995893 21.88090
## STAGE=Stage 4              10.15195  8.476386 14.39014
## STAGE=Stage not determined 27.79466 16.657084 36.30390
```

```
dSTAGE <- data.frame(time = km_STAGE$time,
                  n.risk = km_STAGE$n.risk,
                  n.event = km_STAGE$n.event,
                  n.censor = km_STAGE$n.censor,
                  surv = km_STAGE$surv,
                  upper = km_STAGE$upper,
                  lower = km_STAGE$lower
                  )
head(dSTAGE)
```

```
##         time n.risk n.event n.censor      surv upper     lower
## 1 0.00000000    383       1       23 0.9973890     1 0.9922914
## 2 0.03285421    359       1        3 0.9946108     1 0.9871860
## 3 0.06570842    355       0        7 0.9946108     1 0.9871860
## 4 0.09856263    348       0       11 0.9946108     1 0.9871860
## 5 0.13141684    337       0        9 0.9946108     1 0.9871860
## 6 0.16427105    328       0       15 0.9946108     1 0.9871860
```

```
ggsurvplot(km_STAGE,
           pval = TRUE, conf.int = FALSE,
           risk.table = TRUE,
           risk.table.col = "strata",
           linetype = "strata",
           surv.median.line = "hv",
           ggtheme = theme_bw(),
            title = "Survival Curve by Stage"
)
```

```
## Warning in geom_segment(aes(x = 0, y = max(y2), xend = max(x1), yend = max(y2)), : All aesthetics ha
## i Please consider using 'annotate()' or provide this layer with data containing
##   a single row.
## All aesthetics have length 1, but the data has 5 rows.
## i Please consider using 'annotate()' or provide this layer with data containing
##   a single row.
## All aesthetics have length 1, but the data has 5 rows.
## i Please consider using 'annotate()' or provide this layer with data containing
##   a single row.
## All aesthetics have length 1, but the data has 5 rows.
## i Please consider using 'annotate()' or provide this layer with data containing
##   a single row.
```

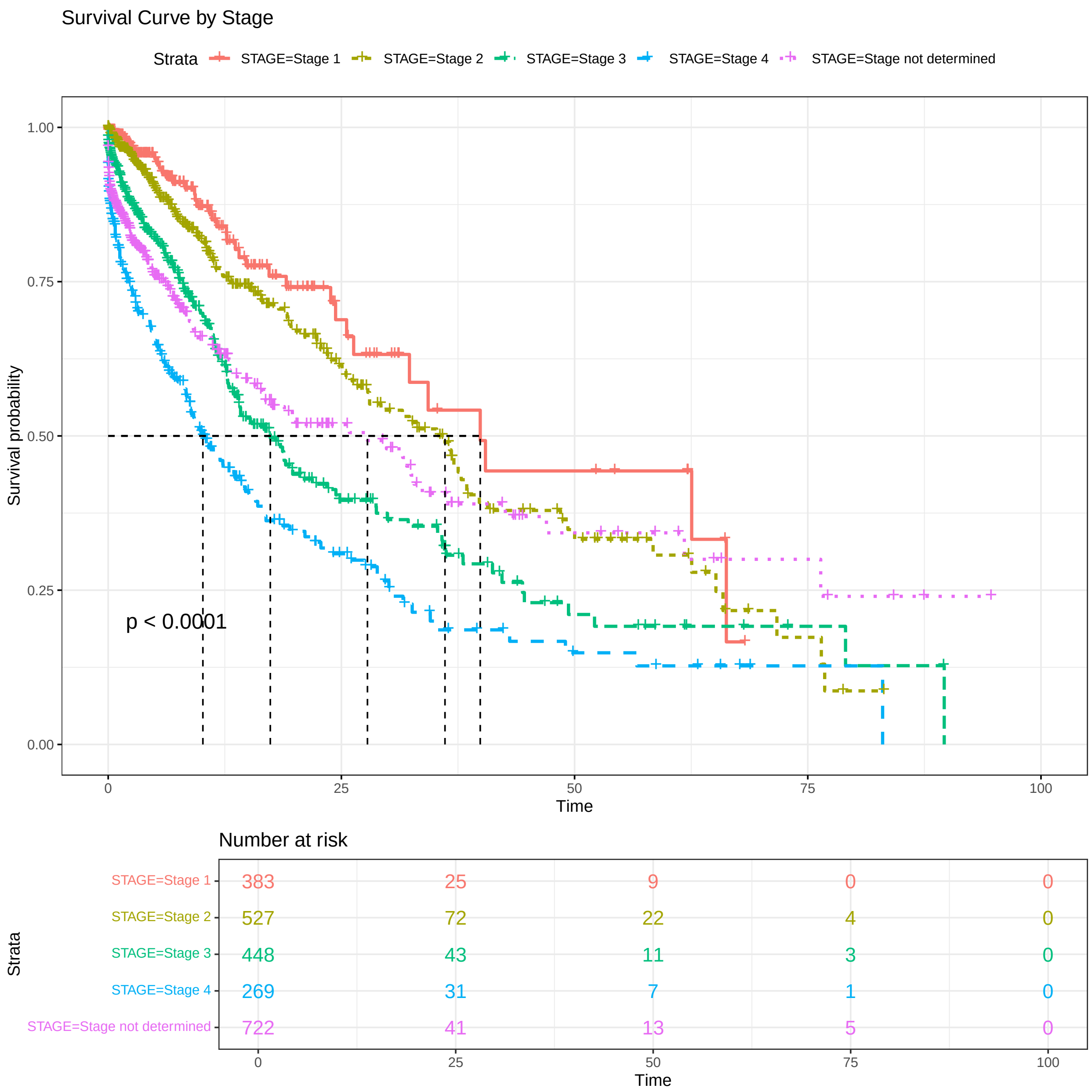

```r
ggsurvplot(km_STAGE,
           conf.int = FALSE,
           risk.table.col = "strata",
           ggtheme = theme_bw(),
           fun = "cumhaz",
           title = "Cumulative Hazard Plot by Stage"
)
```

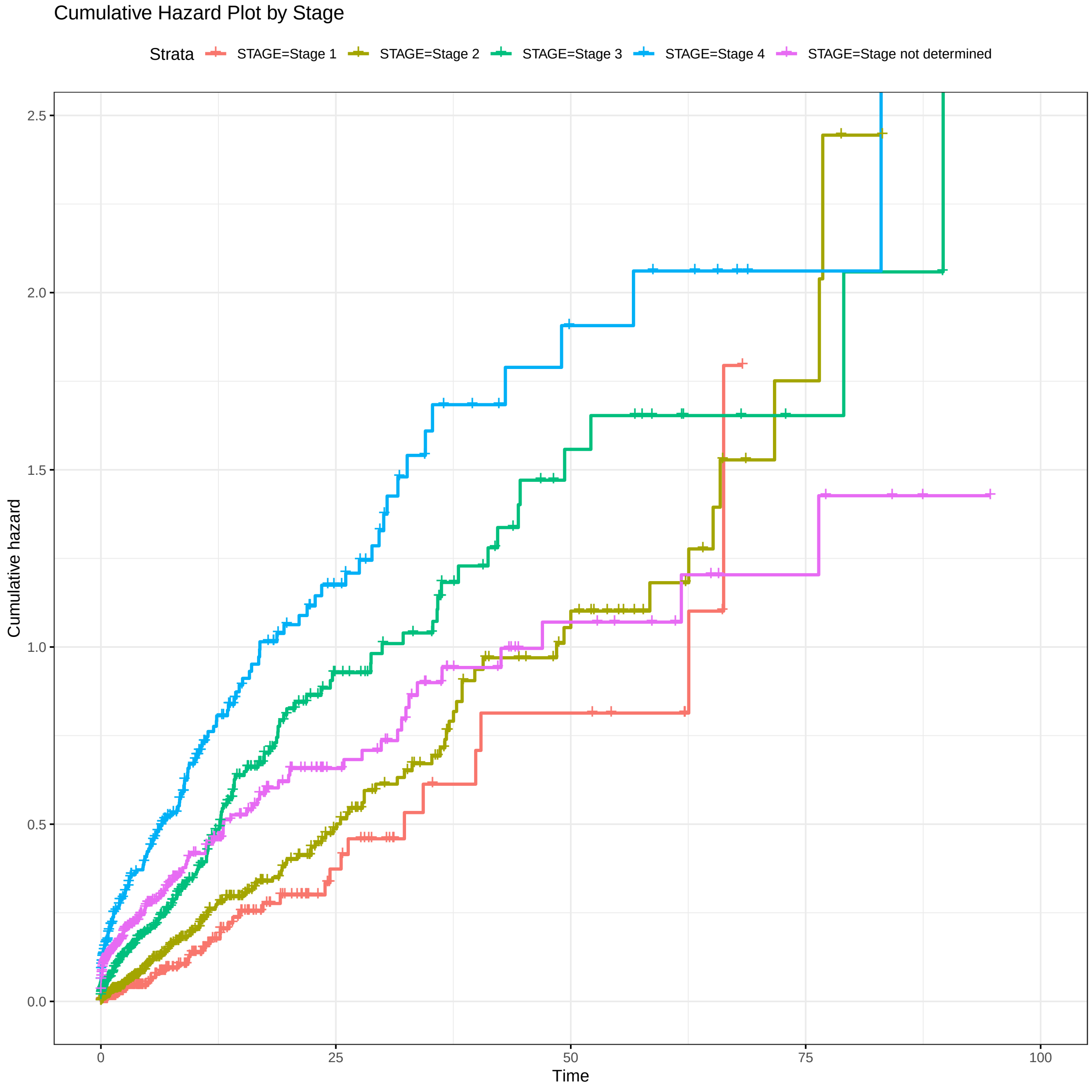


```r
# Log-rank test for STAGE
STAGElrt <- survdiff(Surv(time2death, Censor) ~ STAGE, data = cervical)

# View the test result
print(STAGElrt)
```

```
## Call:
## survdiff(formula = Surv(time2death, Censor) ~ STAGE, data = cervical)
##
##                                N Observed Expected (O-E)^2/E (O-E)^2/V
## STAGE=Stage 1                383       40     89.6     27.45     32.54
## STAGE=Stage 2                527      119    173.4     17.08     24.20
## STAGE=Stage 3                448      158    138.2      2.82      3.68
## STAGE=Stage 4                269      148     80.8     55.80     64.72
## STAGE=Stage not determined 722      138    120.9      2.42      3.07
##
##  Chisq= 106  on 4 degrees of freedom, p= <2e-16
```

KM - SMOKE

```r
# SMOKE
km_SMOKE <- survfit(Surv(time2death, Censor) ~ SMOKE, data = cervical)
summary(km_SMOKE)$table
```

```
##                records n.max n.start events    rmean se(rmean)   median  0.95LCL
## SMOKE=No           319   319     319     67 49.93037  4.101625 52.13963 28.02464
## SMOKE=Unknown     1999  1999    1999    531 30.93174  1.417180 21.88090 18.26694
## SMOKE=Yes           31    31      31      5 62.40891 11.180398 65.14990 65.14990
##                 0.95UCL
## SMOKE=No             NA
## SMOKE=Unknown 25.56057
## SMOKE=Yes            NA
```

```r
dSMOKE <- data.frame(time = km_SMOKE$time,
                  n.risk = km_SMOKE$n.risk,
                  n.event = km_SMOKE$n.event,
                  n.censor = km_SMOKE$n.censor,
                  surv = km_SMOKE$surv,
                  upper = km_SMOKE$upper,
                  lower = km_SMOKE$lower
                  )
head(dSMOKE)
```

```
##         time n.risk n.event n.censor     surv upper     lower
## 1 0.00000000    319       0        6 1.000000     1 1.0000000
## 2 0.03285421    313       0        1 1.000000     1 1.0000000
## 3 0.06570842    312       0        4 1.000000     1 1.0000000
## 4 0.09856263    308       0        1 1.000000     1 1.0000000
## 5 0.13141684    307       0        1 1.000000     1 1.0000000
## 6 0.16427105    306       1        3 0.996732     1 0.9903579
```

```r
ggsurvplot(km_SMOKE,
           pval = TRUE, conf.int = FALSE,
           risk.table = TRUE,
           risk.table.col = "strata",
           linetype = "strata",
           surv.median.line = "hv",
```

```r
           ggtheme = theme_bw(),
            title = "Survival Curve by Smoking Status"
)
```

```
## Warning in geom_segment(aes(x = 0, y = max(y2), xend = max(x1), yend = max(y2)), : All aesthetics hav
## i Please consider using ‘annotate()‘ or provide this layer with data containing
##   a single row.
## All aesthetics have length 1, but the data has 3 rows.
## i Please consider using ‘annotate()‘ or provide this layer with data containing
##   a single row.
## All aesthetics have length 1, but the data has 3 rows.
## i Please consider using ‘annotate()‘ or provide this layer with data containing
##   a single row.
## All aesthetics have length 1, but the data has 3 rows.
## i Please consider using ‘annotate()‘ or provide this layer with data containing
##   a single row.
```

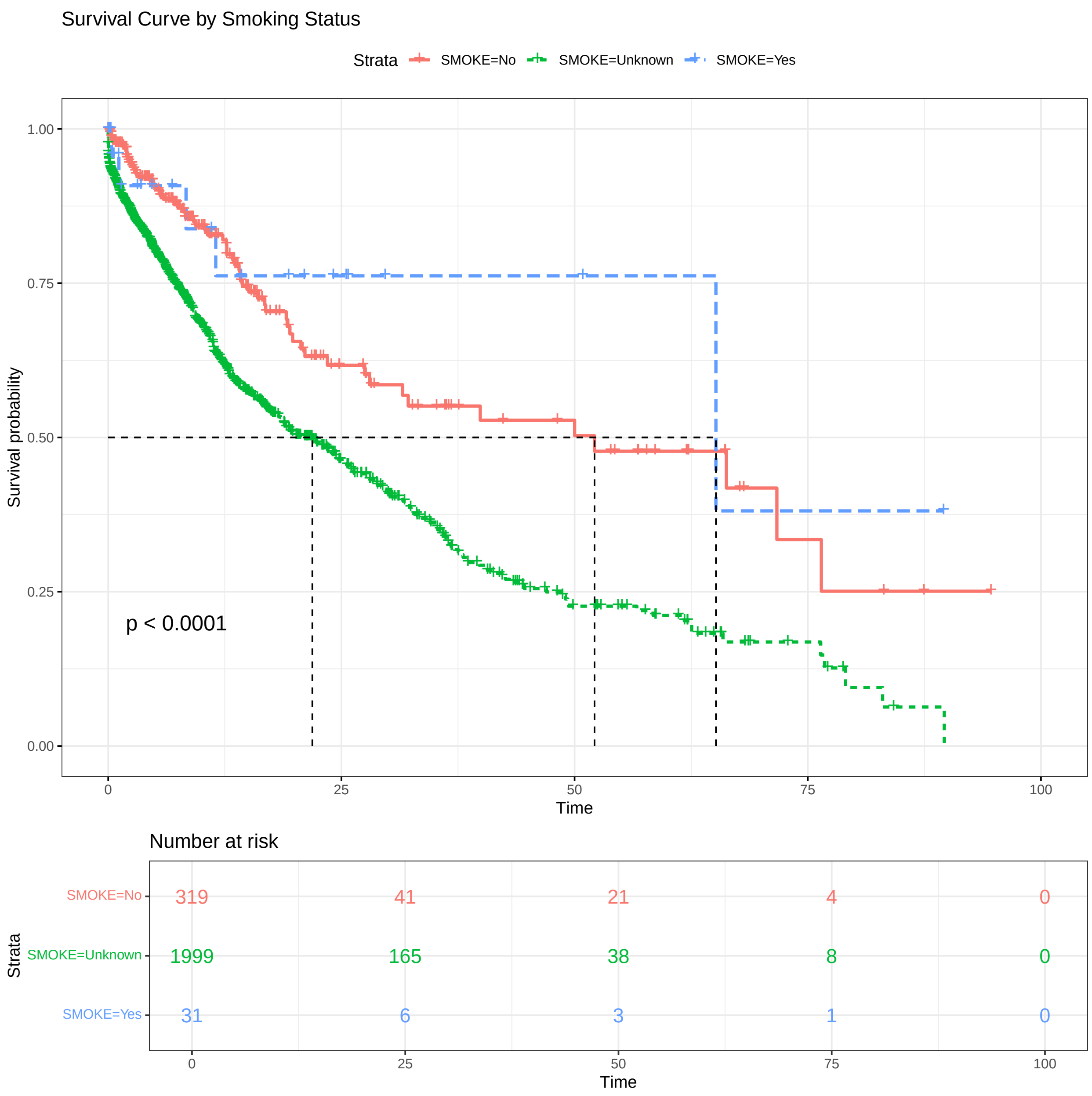


```r
ggsurvplot(km_SMOKE,
          conf.int = FALSE,
          risk.table.col = "strata",
          ggtheme = theme_bw(),
          fun = "cumhaz",
          title = "Cumulative Hazard Plot by Smoking Status"
)
```

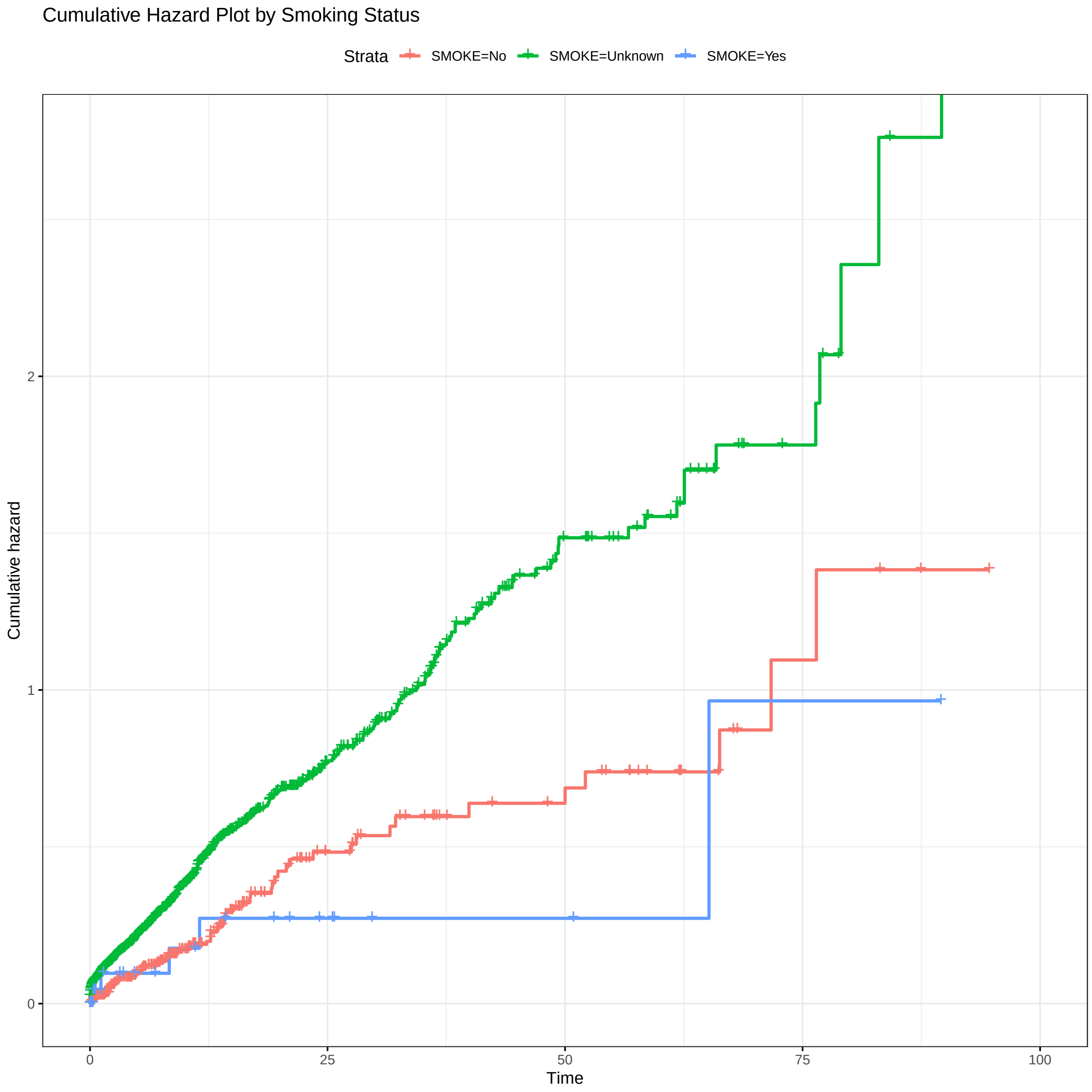


```r
# Log-rank test for SMOKE_Group
SMOKElrt <- survdiff(Surv(time2death, Censor) ~ SMOKE, data = cervical)

# View the test result
print(SMOKElrt)
```

```
## Call:
## survdiff(formula = Surv(time2death, Censor) ~ SMOKE, data = cervical)
##
##                  N Observed Expected (O-E)^2/E (O-E)^2/V
## SMOKE=No       319       67    119.9     23.33     29.47
## SMOKE=Unknown 1999      531    471.0      7.64     35.44
## SMOKE=Yes       31        5     12.1      4.16      4.29
```

```
##
##  Chisq= 35.7  on 2 degrees of freedom, p= 2e-08
```

KM - ALCOHOL

```
# ALCOHOL
km_ALCOHOL <- survfit(Surv(time2death, Censor) ~ ALCOHOL, data = cervical)
summary(km_ALCOHOL)$table
```

```
##                  records n.max n.start events    rmean se(rmean)  median
## ALCOHOL=No           297   297     297     63 50.37906  4.121480 65.1499
## ALCOHOL=Unknown     2002  2002    2002    534 30.75444  1.403886 21.8809
## ALCOHOL=Yes           50    50      50      6 67.19239 10.567481      NA
##                  0.95LCL  0.95UCL
## ALCOHOL=No      31.57290       NA
## ALCOHOL=Unknown 18.26694 25.56057
## ALCOHOL=Yes     27.49897       NA
```

```
dALCOHOL <- data.frame(time = km_ALCOHOL$time,
                  n.risk = km_ALCOHOL$n.risk,
                  n.event = km_ALCOHOL$n.event,
                  n.censor = km_ALCOHOL$n.censor,
                  surv = km_ALCOHOL$surv,
                  upper = km_ALCOHOL$upper,
                  lower = km_ALCOHOL$lower
                  )
head(dALCOHOL)
```

```
##         time n.risk n.event n.censor      surv upper     lower
## 1 0.00000000    297       0        5 1.0000000     1 1.0000000
## 2 0.03285421    292       0        1 1.0000000     1 1.0000000
## 3 0.06570842    291       0        4 1.0000000     1 1.0000000
## 4 0.09856263    287       0        2 1.0000000     1 1.0000000
## 5 0.13141684    285       0        1 1.0000000     1 1.0000000
## 6 0.16427105    284       1        4 0.9964789     1 0.9896135
```

```
ggsurvplot(km_ALCOHOL,
           pval = TRUE, conf.int = FALSE,
           risk.table = TRUE,
           risk.table.col = "strata",
           linetype = "strata",
           surv.median.line = "hv",
           ggtheme = theme_bw(),
            title = "Survival Curve by Alcohol Status"
)
```

```
## Warning in geom_segment(aes(x = 0, y = max(y2), xend = max(x1), yend = max(y2)), : All aesthetics ha
## i Please consider using `annotate()` or provide this layer with data containing
##   a single row.
## All aesthetics have length 1, but the data has 2 rows.
```

```
## i Please consider using `annotate()` or provide this layer with data containing
##   a single row.
## All aesthetics have length 1, but the data has 2 rows.
## i Please consider using `annotate()` or provide this layer with data containing
##   a single row.
## All aesthetics have length 1, but the data has 2 rows.
## i Please consider using `annotate()` or provide this layer with data containing
##   a single row.
```

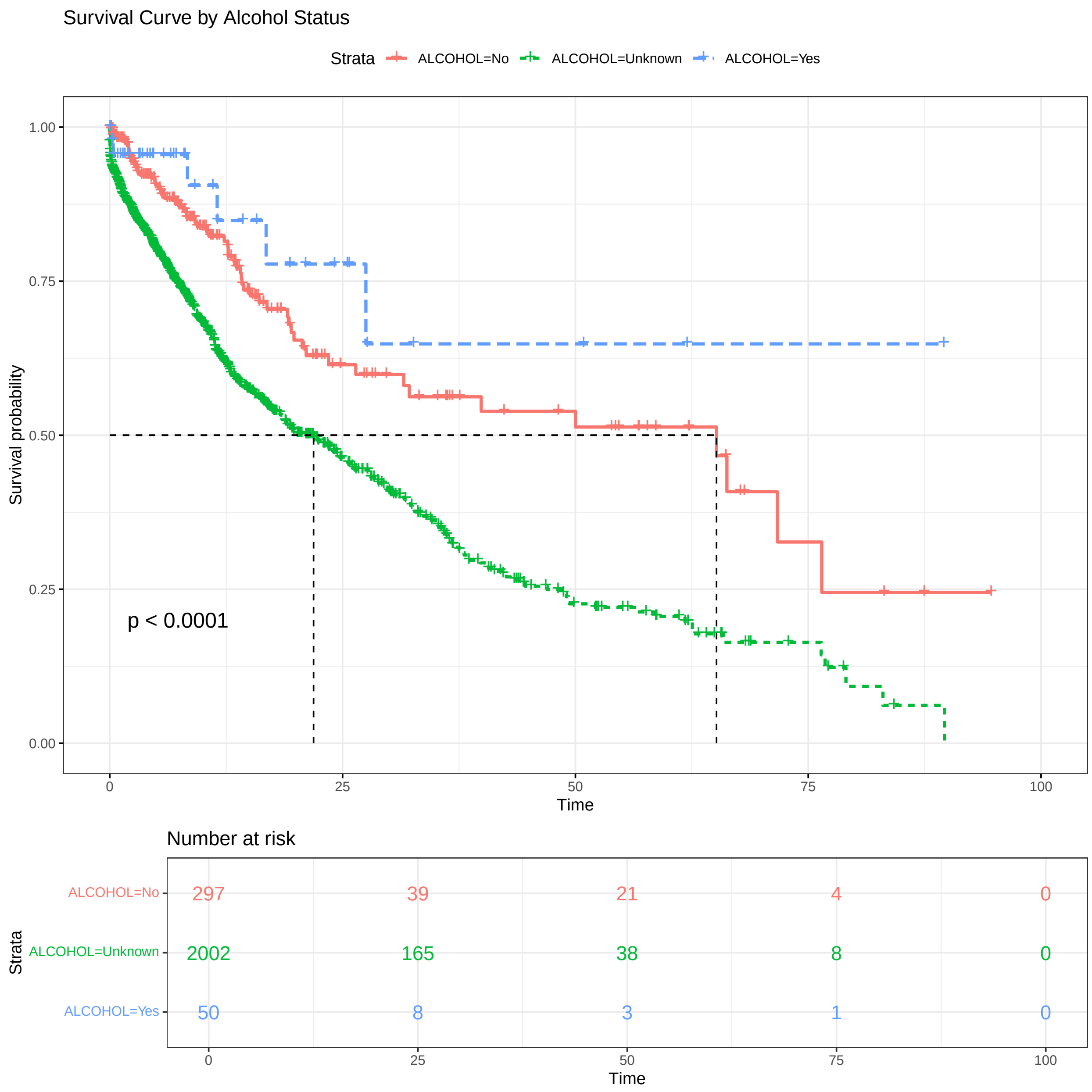


```r
ggsurvplot(km_ALCOHOL,
           conf.int = FALSE,
           risk.table.col = "strata",
           ggtheme = theme_bw(),
```

```
         fun = "cumhaz",
         title = "Cumulative Hazard Plot by Alcohol Status"
)
```

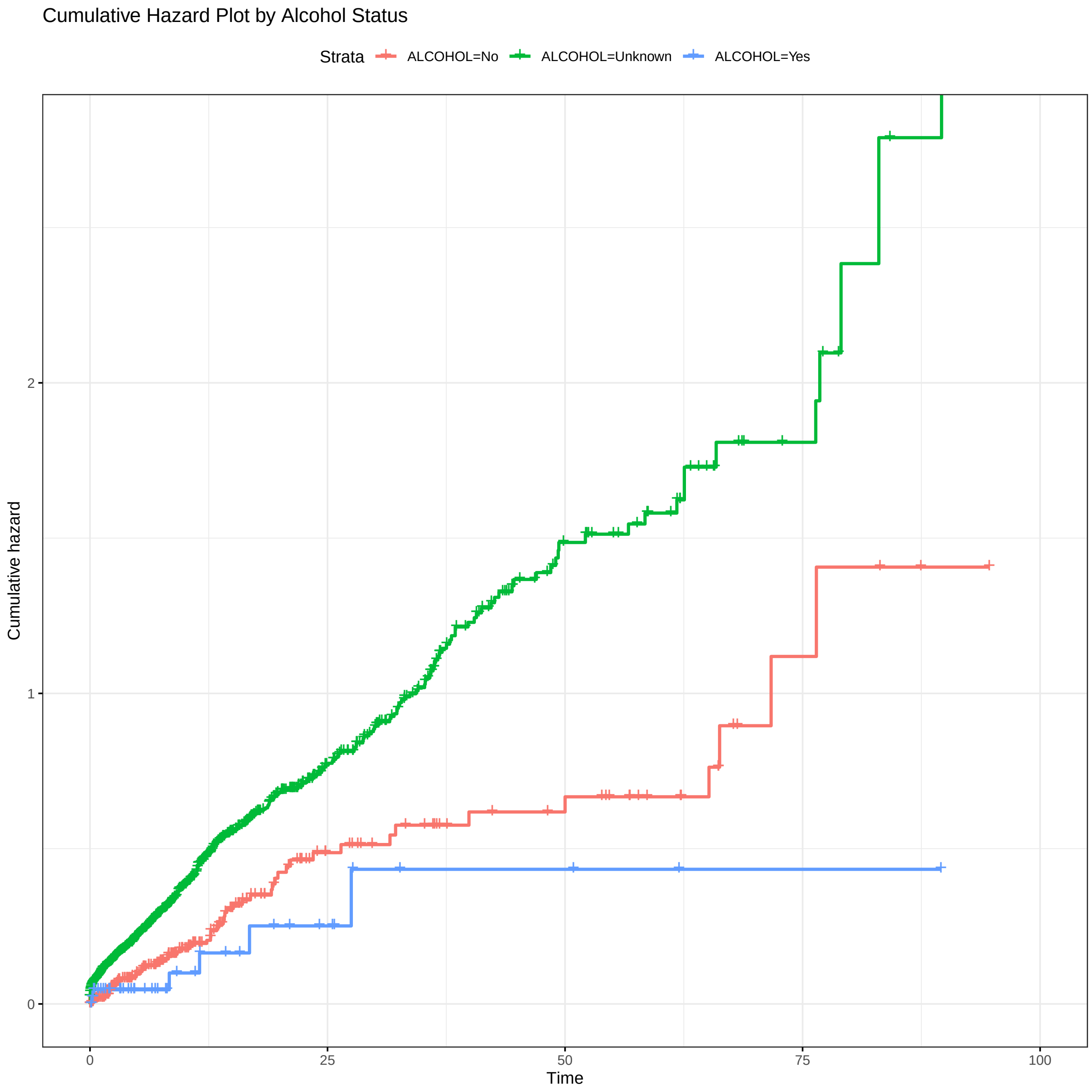


```
# Log-rank test for ALCOHOL_Group
ALCOHOLlrt <- survdiff(Surv(time2death, Censor) ~ ALCOHOL, data = cervical)

# View the test result
print(ALCOHOLlrt)
```

```
## Call:
## survdiff(formula = Surv(time2death, Censor) ~ ALCOHOL, data = cervical)
##
```

```
##                    N Observed Expected (O-E)^2/E (O-E)^2/V
## ALCOHOL=No       297       63    113.6     22.52     28.13
## ALCOHOL=Unknown 2002      534    471.6      8.25     38.48
## ALCOHOL=Yes       50        6     17.8      7.84      8.12
##
##  Chisq= 39.2  on 2 degrees of freedom, p= 3e-09
```

KM - HIVSTAT

```
# HIVSTAT
km_HIVSTAT <- survfit(Surv(time2death, Censor) ~ HIVSTAT, data = cervical)
summary(km_HIVSTAT)$table
```

```
##                  records n.max n.start events    rmean se(rmean)   median
## HIVSTAT=Negative     253   253     253     82 36.82012  3.301012 30.45585
## HIVSTAT=Positive    1014  1014    1014    309 35.32789  2.010169 24.70637
## HIVSTAT=Unknown     1082  1082    1082    212 35.08297  2.845835 22.80082
##                   0.95LCL  0.95UCL
## HIVSTAT=Negative 19.77823 37.05955
## HIVSTAT=Positive 20.10678 29.60164
## HIVSTAT=Unknown  16.09856 30.09446
```

```
dHIVSTAT <- data.frame(time = km_HIVSTAT$time,
                  n.risk = km_HIVSTAT$n.risk,
                  n.event = km_HIVSTAT$n.event,
                  n.censor = km_HIVSTAT$n.censor,
                  surv = km_HIVSTAT$surv,
                  upper = km_HIVSTAT$upper,
                  lower = km_HIVSTAT$lower
                  )
head(dHIVSTAT)
```

```
##         time n.risk n.event n.censor      surv     upper     lower
## 1 0.00000000    253       3        8 0.9881423 1.0000000 0.9748937
## 2 0.03285421    242       3        4 0.9758926 0.9951400 0.9570175
## 3 0.06570842    235       1        8 0.9717399 0.9926045 0.9513138
## 4 0.09856263    226       0        5 0.9717399 0.9926045 0.9513138
## 5 0.13141684    221       1        4 0.9673428 0.9898780 0.9453207
## 6 0.19712526    216       0        2 0.9673428 0.9898780 0.9453207
```

```
ggsurvplot(km_HIVSTAT,
           pval = TRUE, conf.int = FALSE,
           risk.table = TRUE,
           risk.table.col = "strata",
           linetype = "strata",
           surv.median.line = "hv",
           ggtheme = theme_bw(),
          title = "Survival Curve by HIV Status"
)
```

```
## Warning in geom_segment(aes(x = 0, y = max(y2), xend = max(x1), yend = max(y2)), : All aesthetics ha
## i Please consider using ‘annotate()‘ or provide this layer with data containing
##   a single row.
## All aesthetics have length 1, but the data has 3 rows.
## i Please consider using ‘annotate()‘ or provide this layer with data containing
##   a single row.
## All aesthetics have length 1, but the data has 3 rows.
## i Please consider using ‘annotate()‘ or provide this layer with data containing
##   a single row.
## All aesthetics have length 1, but the data has 3 rows.
## i Please consider using ‘annotate()‘ or provide this layer with data containing
##   a single row.
```

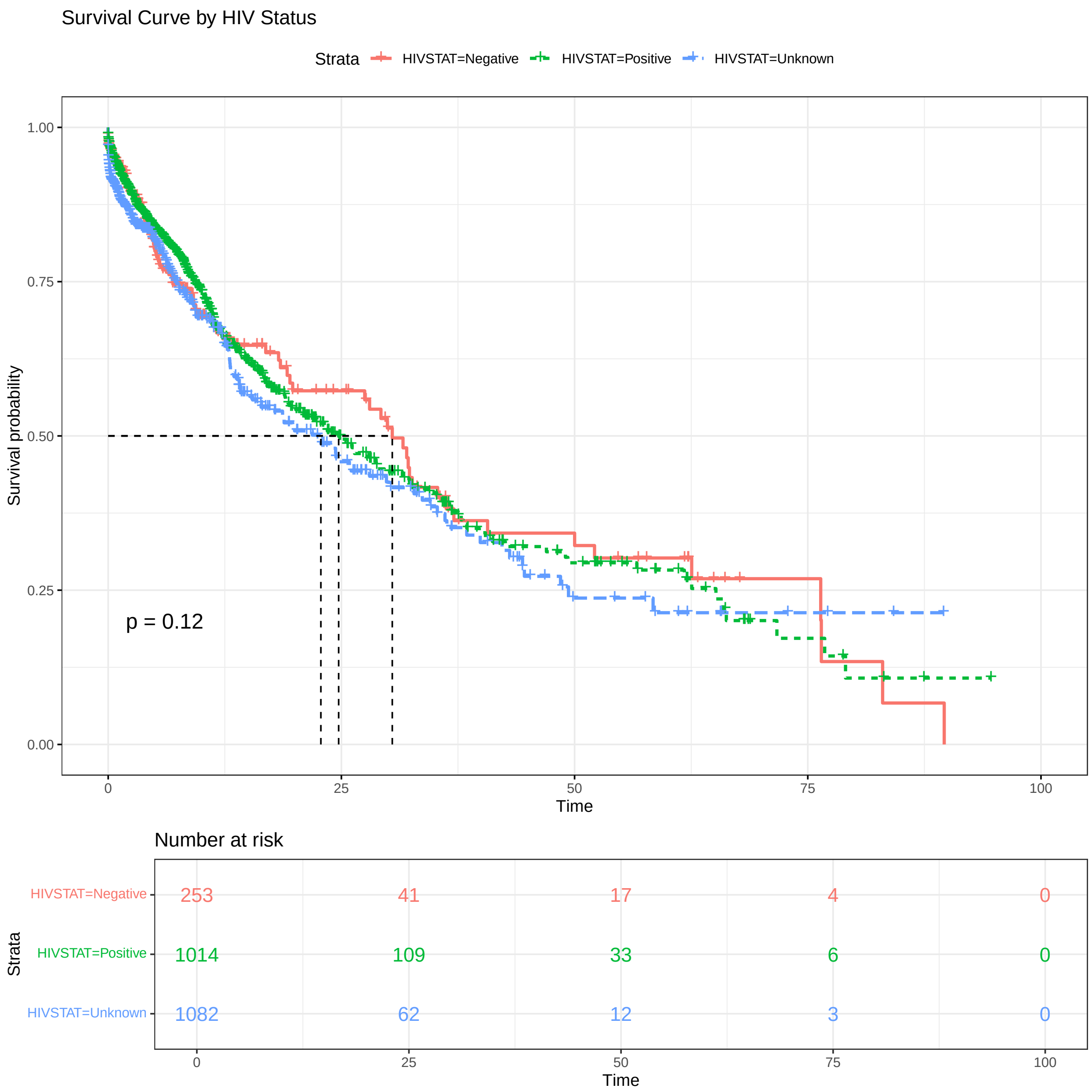

```r
ggsurvplot(km_HIVSTAT,
           conf.int = FALSE,
           risk.table.col = "strata",
           ggtheme = theme_bw(),
           fun = "cumhaz",
           title = "Cumulative Hazard Plot by HIV Status"
)
```

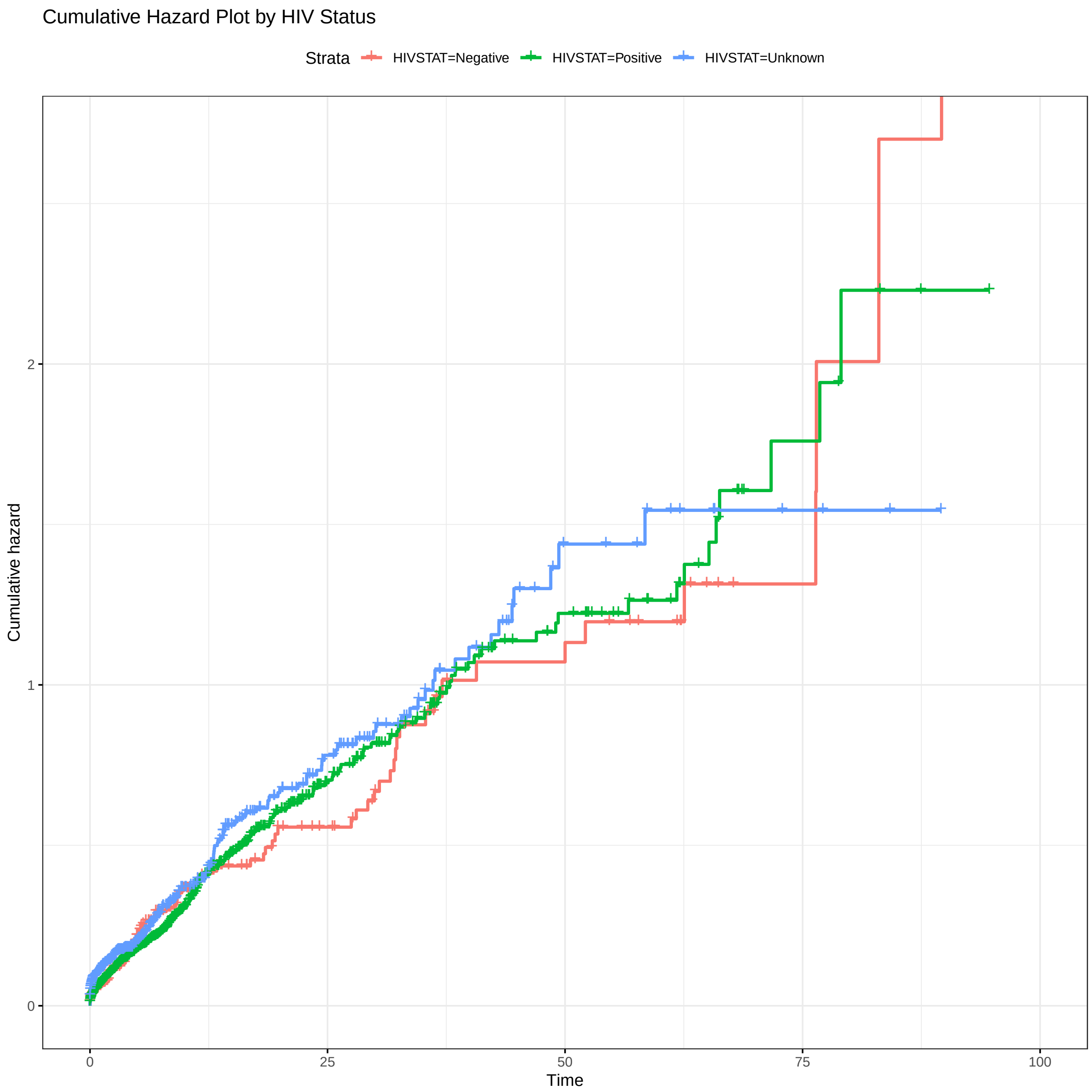


```r
# Log-rank test for HIVSTAT_Group
HIVSTATlrt <- survdiff(Surv(time2death, Censor) ~ HIVSTAT, data = cervical)

# View the test result
print(HIVSTATlrt)
```

```
## Call:
## survdiff(formula = Surv(time2death, Censor) ~ HIVSTAT, data = cervical)
##
##                     N Observed Expected (O-E)^2/E (O-E)^2/V
## HIVSTAT=Negative  253       82     87.6     0.357     0.423
## HIVSTAT=Positive 1014      309    326.6     0.946     2.089
## HIVSTAT=Unknown  1082      212    188.8     2.841     4.224
##
##  Chisq= 4.2  on 2 degrees of freedom, p= 0.1
```

KM - FAMILY HISTORY OF CANCER

```
# FAMHIST
km_FAMHIST <- survfit(Surv(time2death, Censor) ~ FAMHIST, data = cervical)
summary(km_FAMHIST)$table
```

```
##                 records n.max n.start events    rmean se(rmean)  median
## FAMHIST=No          253   253     253     54 52.34384  4.317692 65.1499
## FAMHIST=Unknown    2035  2035    2035    539 30.93075  1.401139 21.8809
## FAMHIST=Yes          61    61      61     10 53.73212 11.420430 31.5729
##                  0.95LCL  0.95UCL
## FAMHIST=No      39.88501       NA
## FAMHIST=Unknown 18.46407 25.56057
## FAMHIST=Yes     27.49897       NA
```

```
dFAMHIST <- data.frame(time = km_FAMHIST$time,
                  n.risk = km_FAMHIST$n.risk,
                  n.event = km_FAMHIST$n.event,
                  n.censor = km_FAMHIST$n.censor,
                  surv = km_FAMHIST$surv,
                  upper = km_FAMHIST$upper,
                  lower = km_FAMHIST$lower
                  )
head(dFAMHIST)
```

```
##         time n.risk n.event n.censor      surv upper     lower
## 1 0.00000000    253       1        4 0.9960474     1 0.9883458
## 2 0.03285421    248       0        1 0.9960474     1 0.9883458
## 3 0.06570842    247       0        2 0.9960474     1 0.9883458
## 4 0.09856263    245       0        1 0.9960474     1 0.9883458
## 5 0.16427105    244       1        3 0.9919653     1 0.9809347
## 6 0.19712526    240       0        1 0.9919653     1 0.9809347
```

```
ggsurvplot(km_FAMHIST,
           pval = TRUE, conf.int = FALSE,
           risk.table = TRUE,
           risk.table.col = "strata",
           linetype = "strata",
           surv.median.line = "hv",
           ggtheme = theme_bw(),
            title = "Survival Curve by Family History of Cancer"
)
```

```
## Warning in geom_segment(aes(x = 0, y = max(y2), xend = max(x1), yend = max(y2)), : All aesthetics ha
## i Please consider using ‘annotate()‘ or provide this layer with data containing
##   a single row.
## All aesthetics have length 1, but the data has 3 rows.
## i Please consider using ‘annotate()‘ or provide this layer with data containing
##   a single row.
## All aesthetics have length 1, but the data has 3 rows.
## i Please consider using ‘annotate()‘ or provide this layer with data containing
##   a single row.
## All aesthetics have length 1, but the data has 3 rows.
## i Please consider using ‘annotate()‘ or provide this layer with data containing
##   a single row.
```

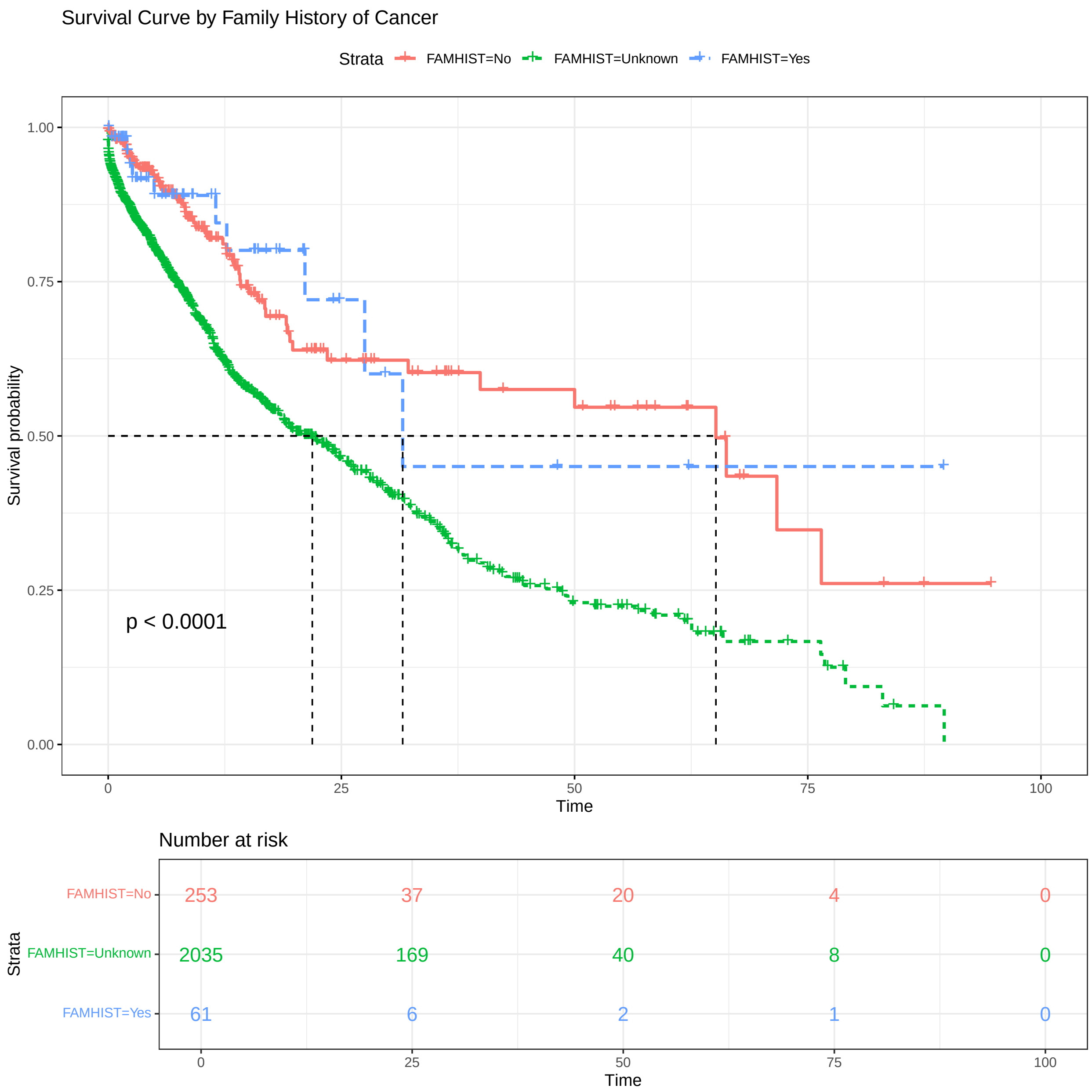

```r
ggsurvplot(km_FAMHIST,
          conf.int = FALSE,
          risk.table.col = "strata",
          ggtheme = theme_bw(),
          fun = "cumhaz",
          title = "Cumulative Hazard Plot by Family History of Cancer"
)
```

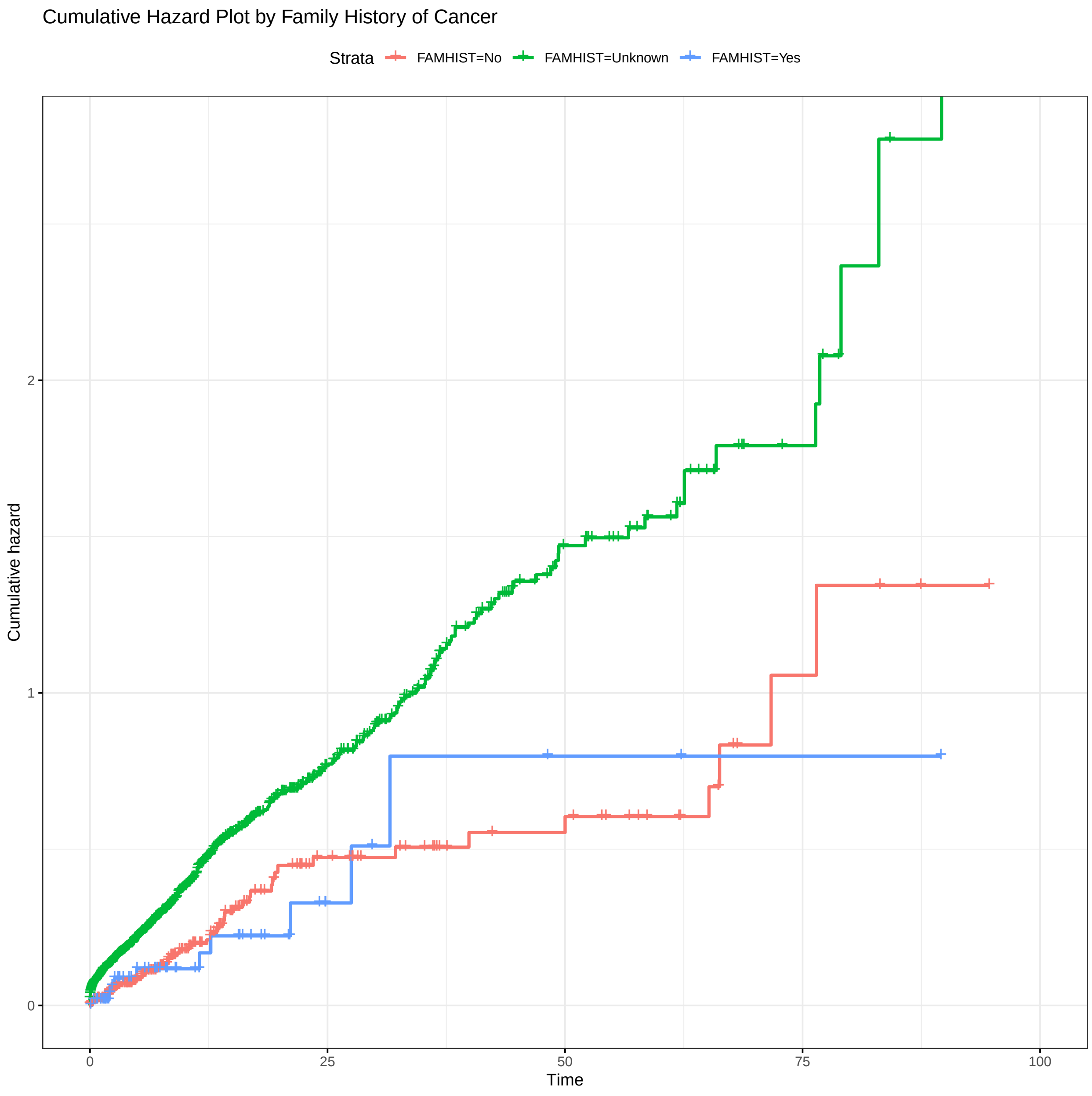


```r
# Log-rank test for FAMHIST_Group
FAMHISTlrt <- survdiff(Surv(time2death, Censor) ~ FAMHIST, data = cervical)

# View the test result
print(FAMHISTlrt)
```

```
## Call:
## survdiff(formula = Surv(time2death, Censor) ~ FAMHIST, data = cervical)
##
##                    N Observed Expected (O-E)^2/E (O-E)^2/V
## FAMHIST=No       253       54    101.8     22.45     27.46
## FAMHIST=Unknown 2035      539    479.4      7.41     36.74
## FAMHIST=Yes       61       10     21.8      6.37      6.64
##
##  Chisq= 36.8  on 2 degrees of freedom, p= 1e-08
```

KM - TREATMENT

```
# TRT
km_TRT <- survfit(Surv(time2death, Censor) ~ TRT, data = cervical)
summary(km_TRT)$table
```

```
##          records n.max n.start events    rmean se(rmean)   median  0.95LCL
## TRT=No      1387  1387    1387    304 30.04345  2.325608 18.85832 13.99589
## TRT=Yes      962   962     962    299 38.76700  1.916132 28.71458 23.85216
##           0.95UCL
## TRT=No   25.85626
## TRT=Yes  35.21971
```

```
dTRT <- data.frame(time = km_TRT$time,
                  n.risk = km_TRT$n.risk,
                  n.event = km_TRT$n.event,
                  n.censor = km_TRT$n.censor,
                  surv = km_TRT$surv,
                  upper = km_TRT$upper,
                  lower = km_TRT$lower
                  )
head(dTRT)
```

```
##         time n.risk n.event n.censor      surv     upper     lower
## 1 0.00000000   1387      40      273 0.9711608 0.9800082 0.9623932
## 2 0.03285421   1074      16       49 0.9566928 0.9679290 0.9455871
## 3 0.06570842   1009       6       36 0.9510039 0.9630758 0.9390833
## 4 0.09856263    967       3       20 0.9480535 0.9605497 0.9357198
## 5 0.13141684    944       2       16 0.9460449 0.9588270 0.9334332
## 6 0.16427105    926       6       19 0.9399150 0.9535414 0.9264834
```

```
ggsurvplot(km_TRT,
          pval = TRUE, conf.int = FALSE,
          risk.table = TRUE,
          risk.table.col = "strata",
          linetype = "strata",
          surv.median.line = "hv",
          ggtheme = theme_bw(),
           title = "Survival Curve by Treatment"
)
```

```
## Warning in geom_segment(aes(x = 0, y = max(y2), xend = max(x1), yend = max(y2)), : All aesthetics ha
## i Please consider using `annotate()` or provide this layer with data containing
##   a single row.
## All aesthetics have length 1, but the data has 2 rows.
## i Please consider using `annotate()` or provide this layer with data containing
##   a single row.
## All aesthetics have length 1, but the data has 2 rows.
## i Please consider using `annotate()` or provide this layer with data containing
##   a single row.
## All aesthetics have length 1, but the data has 2 rows.
## i Please consider using `annotate()` or provide this layer with data containing
##   a single row.
```

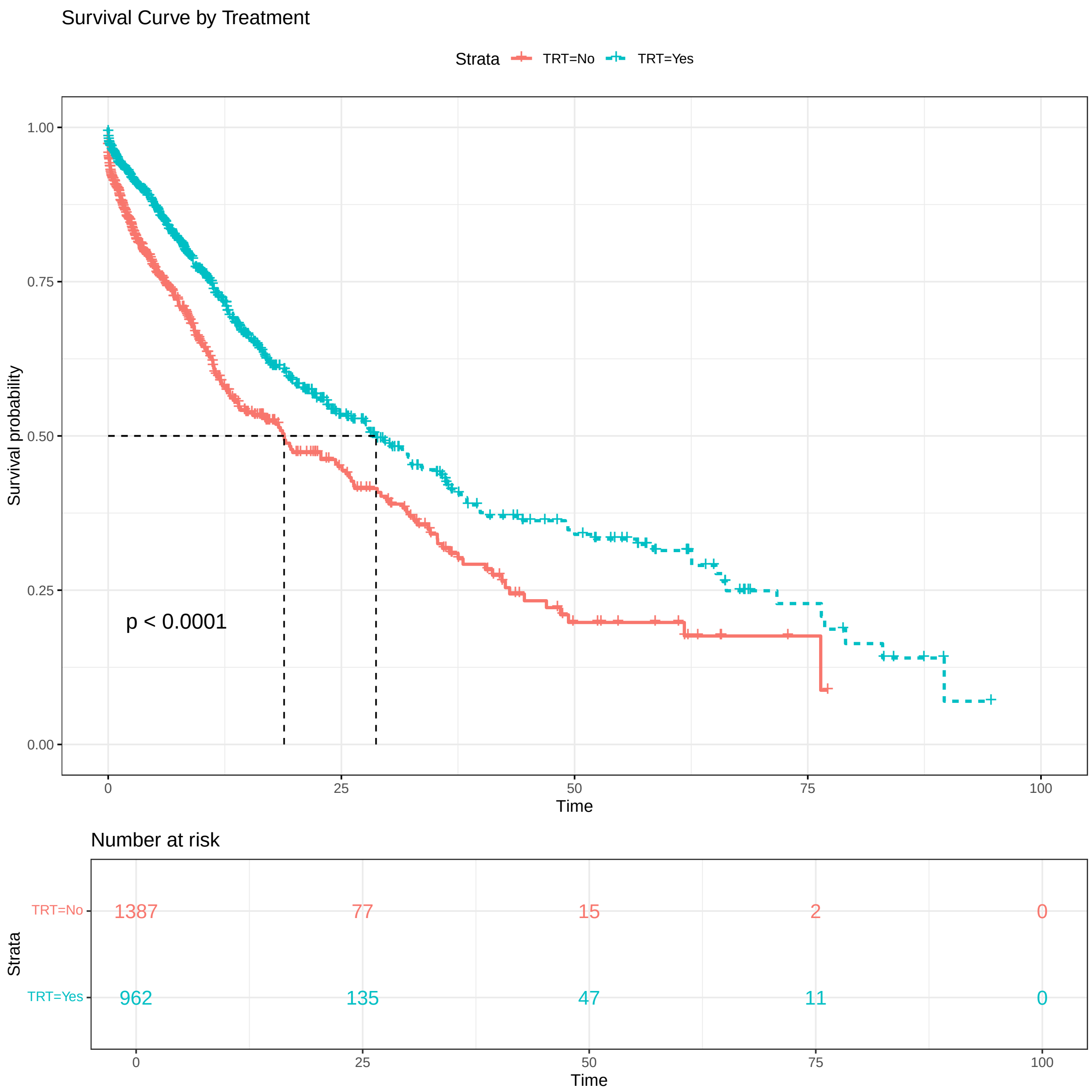

```r
ggsurvplot(km_TRT,
           conf.int = FALSE,
           risk.table.col = "strata",
           ggtheme = theme_bw(),
           fun = "cumhaz",
           title = "Cumulative Hazard Plot by Treatment"
)
```

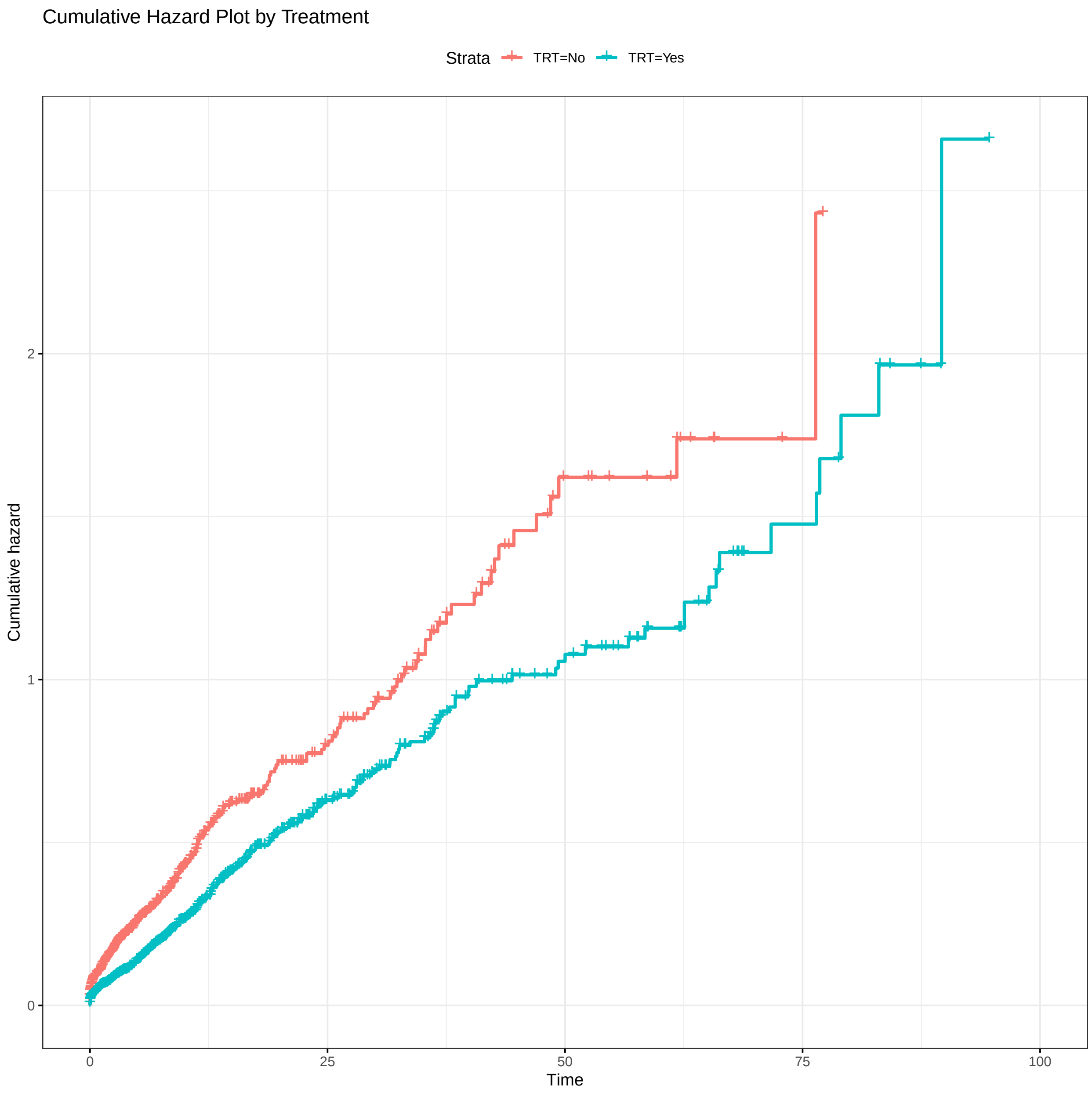


```r
# Log-rank test for TRT_Group
TRTlrt <- survdiff(Surv(time2death, Censor) ~ TRT, data = cervical)

# View the test result
print(TRTlrt)
```

```
## Call:
## survdiff(formula = Surv(time2death, Censor) ~ TRT, data = cervical)
##
##             N Observed Expected (O-E)^2/E (O-E)^2/V
## TRT=No  1387      304      242      15.7      27.3
## TRT=Yes  962      299      361      10.5      27.3
##
##  Chisq= 27.3  on 1 degrees of freedom, p= 2e-07
```

KM - BASIS OF DIAGNOSIS

```
# BAS
km_BAS <- survfit(Surv(time2death, Censor) ~ BAS, data = cervical)
summary(km_BAS)$table
```

```
##                           records n.max n.start events    rmean se(rmean)
## BAS=Clinical                  565   565     565    195 27.11759  2.905438
## BAS=Histology of Primary     1784  1784    1784    408 36.93832  1.618050
##                             median   0.95LCL  0.95UCL
## BAS=Clinical              9.166324  6.439425 13.99589
## BAS=Histology of Primary 28.714579 24.640657 32.45996
```

```
dBAS <- data.frame(time = km_BAS$time,
                   n.risk = km_BAS$n.risk,
                   n.event = km_BAS$n.event,
                   n.censor = km_BAS$n.censor,
                   surv = km_BAS$surv,
                   upper = km_BAS$upper,
                   lower = km_BAS$lower
                   )
head(dBAS)
```

```
##         time n.risk n.event n.censor      surv     upper     lower
## 1 0.00000000    565      31       75 0.9451327 0.9640975 0.9265410
## 2 0.03285421    459      20       35 0.9039505 0.9294854 0.8791171
## 3 0.06570842    404       7       26 0.8882880 0.9159985 0.8614157
## 4 0.09856263    371       5       29 0.8763164 0.9056637 0.8479202
## 5 0.13141684    337       1       14 0.8737161 0.9034378 0.8449722
## 6 0.16427105    322       7       16 0.8547223 0.8871202 0.8235075
```

```
ggsurvplot(km_BAS,
           pval = TRUE, conf.int = FALSE,
           risk.table = TRUE,
           risk.table.col = "strata",
           linetype = "strata",
           surv.median.line = "hv",
           ggtheme = theme_bw(),
            title = "Survival Curve by Basis of Diagnosis"
)
```

```
## Warning in geom_segment(aes(x = 0, y = max(y2), xend = max(x1), yend = max(y2)), : All aesthetics ha
## i Please consider using `annotate()` or provide this layer with data containing
##   a single row.
## All aesthetics have length 1, but the data has 2 rows.
## i Please consider using `annotate()` or provide this layer with data containing
##   a single row.
## All aesthetics have length 1, but the data has 2 rows.
## i Please consider using `annotate()` or provide this layer with data containing
##   a single row.
## All aesthetics have length 1, but the data has 2 rows.
## i Please consider using `annotate()` or provide this layer with data containing
##   a single row.
```

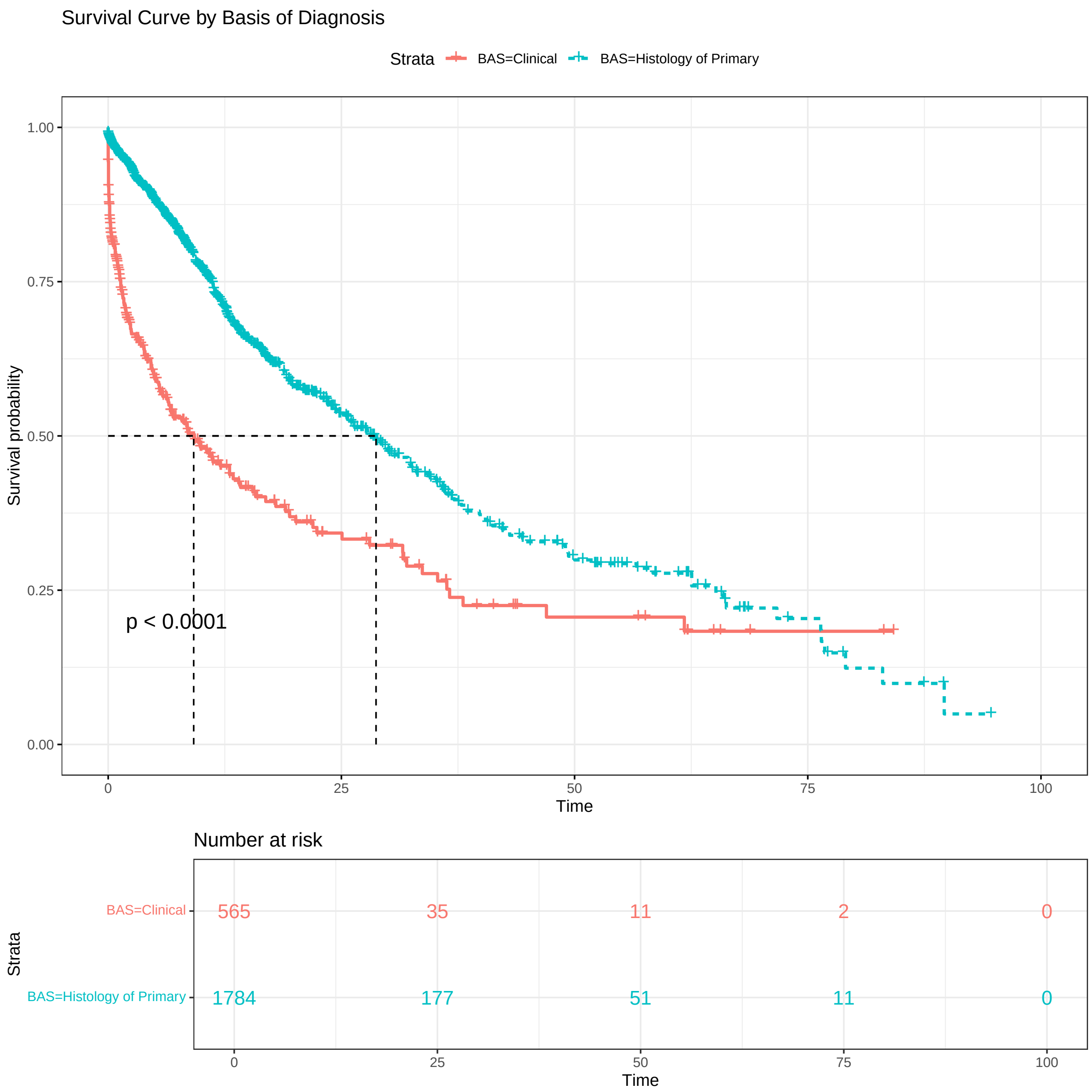

```r
ggsurvplot(km_HIVSTAT,
          conf.int = FALSE,
          risk.table.col = "strata",
          ggtheme = theme_bw(),
          fun = "cumhaz",
          title = "Cumulative Hazard Plot by Basis of Diagnosis"
)
```

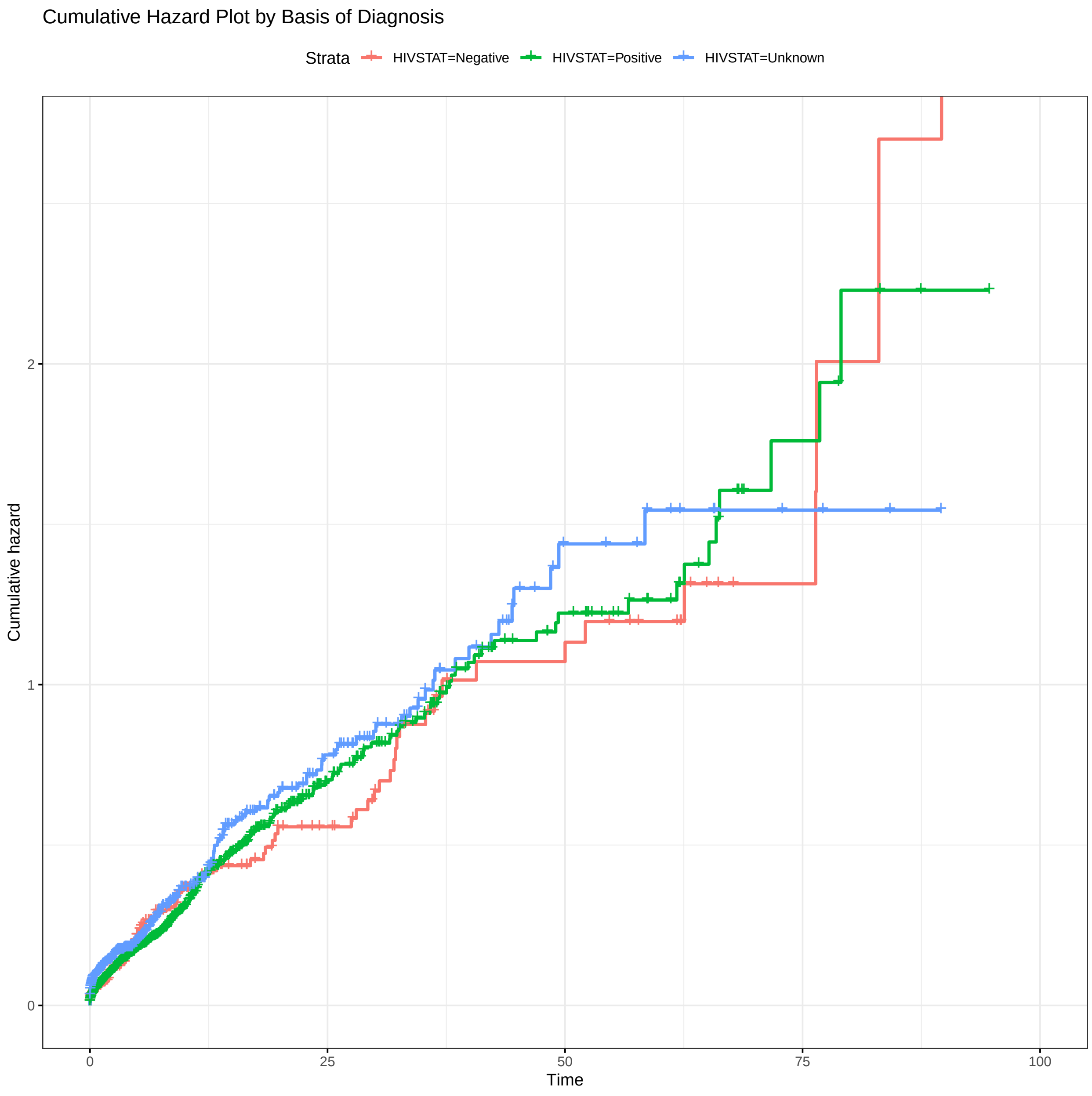


```r
# Log-rank test for BAS_Group
BASlrt <- survdiff(Surv(time2death, Censor) ~ BAS, data = cervical)

# View the test result
print(BASlrt)
```

```
## Call:
## survdiff(formula = Surv(time2death, Censor) ~ BAS, data = cervical)
##
##                              N Observed Expected (O-E)^2/E (O-E)^2/V
## BAS=Clinical               565      195      100      90.4       109
## BAS=Histology of Primary 1784      408      503      18.0       109
##
##  Chisq= 110  on 1 degrees of freedom, p= <2e-16
```

UNIVARIATE - COX PH MODEL

```
# Create an empty list to store results
results_list <- list()

# List of covariates
covariates <- c("Age_Group", "STAGE", "SMOKE", "ALCOHOL", "HIVSTAT", "FAMHIST", "TRT", "BAS")

# Loop through each covariate
for (cov in covariates) {
  # Fit the Cox model
  model <- coxph(as.formula(paste("Surv(time2death, Censor) ~", cov)), data = cervical)
  model_summary <- summary(model)

  # Extract relevant statistics
  beta <- signif(model_summary$coef[1], digits = 2)
  HR <- signif(model_summary$coef[2], digits = 2)
  HR_lower <- signif(model_summary$conf.int[,"lower .95"], 2)
  HR_upper <- signif(model_summary$conf.int[,"upper .95"], 2)
  p_value <- signif(model_summary$wald["pvalue"], digits = 2)
  wald_test <- signif(model_summary$wald["test"], digits = 2)

  # Combine HR with confidence intervals
  HR_combined <- paste0(HR, " (", HR_lower, "-", HR_upper, ")")

  # Store results as a named vector
  results_list[[cov]] <- c(beta = beta, `HR (95% CI)` = HR_combined, `Wald Test` = wald_test, `P-Value`
}

# Convert the list to a data frame
results_table <- do.call(rbind, results_list)
```

```
## Warning in (function (..., deparse.level = 1) : number of columns of result is
## not a multiple of vector length (arg 1)
```

```
# Convert to a data frame for better readability
results_table <- as.data.frame(results_table)

# Print the results
print(results_table)
```

```
##            beta      HR (95% CI)1      HR (95% CI)2   HR (95% CI)3
```

```
## Age_Group  0.16 -0.056 (0.95-1.4) -0.056 (0.76-1.2)            5.6
## STAGE      0.43    0.94 (1.1-2.2)    0.94 (1.8-3.6) 0.94 (2.9-5.8)
## SMOKE      0.71   -0.31 (1.6-2.6)   -0.31 (0.3-1.8)             34
## ALCOHOL    0.72    -0.5 (1.6-2.7)    -0.5 (0.26-1.4)            37
## HIVSTAT   0.011    0.19 (0.79-1.3)   0.19 (0.93-1.6)            4.3
## FAMHIST    0.76   -0.14 (1.6-2.8)  -0.14 (0.44-1.7)             35
## TRT       -0.43  0.65 (0.55-0.76)                27          2e-07
## BAS       -0.89  0.41 (0.35-0.49)               100        2.9e-24
##             HR (95% CI)4   Wald Test.test    P-Value.pvalue
## Age_Group          0.059             0.16 -0.056 (0.95-1.4)
## STAGE      0.94 (1.8-3.7)              98           2.2e-20
## SMOKE            3.9e-08             0.71   -0.31 (1.6-2.6)
## ALCOHOL          9.4e-09             0.72    -0.5 (1.6-2.7)
## HIVSTAT             0.12            0.011   0.19 (0.79-1.3)
## FAMHIST          2.5e-08             0.76   -0.14 (1.6-2.8)
## TRT                -0.43 0.65 (0.55-0.76)                27
## BAS                -0.89 0.41 (0.35-0.49)               100
```

CHECK PH ASSUMPTION

```
# Create a list to store results
ph_test_results <- list()

# Loop through each covariate
for (cov in covariates) {
  # Fit Cox model for the current covariate
  model <- coxph(as.formula(paste("Surv(time2death, Censor) ~", cov)), data = cervical)

  # Perform the proportional hazards test
  ph_test <- cox.zph(model)

  # Store the result in the list
  ph_test_results[[cov]] <- ph_test

  # Print the results
  cat("\nProportional Hazards Test for:", cov, "\n")
  print(ph_test)
}
```

```
##
## Proportional Hazards Test for: Age_Group
##           chisq df   p
## Age_Group  2.38  2 0.3
## GLOBAL     2.38  2 0.3
##
## Proportional Hazards Test for: STAGE
##        chisq df       p
## STAGE   53.5  4 6.7e-11
## GLOBAL  53.5  4 6.7e-11
##
## Proportional Hazards Test for: SMOKE
##        chisq df    p
```

```
## SMOKE  0.897  2 0.64
## GLOBAL 0.897  2 0.64
##
## Proportional Hazards Test for: ALCOHOL
##         chisq df    p
## ALCOHOL 0.561  2 0.76
## GLOBAL  0.561  2 0.76
##
## Proportional Hazards Test for: HIVSTAT
##         chisq df    p
## HIVSTAT  3.96  2 0.14
## GLOBAL   3.96  2 0.14
##
## Proportional Hazards Test for: FAMHIST
##         chisq df    p
## FAMHIST 0.235  2 0.89
## GLOBAL  0.235  2 0.89
##
## Proportional Hazards Test for: TRT
##        chisq df     p
## TRT     4.42  1 0.036
## GLOBAL  4.42  1 0.036
##
## Proportional Hazards Test for: BAS
##        chisq df       p
## BAS     63.7  1 1.5e-15
## GLOBAL  63.7  1 1.5e-15
```

STAGE MODEL - MULTIVARIATE COX PH

IMPLEMENTING THE 10% RULE FOR CONFOUNDING

CRUDE MOEL

```
# Crude Cox model
crude_model <- coxph(Surv(time2death, Censor) ~ STAGE, data = cervical)
summary(crude_model)
```

```
## Call:
## coxph(formula = Surv(time2death, Censor) ~ STAGE, data = cervical)
##
##   n= 2349, number of events= 603
##
##                              coef exp(coef) se(coef)     z Pr(>|z|)
## STAGEStage 2               0.4313    1.5392   0.1831 2.355   0.0185 *
## STAGEStage 3               0.9433    2.5685   0.1772 5.322 1.02e-07 ***
## STAGEStage 4               1.4160    4.1205   0.1784 7.938 2.06e-15 ***
## STAGEStage not determined 0.9430    2.5677   0.1799 5.241 1.60e-07 ***
## ---
## Signif. codes:  0 '***' 0.001 '**' 0.01 '*' 0.05 '.' 0.1 ' ' 1
##
```

```
##                              exp(coef) exp(-coef) lower .95 upper .95
## STAGEStage 2                     1.539     0.6497     1.075     2.204
## STAGEStage 3                     2.569     0.3893     1.815     3.635
## STAGEStage 4                     4.121     0.2427     2.905     5.845
## STAGEStage not determined        2.568     0.3895     1.805     3.653
##
## Concordance= 0.648  (se = 0.012 )
## Likelihood ratio test= 104.2  on 4 df,   p=<2e-16
## Wald test            = 98.4  on 4 df,   p=<2e-16
## Score (logrank) test = 106.3  on 4 df,   p=<2e-16
```

```
# Extract the crude hazard ratio (cHR)
crude_hr <- exp(coef(crude_model))
```

LIST OF POTENTTIAL CONFOUNDERS

```
# List of potential confounders
confounders <- c("Age_Group", "SMOKE", "ALCOHOL", "HIVSTAT", "FAMHIST", "TRT", "BAS")
```

10% RULE

```
# Store results
confounding_results <- data.frame(StageLevel = character(), Confounder = character(), PercentDifference

# Loop through each potential confounder
for (confounder in confounders) {
  # Adjusted model with the confounder
  model <- coxph(as.formula(paste("Surv(time2death, Censor) ~ STAGE +", confounder)), data = cervical)

  # Extract coefficients for all levels of STAGE
  stage_levels <- grep("^STAGE", names(coef(model)), value = TRUE)

  for (level in stage_levels) {
    # Crude HR for the specific level of STAGE
    crude_hr <- exp(coef(crude_model)[level])

    # Adjusted HR for the specific level of STAGE
    adjusted_hr <- exp(coef(model)[level])

    # Calculate percent difference
    percent_diff <- abs((adjusted_hr - crude_hr) / crude_hr) * 100

    # Check if confounding exists
    confounding <- ifelse(percent_diff > 10, "Yes", "No")

    # Append results
    confounding_results <- rbind(confounding_results, data.frame(
      StageLevel = level,
      Confounder = confounder,
      PercentDifference = percent_diff,
```

```r
      Confounding = confounding
    ))
  }
}

# Print results
print(confounding_results)
```

```
##                                              StageLevel Confounder
## STAGEStage 2                               STAGEStage 2  Age_Group
## STAGEStage 3                               STAGEStage 3  Age_Group
## STAGEStage 4                               STAGEStage 4  Age_Group
## STAGEStage not determined  STAGEStage not determined  Age_Group
## STAGEStage 21                              STAGEStage 2      SMOKE
## STAGEStage 31                              STAGEStage 3      SMOKE
## STAGEStage 41                              STAGEStage 4      SMOKE
## STAGEStage not determined1 STAGEStage not determined      SMOKE
## STAGEStage 22                              STAGEStage 2    ALCOHOL
## STAGEStage 32                              STAGEStage 3    ALCOHOL
## STAGEStage 42                              STAGEStage 4    ALCOHOL
## STAGEStage not determined2 STAGEStage not determined    ALCOHOL
## STAGEStage 23                              STAGEStage 2    HIVSTAT
## STAGEStage 33                              STAGEStage 3    HIVSTAT
## STAGEStage 43                              STAGEStage 4    HIVSTAT
## STAGEStage not determined3 STAGEStage not determined    HIVSTAT
## STAGEStage 24                              STAGEStage 2    FAMHIST
## STAGEStage 34                              STAGEStage 3    FAMHIST
## STAGEStage 44                              STAGEStage 4    FAMHIST
## STAGEStage not determined4 STAGEStage not determined    FAMHIST
## STAGEStage 25                              STAGEStage 2        TRT
## STAGEStage 35                              STAGEStage 3        TRT
## STAGEStage 45                              STAGEStage 4        TRT
## STAGEStage not determined5 STAGEStage not determined        TRT
## STAGEStage 26                              STAGEStage 2        BAS
## STAGEStage 36                              STAGEStage 3        BAS
## STAGEStage 46                              STAGEStage 4        BAS
## STAGEStage not determined6 STAGEStage not determined        BAS
##                            PercentDifference Confounding
## STAGEStage 2                      0.04444671          No
## STAGEStage 3                      0.42917811          No
## STAGEStage 4                      1.04284226          No
## STAGEStage not determined         1.72682147          No
## STAGEStage 21                     1.96223558          No
## STAGEStage 31                     2.88530675          No
## STAGEStage 41                     0.31505502          No
## STAGEStage not determined1       11.64336581         Yes
## STAGEStage 22                     1.77380471          No
## STAGEStage 32                     3.04530809          No
## STAGEStage 42                     0.49676176          No
## STAGEStage not determined2       11.44700430         Yes
## STAGEStage 23                     0.90087567          No
## STAGEStage 33                     0.87195605          No
## STAGEStage 43                     2.13940666          No
```

```
## STAGEStage not determined3        3.74949457          No
## STAGEStage 24                     2.37711728          No
## STAGEStage 34                     4.10614031          No
## STAGEStage 44                     0.38264999          No
## STAGEStage not determined4       11.60503577         Yes
## STAGEStage 25                     0.36650754          No
## STAGEStage 35                     3.71036915          No
## STAGEStage 45                     1.50069745          No
## STAGEStage not determined5       11.93141269         Yes
## STAGEStage 26                     0.84579208          No
## STAGEStage 36                     8.26472544          No
## STAGEStage 46                     9.42638611          No
## STAGEStage not determined6       20.33648348         Yes
```

STAGE MODEL - MULTIVARIATE COX PH

```r
## STAGE MODEL - MULTIVARIATE COX PH (Including STAGE)
stagecox <- coxph(Surv(time2death, Censor) ~ STAGE + Age_Group + SMOKE + HIVSTAT + TRT + BAS, data = ce

# Summary of the model
summary(stagecox)
```

```
## Call:
## coxph(formula = Surv(time2death, Censor) ~ STAGE + Age_Group +
##     SMOKE + HIVSTAT + TRT + BAS, data = cervical)
##
##   n= 2349, number of events= 603
##
##                               coef exp(coef) se(coef)      z Pr(>|z|)
## STAGEStage 2               0.39973   1.49142  0.18325  2.181  0.02916 *
## STAGEStage 3               0.81965   2.26970  0.17812  4.602 4.19e-06 ***
## STAGEStage 4               1.30643   3.69298  0.17955  7.276 3.44e-13 ***
## STAGEStage not determined  0.56989   1.76807  0.18429  3.092  0.00199 **
## Age_Group>50               0.08536   1.08911  0.10918  0.782  0.43430
## Age_Group40-49            -0.08428   0.91917  0.11475 -0.734  0.46267
## SMOKEUnknown               0.53010   1.69911  0.13780  3.847  0.00012 ***
## SMOKEYes                  -0.24053   0.78621  0.46598 -0.516  0.60574
## HIVSTATPositive            0.14573   1.15688  0.13078  1.114  0.26514
## HIVSTATUnknown             0.12073   1.12832  0.13717  0.880  0.37878
## TRTYes                    -0.20066   0.81819  0.08866 -2.263  0.02361 *
## BASHistology of Primary   -0.70584   0.49369  0.09166 -7.700 1.36e-14 ***
## ---
## Signif. codes:  0 '***' 0.001 '**' 0.01 '*' 0.05 '.' 0.1 ' ' 1
##
##                           exp(coef) exp(-coef) lower .95 upper .95
## STAGEStage 2                 1.4914     0.6705    1.0414    2.1359
## STAGEStage 3                 2.2697     0.4406    1.6009    3.2180
## STAGEStage 4                 3.6930     0.2708    2.5974    5.2506
## STAGEStage not determined    1.7681     0.5656    1.2321    2.5372
## Age_Group>50                 1.0891     0.9182    0.8793    1.3490
## Age_Group40-49               0.9192     1.0879    0.7340    1.1510
## SMOKEUnknown                 1.6991     0.5885    1.2969    2.2260
```

```
## SMOKEYes                       0.7862     1.2719    0.3154    1.9597
## HIVSTATPositive                1.1569     0.8644    0.8953    1.4949
## HIVSTATUnknown                 1.1283     0.8863    0.8623    1.4763
## TRTYes                         0.8182     1.2222    0.6877    0.9735
## BASHistology of Primary        0.4937     2.0255    0.4125    0.5909
##
## Concordance= 0.728  (se = 0.012 )
## Likelihood ratio test= 211.4  on 12 df,   p=<2e-16
## Wald test            = 212.8  on 12 df,   p=<2e-16
## Score (logrank) test = 230.2  on 12 df,   p=<2e-16
```

```
# Plot the baseline survival function by STAGE
ggsurvplot(
  survfit(stagecox),
  data = cervical,
  ggtheme = theme_minimal(),
  legend.title = "STAGE",
  risk.table = TRUE
)
```

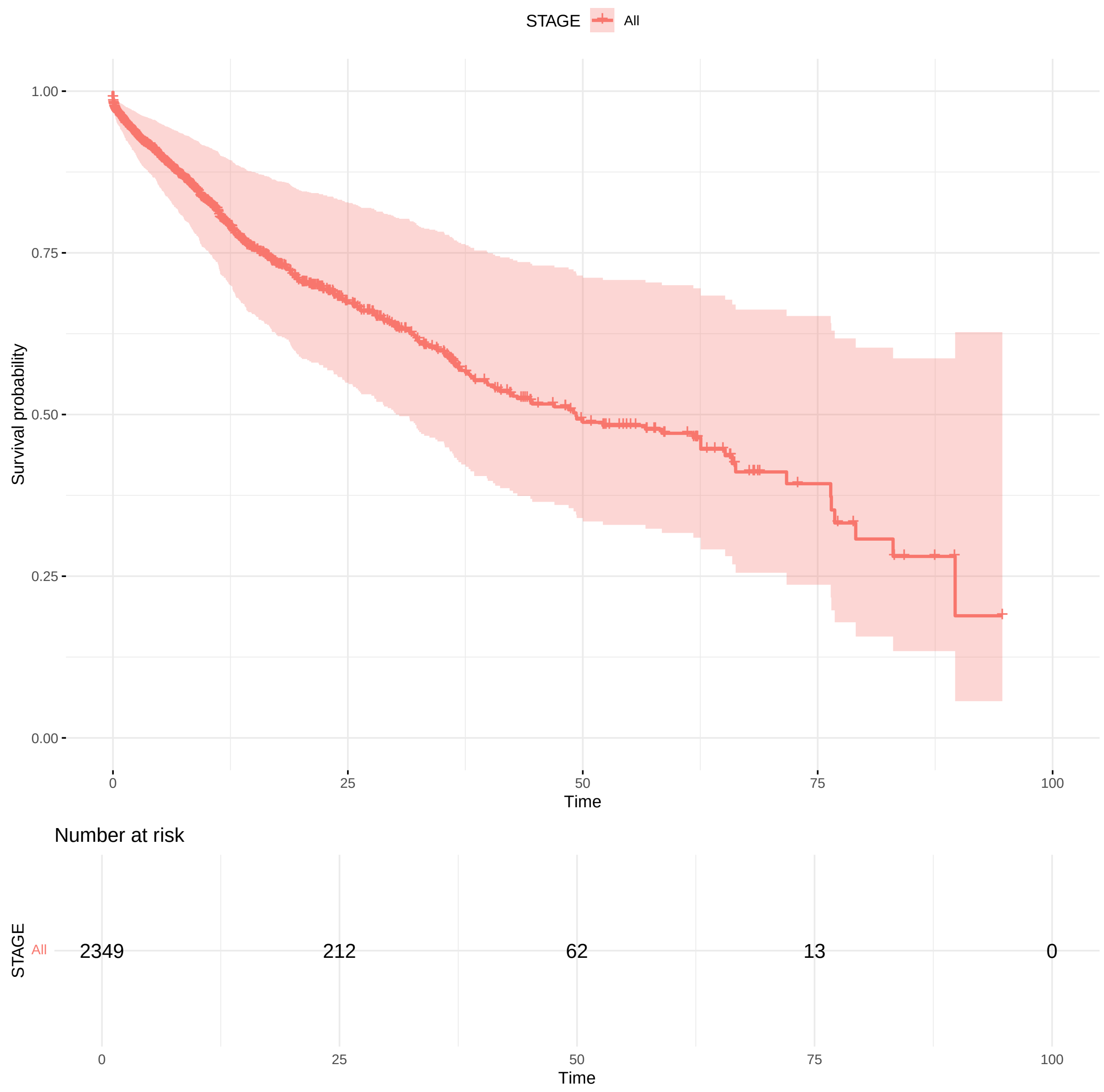

STAGE MODEL - CHECKING ASSUMPTIONS

```r
# Test PH assumption
stage.ph <- cox.zph(stagecox)
stage.ph
```

```
##             chisq df       p
## STAGE       61.78  4 1.2e-12
## Age_Group    4.08  2  0.1303
## SMOKE        1.47  2  0.4794
## HIVSTAT      6.97  2  0.0307
## TRT          8.13  1  0.0044
```

```
## BAS          59.68  1 1.1e-14
## GLOBAL      106.32 12 < 2e-16
```

```r
# Graph PH
ggcoxzph(stage.ph)
```

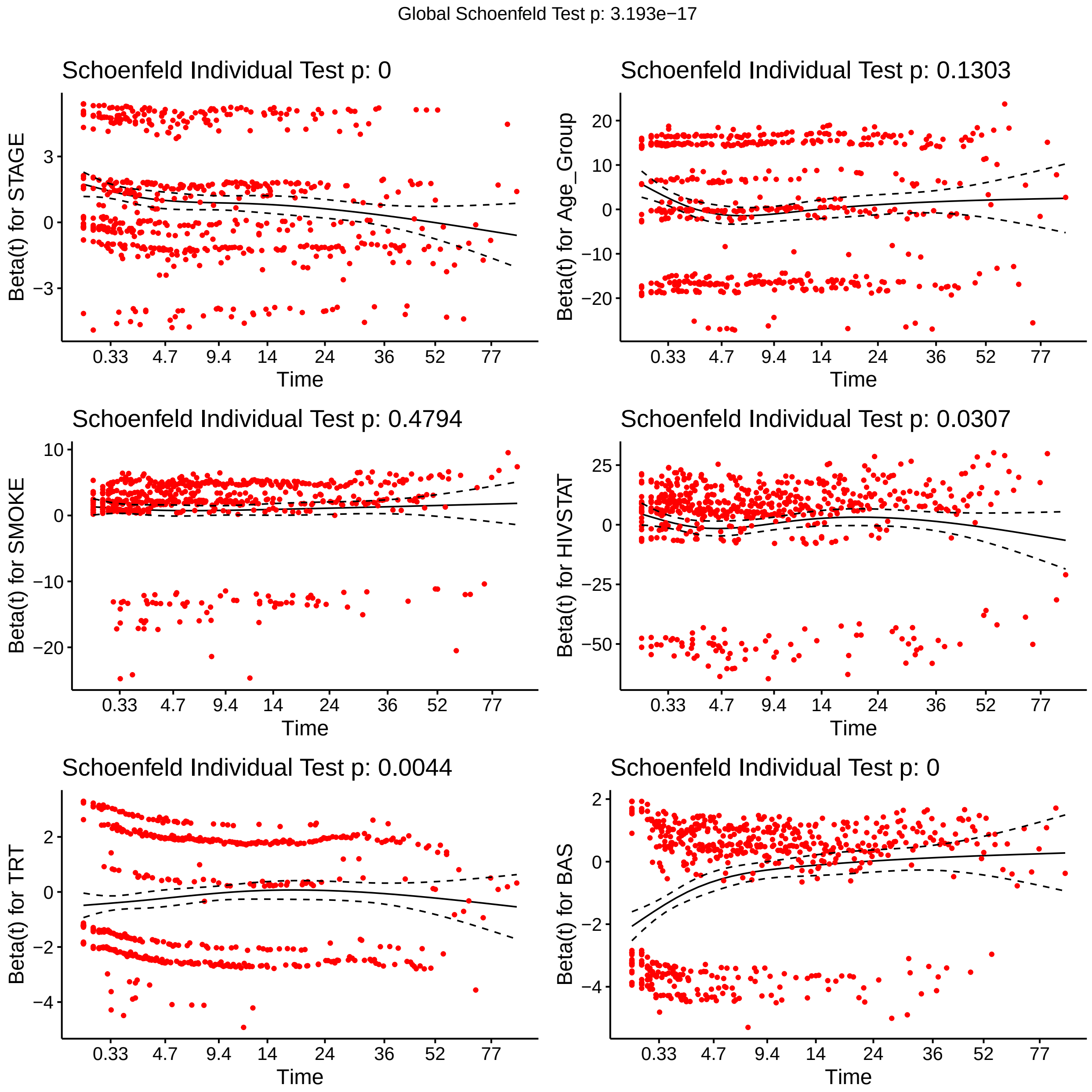

STAGE MODEL (STRAT: STAGE, BAS) - MULTIVARIATE COX PH

This was done to handle the violation of PH Assumptions from the previous model

```r
stagestratcox <- coxph(Surv(time2death, Censor) ~ Age_Group + SMOKE + HIVSTAT + TRT + strata(STAGE, BAS

# Summary of the model
summary(stagestratcox)
```

```
## Call:
## coxph(formula = Surv(time2death, Censor) ~ Age_Group + SMOKE +
##     HIVSTAT + TRT + strata(STAGE, BAS), data = cervical)
##
##   n= 2349, number of events= 603
##
##                       coef exp(coef) se(coef)      z Pr(>|z|)
## Age_Group>50     0.10941   1.11562  0.11062  0.989 0.322642
## Age_Group40-49  -0.03980   0.96099  0.11556 -0.344 0.730577
## SMOKEUnknown     0.52819   1.69586  0.13914  3.796 0.000147 ***
## SMOKEYes        -0.33252   0.71711  0.46786 -0.711 0.477256
## HIVSTATPositive  0.13242   1.14159  0.13254  0.999 0.317749
## HIVSTATUnknown   0.13097   1.13993  0.13852  0.945 0.344418
## TRTYes          -0.27511   0.75949  0.09149 -3.007 0.002638 **
## ---
## Signif. codes:  0 '***' 0.001 '**' 0.01 '*' 0.05 '.' 0.1 ' ' 1
##
##                 exp(coef) exp(-coef) lower .95 upper .95
## Age_Group>50       1.1156     0.8964    0.8982    1.3857
## Age_Group40-49     0.9610     1.0406    0.7662    1.2053
## SMOKEUnknown       1.6959     0.5897    1.2911    2.2275
## SMOKEYes           0.7171     1.3945    0.2866    1.7941
## HIVSTATPositive    1.1416     0.8760    0.8804    1.4802
## HIVSTATUnknown     1.1399     0.8772    0.8689    1.4955
## TRTYes             0.7595     1.3167    0.6348    0.9087
##
## Concordance= 0.583  (se = 0.016 )
## Likelihood ratio test= 42.47  on 7 df,   p=4e-07
## Wald test            = 38.67  on 7 df,   p=2e-06
## Score (logrank) test = 39.8  on 7 df,   p=1e-06
```

```r
# Plot the baseline survival function by STAGE
ggsurvplot(
  survfit(stagestratcox),
  data = cervical,
  ggtheme = theme_minimal(),
  legend.title = "STAGE",
  risk.table = TRUE
)
```

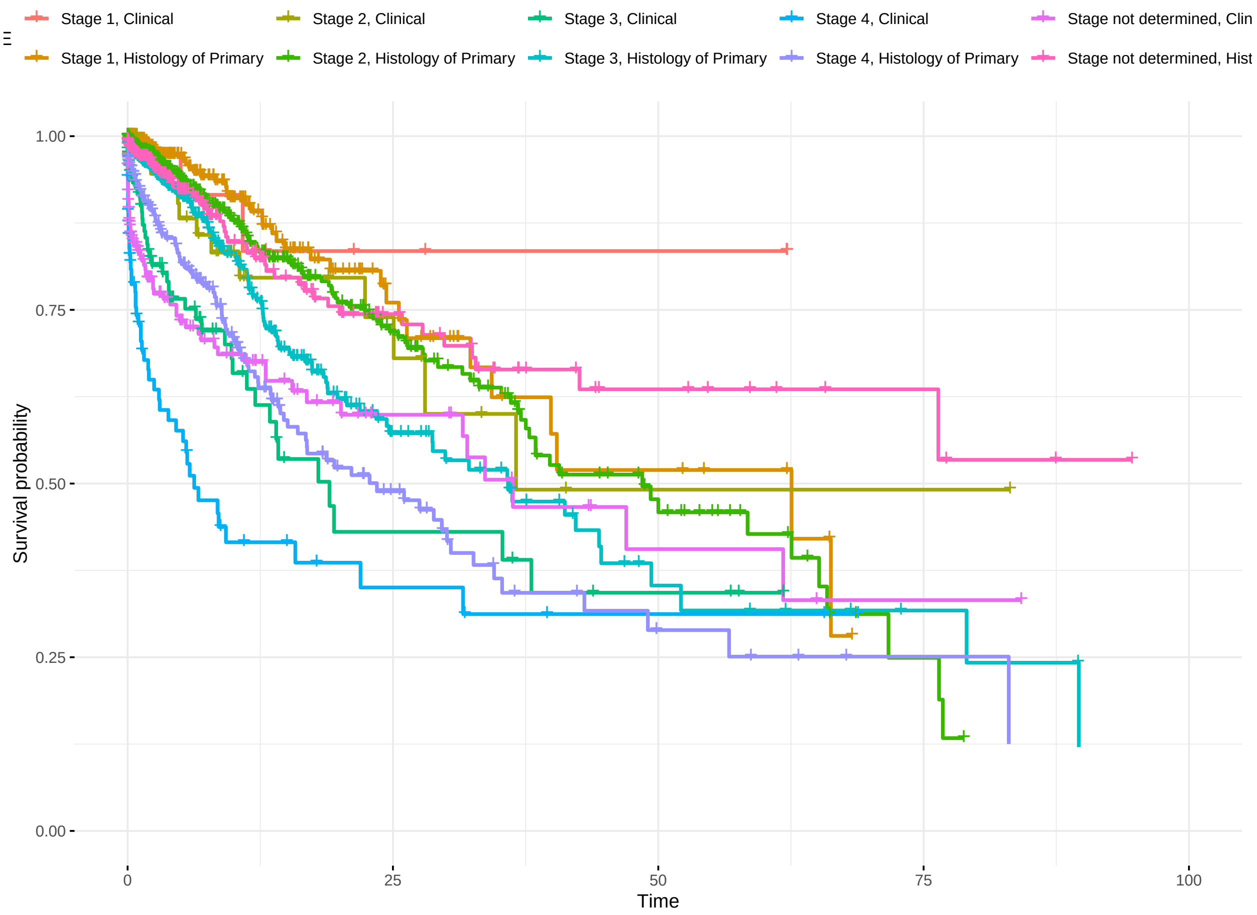

STAGE	0	25	50	75	100
Stage 1, Clinical	46	3	2	0	0
Stage 1, Histology of Primary	337	22	7	0	0
Stage 2, Clinical	83	7	1	1	0
Stage 2, Histology of Primary	444	65	21	3	0
Stage 3, Clinical	117	7	3	0	0
Stage 3, Histology of Primary	331	36	8	3	0
Stage 4, Clinical	78	5	2	0	0
Stage 4, Histology of Primary	191	26	5	1	0
Stage not determined, Clinical	241	13	3	1	0
Stage not determined, Histology of Primary	481	28	10	4	0

STAGE MODEL (STRAT: STAGE, BAS) - CHECKING ASSUMPTIONS

```r
# Test PH assumption
stagestrat.ph <- cox.zph(stagestratcox)
stagestrat.ph
```

```
##             chisq df    p
## Age_Group  1.22  2 0.54
## SMOKE      0.42  2 0.81
## HIVSTAT    1.34  2 0.51
## TRT        1.08  1 0.30
## GLOBAL     3.55  7 0.83
```

```r
# Graph PH
ggcoxzph(stagestrat.ph)
```

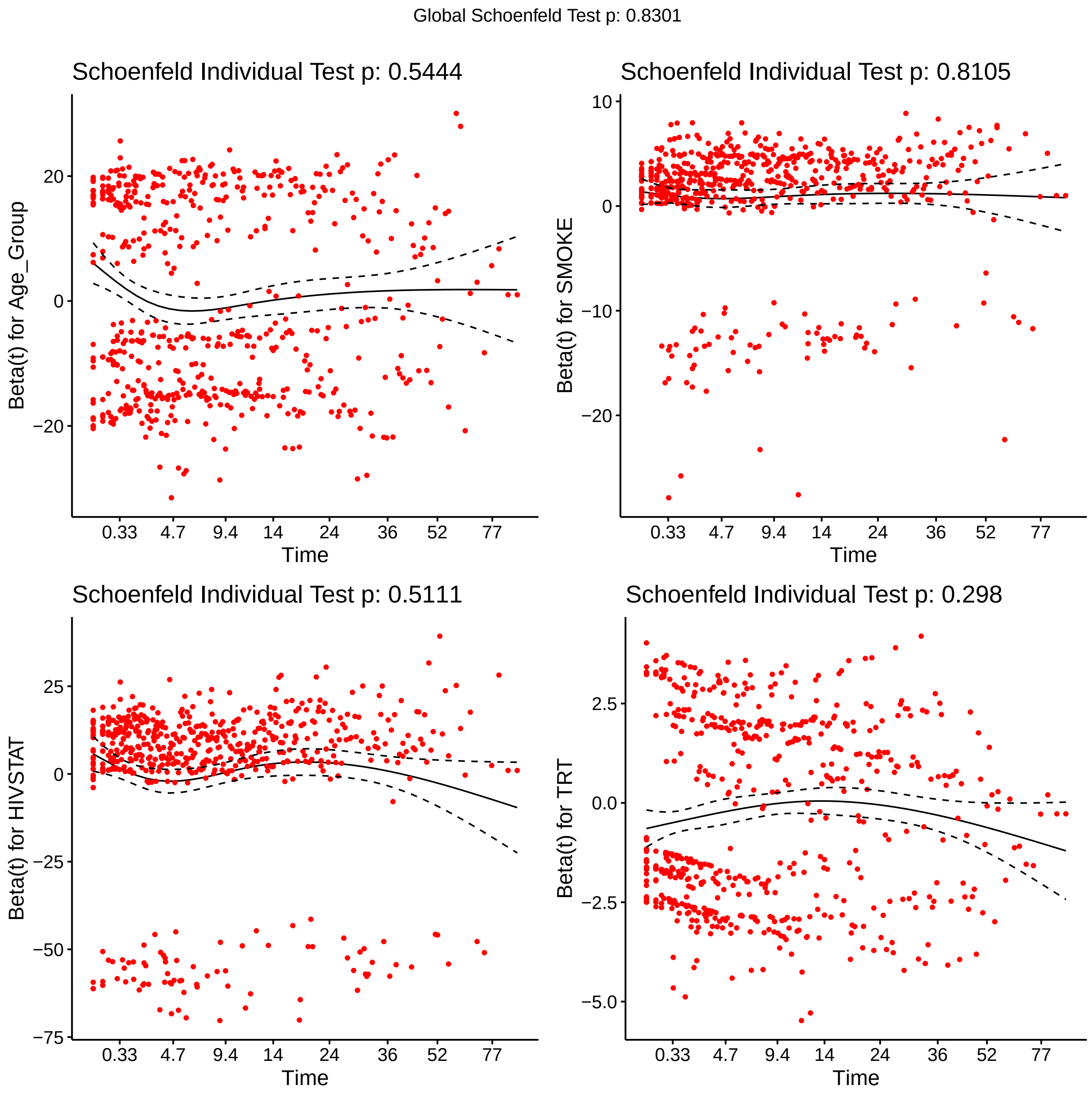

HIVxTRT - MULTIVARIATE COX PH

This model still stratifies by BAS and STAGE for consistency in testing our hypothesis as well as adressing the previous PH violation

```r
# Create Interaction Term
cervical$HIV_TRT <- interaction(cervical$HIVSTAT, cervical$TRT)
```

```r
# COX PH Model
HIVxTRT <- coxph(Surv(time2death, Censor) ~ Age_Group + SMOKE + HIVSTAT + TRT + HIV_TRT + strata(STAGE,

summary(HIVxTRT)
```

```
## Call:
## coxph(formula = Surv(time2death, Censor) ~ Age_Group + SMOKE +
##     HIVSTAT + TRT + HIV_TRT + strata(STAGE, BAS), data = cervical)
##
##   n= 2349, number of events= 603
##
##                         coef exp(coef) se(coef)      z Pr(>|z|)
## Age_Group>50         0.10718   1.11314  0.11067  0.968 0.332817
## Age_Group40-49      -0.04023   0.96057  0.11560 -0.348 0.727870
## SMOKEUnknown         0.51924   1.68075  0.13998  3.709 0.000208 ***
## SMOKEYes            -0.34202   0.71033  0.46869 -0.730 0.465553
## HIVSTATPositive      0.19415   1.21428  0.17227  1.127 0.259739
## HIVSTATUnknown       0.21580   1.24085  0.20038  1.077 0.281495
## TRTYes              -0.41390   0.66106  0.23576 -1.756 0.079155 .
## HIV_TRTPositive.No  -0.14761   0.86277  0.25816 -0.572 0.567471
## HIV_TRTUnknown.No   -0.17485   0.83958  0.27741 -0.630 0.528514
## HIV_TRTNegative.Yes       NA        NA  0.00000     NA       NA
## HIV_TRTPositive.Yes       NA        NA  0.00000     NA       NA
## HIV_TRTUnknown.Yes        NA        NA  0.00000     NA       NA
## ---
## Signif. codes:  0 '***' 0.001 '**' 0.01 '*' 0.05 '.' 0.1 ' ' 1
##
##                     exp(coef) exp(-coef) lower .95 upper .95
## Age_Group>50           1.1131     0.8984    0.8961     1.383
## Age_Group40-49         0.9606     1.0410    0.7658     1.205
## SMOKEUnknown           1.6808     0.5950    1.2775     2.211
## SMOKEYes               0.7103     1.4078    0.2835     1.780
## HIVSTATPositive        1.2143     0.8235    0.8663     1.702
## HIVSTATUnknown         1.2409     0.8059    0.8378     1.838
## TRTYes                 0.6611     1.5127    0.4165     1.049
## HIV_TRTPositive.No     0.8628     1.1591    0.5202     1.431
## HIV_TRTUnknown.No      0.8396     1.1911    0.4874     1.446
## HIV_TRTNegative.Yes        NA         NA        NA        NA
## HIV_TRTPositive.Yes        NA         NA        NA        NA
## HIV_TRTUnknown.Yes         NA         NA        NA        NA
##
## Concordance= 0.585  (se = 0.016 )
## Likelihood ratio test= 42.89  on 9 df,   p=2e-06
## Wald test            = 38.87  on 9 df,   p=1e-05
## Score (logrank) test = 40.04  on 9 df,   p=7e-06
```

```r
# Plot the baseline survival function
ggsurvplot(
  survfit(HIVxTRT),
  data = cervical,
  ggtheme = theme_minimal(),
  legend.title = "HIV × TRT Interaction",
  risk.table = TRUE
```

```
)
```

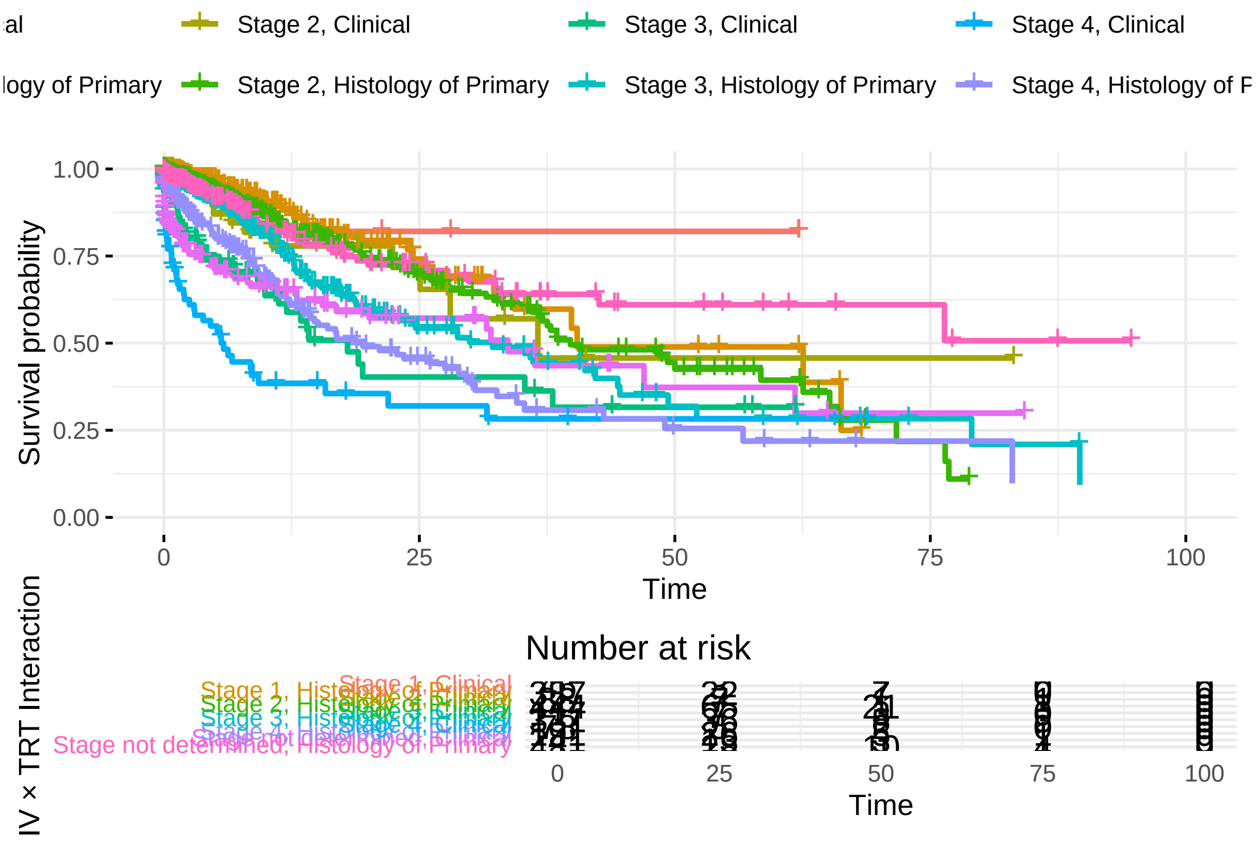

HIVxTRT - CHECKING ASSUMPTIONS

```
# Test PH assumption
HIVxTRT.ph <- cox.zph(HIVxTRT)
HIVxTRT.ph
```

```
##            chisq df    p
## Age_Group 1.106  2 0.58
## SMOKE     0.467  2 0.79
## HIVSTAT   1.586  2 0.45
## TRT       1.143  1 0.29
## HIV_TRT   4.316  2 0.12
## GLOBAL    6.035  9 0.74
```

```
# Graph PH
ggcoxzph(HIVxTRT.ph)
```

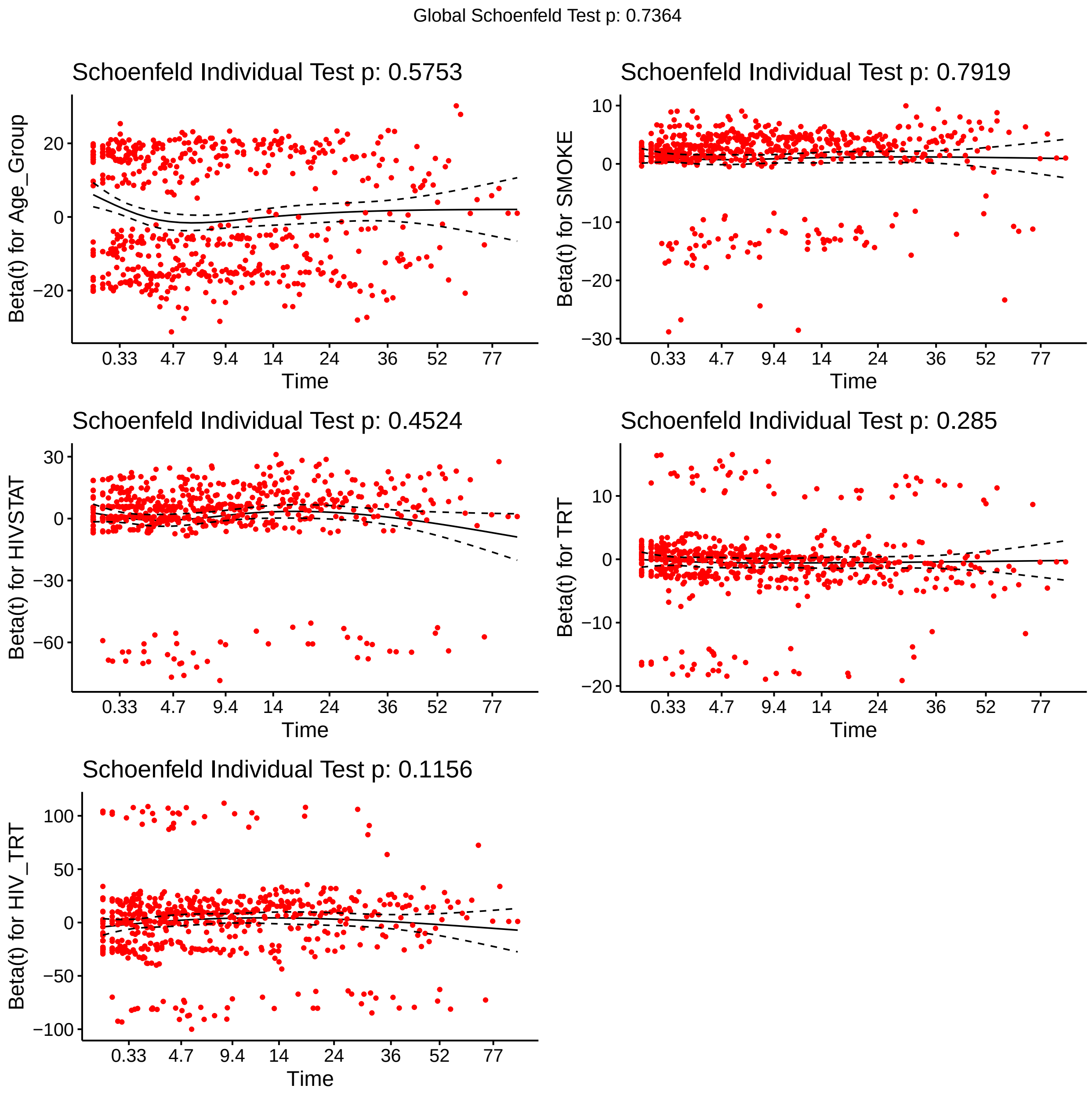

HIVxSTAGE - MULTIVARIATE COX PH

```
# Create intx term
cervical$HIV_STAGE <- interaction(cervical$HIVSTAT, cervical$STAGE)

# Cox PH Model
HIVxSTAGE <- coxph(Surv(time2death, Censor) ~ Age_Group + SMOKE + HIVSTAT + TRT + HIV_STAGE + strata(ST

summary(HIVxSTAGE)
```

```
## Call:
```

```
## coxph(formula = Surv(time2death, Censor) ~ Age_Group + SMOKE +
##     HIVSTAT + TRT + HIV_STAGE + strata(STAGE, BAS), data = cervical)
##
##   n= 2349, number of events= 603
##
##                                            coef exp(coef) se(coef)      z
## Age_Group>50                            0.09481   1.09945  0.11160  0.850
## Age_Group40-49                         -0.04157   0.95928  0.11572 -0.359
## SMOKEUnknown                            0.54385   1.72263  0.14056  3.869
## SMOKEYes                               -0.34250   0.71000  0.46920 -0.730
## HIVSTATPositive                         0.33599   1.39932  0.33551  1.001
## HIVSTATUnknown                          0.24791   1.28135  0.32813  0.756
## TRTYes                                 -0.24986   0.77891  0.09300 -2.687
## HIV_STAGEPositive.Stage 1              -1.09348   0.33505  0.53355 -2.049
## HIV_STAGEUnknown.Stage 1               -1.07242   0.34218  0.60568 -1.771
## HIV_STAGENegative.Stage 2               0.28085   1.32426  0.46369  0.606
## HIV_STAGEPositive.Stage 2              -0.04274   0.95816  0.29046 -0.147
## HIV_STAGEUnknown.Stage 2                     NA        NA  0.00000     NA
## HIV_STAGENegative.Stage 3               0.10803   1.11408  0.42323  0.255
## HIV_STAGEPositive.Stage 3               0.11187   1.11837  0.26008  0.430
## HIV_STAGEUnknown.Stage 3                     NA        NA  0.00000     NA
## HIV_STAGENegative.Stage 4              -0.14354   0.86629  0.41062 -0.350
## HIV_STAGEPositive.Stage 4              -0.41629   0.65949  0.26235 -1.587
## HIV_STAGEUnknown.Stage 4                     NA        NA  0.00000     NA
## HIV_STAGENegative.Stage not determined       NA        NA  0.00000     NA
## HIV_STAGEPositive.Stage not determined       NA        NA  0.00000     NA
## HIV_STAGEUnknown.Stage not determined        NA        NA  0.00000     NA
##                                        Pr(>|z|)
## Age_Group>50                           0.395580
## Age_Group40-49                         0.719410
## SMOKEUnknown                           0.000109 ***
## SMOKEYes                               0.465414
## HIVSTATPositive                        0.316620
## HIVSTATUnknown                         0.449926
## TRTYes                                 0.007218 **
## HIV_STAGEPositive.Stage 1              0.040418 *
## HIV_STAGEUnknown.Stage 1               0.076628 .
## HIV_STAGENegative.Stage 2              0.544717
## HIV_STAGEPositive.Stage 2              0.883022
## HIV_STAGEUnknown.Stage 2                     NA
## HIV_STAGENegative.Stage 3              0.798536
## HIV_STAGEPositive.Stage 3              0.667101
## HIV_STAGEUnknown.Stage 3                     NA
## HIV_STAGENegative.Stage 4              0.726661
## HIV_STAGEPositive.Stage 4              0.112562
## HIV_STAGEUnknown.Stage 4                     NA
## HIV_STAGENegative.Stage not determined       NA
## HIV_STAGEPositive.Stage not determined       NA
## HIV_STAGEUnknown.Stage not determined        NA
## ---
## Signif. codes:  0 '***' 0.001 '**' 0.01 '*' 0.05 '.' 0.1 ' ' 1
##
##                                        exp(coef) exp(-coef) lower .95 upper .95
## Age_Group>50                              1.0995     0.9095    0.8834    1.3683
```

```
## Age_Group40-49                          0.9593     1.0424    0.7646    1.2035
## SMOKEUnknown                            1.7226     0.5805    1.3078    2.2690
## SMOKEYes                                0.7100     1.4085    0.2831    1.7809
## HIVSTATPositive                         1.3993     0.7146    0.7250    2.7009
## HIVSTATUnknown                          1.2813     0.7804    0.6735    2.4376
## TRTYes                                  0.7789     1.2838    0.6491    0.9347
## HIV_STAGEPositive.Stage 1               0.3350     2.9847    0.1177    0.9534
## HIV_STAGEUnknown.Stage 1                0.3422     2.9224    0.1044    1.1215
## HIV_STAGENegative.Stage 2               1.3243     0.7551    0.5337    3.2860
## HIV_STAGEPositive.Stage 2               0.9582     1.0437    0.5422    1.6931
## HIV_STAGEUnknown.Stage 2                    NA         NA        NA        NA
## HIV_STAGENegative.Stage 3               1.1141     0.8976    0.4860    2.5537
## HIV_STAGEPositive.Stage 3               1.1184     0.8942    0.6717    1.8619
## HIV_STAGEUnknown.Stage 3                    NA         NA        NA        NA
## HIV_STAGENegative.Stage 4               0.8663     1.1544    0.3874    1.9372
## HIV_STAGEPositive.Stage 4               0.6595     1.5163    0.3944    1.1029
## HIV_STAGEUnknown.Stage 4                    NA         NA        NA        NA
## HIV_STAGENegative.Stage not determined      NA         NA        NA        NA
## HIV_STAGEPositive.Stage not determined      NA         NA        NA        NA
## HIV_STAGEUnknown.Stage not determined       NA         NA        NA        NA
##
## Concordance= 0.59  (se = 0.016 )
## Likelihood ratio test= 52.2  on 15 df,   p=5e-06
## Wald test            = 48.99  on 15 df,   p=2e-05
## Score (logrank) test = 50.6  on 15 df,   p=1e-05
```

```
# Plot the baseline survival function
ggsurvplot(
  survfit(HIVxSTAGE),
  data = cervical,
  ggtheme = theme_minimal(),
  legend.title = "HIV × STAGE Interaction",
  risk.table = TRUE
)
```

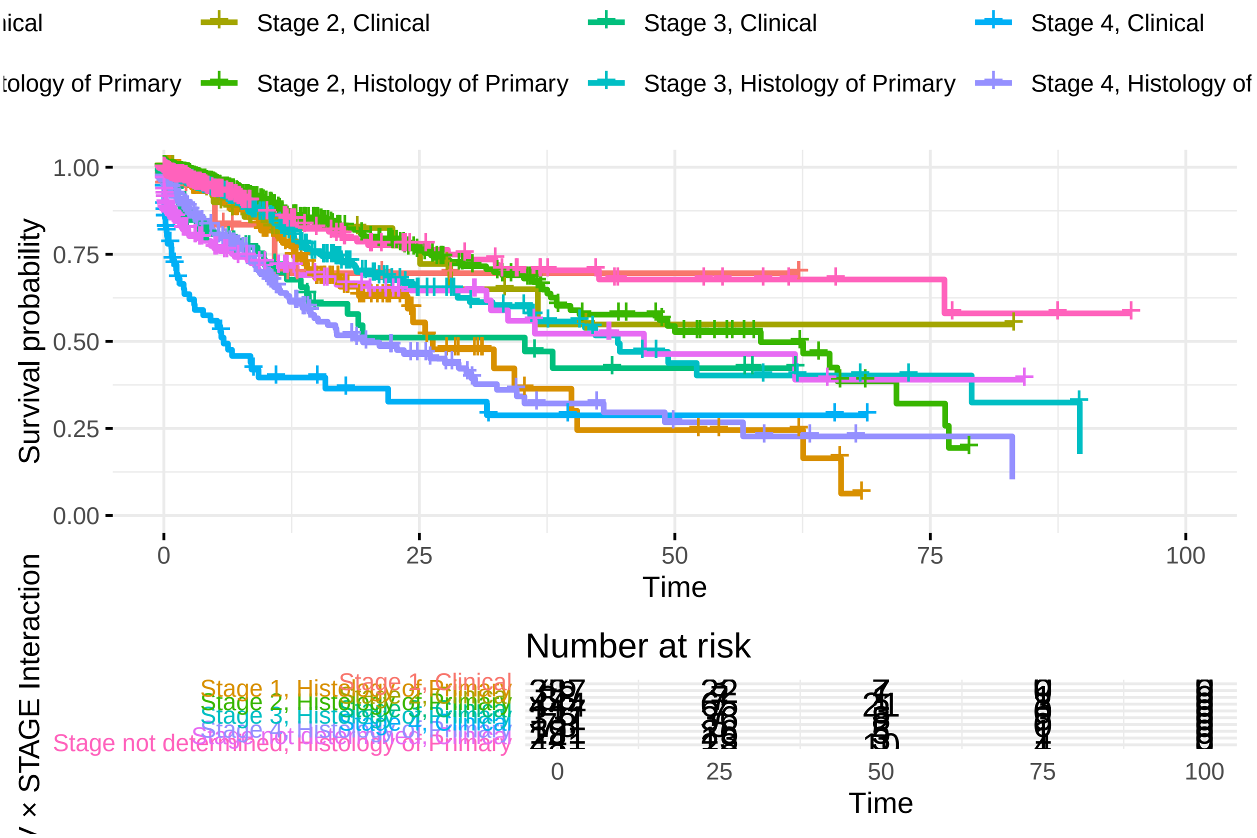

HIVxSTAGE - CHECKING ASSUMPTIONS

```
# Test PH assumption
HIVxSTAGE.ph <- cox.zph(HIVxSTAGE)
HIVxSTAGE.ph
```

```
##            chisq df     p
## Age_Group  1.388  2 0.500
## SMOKE      0.236  2 0.888
## HIVSTAT    0.958  2 0.619
## TRT        1.220  1 0.269
## HIV_STAGE 16.662  8 0.034
## GLOBAL    18.360 15 0.244
```

```
# Graph PH
ggcoxzph(HIVxSTAGE.ph)
```

```
## Warning: Removed 40 rows containing missing values or values outside the scale range
## (`geom_line()`).

## Warning: Removed 138 rows containing missing values or values outside the scale range
## (`geom_point()`).
```

```
## Warning: Removed 40 rows containing missing values or values outside the scale range
## (`geom_line()`).
## Removed 40 rows containing missing values or values outside the scale range
## (`geom_line()`).
```

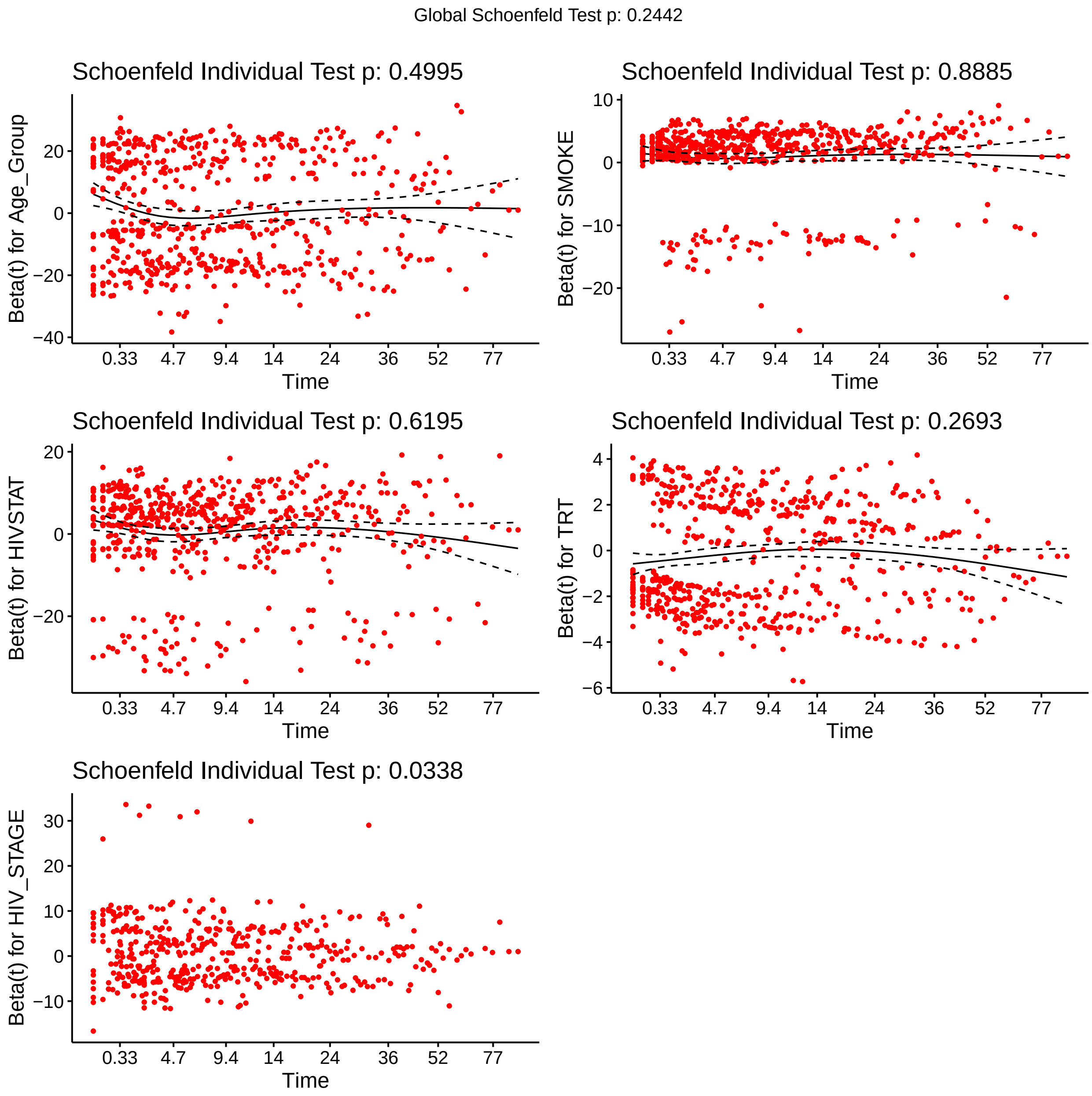

REMOVING SMOKING STATUS (NS) ##

NS - STAGE MODEL - MULTIVARIATE COX PH - NO SMOKE

```
## NS STAGE MODEL - MULTIVARIATE COX PH
nsstagecox <- coxph(Surv(time2death, Censor) ~ STAGE + Age_Group + HIVSTAT + TRT + BAS, data = cervical)
```

```r
# Summary of the model
summary(nsstagecox)
```

```
## Call:
## coxph(formula = Surv(time2death, Censor) ~ STAGE + Age_Group +
##     HIVSTAT + TRT + BAS, data = cervical)
##
##   n= 2349, number of events= 603
##
##                                coef exp(coef) se(coef)      z Pr(>|z|)
## STAGEStage 2                0.41496   1.51432  0.18321  2.265 0.023513 *
## STAGEStage 3                0.83536   2.30564  0.17797  4.694 2.68e-06 ***
## STAGEStage 4                1.31142   3.71145  0.17927  7.315 2.57e-13 ***
## STAGEStage not determined   0.61132   1.84287  0.18422  3.318 0.000905 ***
## Age_Group>50                0.09539   1.10008  0.10890  0.876 0.381093
## Age_Group40-49             -0.07925   0.92381  0.11471 -0.691 0.489625
## HIVSTATPositive             0.18593   1.20434  0.12998  1.430 0.152588
## HIVSTATUnknown              0.22512   1.25247  0.13536  1.663 0.096301 .
## TRTYes                     -0.28161   0.75457  0.08780 -3.208 0.001339 **
## BASHistology of Primary    -0.75300   0.47095  0.09124 -8.253  < 2e-16 ***
## ---
## Signif. codes:  0 '***' 0.001 '**' 0.01 '*' 0.05 '.' 0.1 ' ' 1
##
##                            exp(coef) exp(-coef) lower .95 upper .95
## STAGEStage 2                  1.5143     0.6604    1.0575    2.1685
## STAGEStage 3                  2.3056     0.4337    1.6267    3.2680
## STAGEStage 4                  3.7114     0.2694    2.6118    5.2740
## STAGEStage not determined     1.8429     0.5426    1.2844    2.6443
## Age_Group>50                  1.1001     0.9090    0.8886    1.3618
## Age_Group40-49                0.9238     1.0825    0.7378    1.1567
## HIVSTATPositive               1.2043     0.8303    0.9335    1.5538
## HIVSTATUnknown                1.2525     0.7984    0.9606    1.6330
## TRTYes                        0.7546     1.3253    0.6353    0.8963
## BASHistology of Primary       0.4710     2.1234    0.3938    0.5632
##
## Concordance= 0.723  (se = 0.012 )
## Likelihood ratio test= 192.6  on 10 df,   p=<2e-16
## Wald test            = 198.8  on 10 df,   p=<2e-16
## Score (logrank) test = 214.9  on 10 df,   p=<2e-16
```

```r
# Plot the baseline survival function by STAGE
ggsurvplot(
  survfit(nsstagecox),
  data = cervical,
  ggtheme = theme_minimal(),
  legend.title = "STAGE",
  risk.table = TRUE
)
```

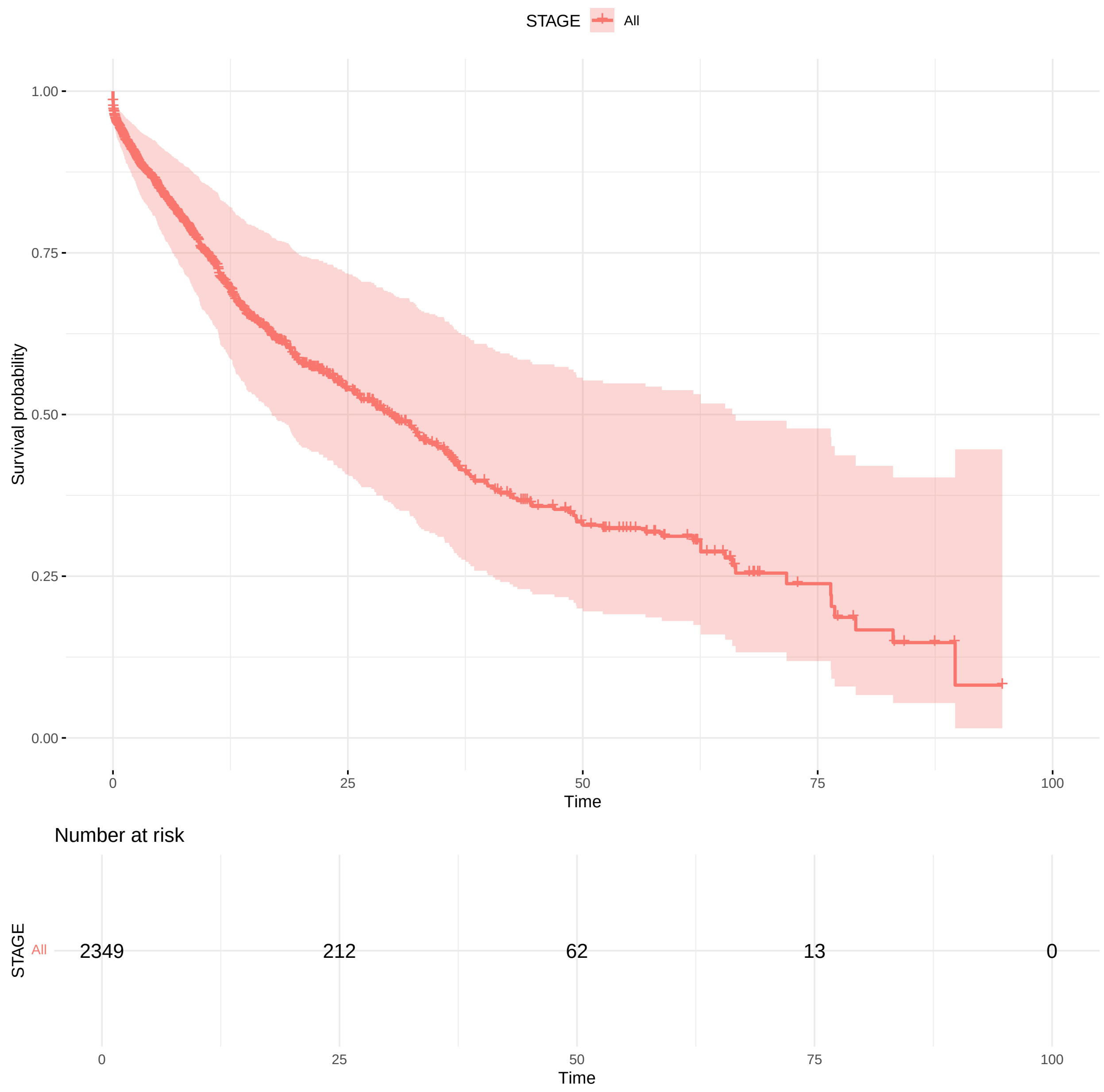

NS - STAGE MODEL - CHECKING ASSUMPTIONS

```
# Test PH assumption
nsstage.ph <- cox.zph(nsstagecox)
nsstage.ph
```

```
##              chisq df        p
## STAGE        57.34  4  1.1e-11
## Age_Group     3.37  2    0.185
## HIVSTAT       6.90  2    0.032
## TRT           6.58  1    0.010
## BAS          59.63  1  1.1e-14
```

```
## GLOBAL     101.90 10 < 2e-16
```

```r
# Graph PH
ggcoxzph(nsstage.ph)
```

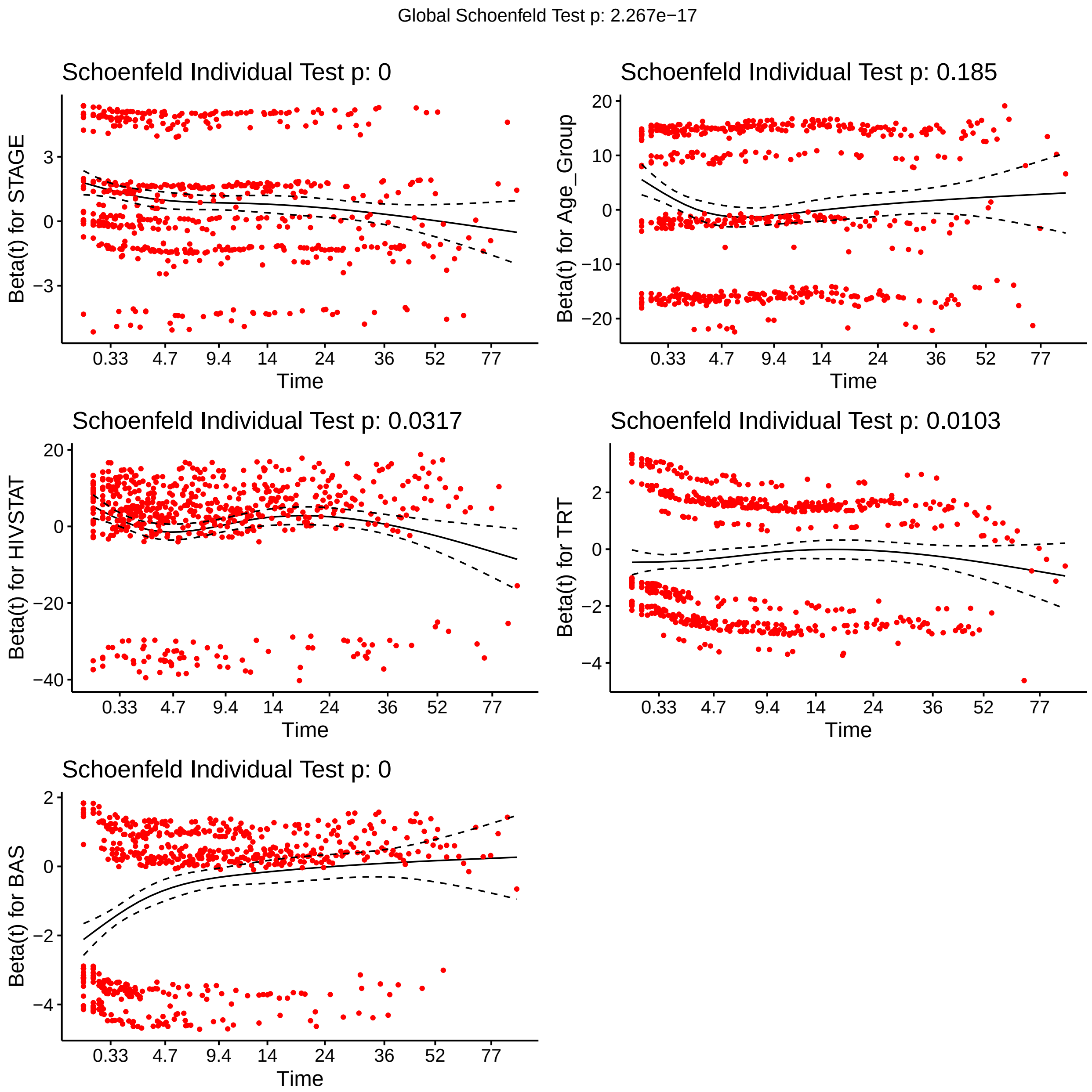

VIOLATION

NS - STAGE MODEL (STRAT: STAGE, BAS) - MULTIVARIATE COX PH

This was done to handle the violation of PH Assumptions from the previous model

```r
# Recode BAS variable
cervical$BAS <- ifelse(cervical$BAS == "Histology of Primary", "Histology", cervical$BAS)

cervical$BAS <- recode(cervical$BAS,
                       "Histology of Primary" = "Histology",
                       "Clinical" = "Clinical")  # Keep or shorten further if needed


cervical$STAGE[cervical$STAGE == "Stage not determined"] <- "Stage Unknown"


nsstagestratcox <- coxph(Surv(time2death, Censor) ~ Age_Group + HIVSTAT + TRT + strata(STAGE, BAS), data

# Summary of the model
summary(nsstagestratcox)
```

```
## Call:
## coxph(formula = Surv(time2death, Censor) ~ Age_Group + HIVSTAT +
##     TRT + strata(STAGE, BAS), data = cervical)
##
##   n= 2349, number of events= 603
##
##                    coef exp(coef) se(coef)      z Pr(>|z|)
## Age_Group>50     0.12086   1.12846  0.11035  1.095 0.273433
## Age_Group40-49  -0.04359   0.95734  0.11562 -0.377 0.706155
## HIVSTATPositive  0.18200   1.19961  0.13191  1.380 0.167657
## HIVSTATUnknown   0.23721   1.26771  0.13699  1.732 0.083339 .
## TRTYes          -0.35303   0.70255  0.09082 -3.887 0.000101 ***
## ---
## Signif. codes:  0 '***' 0.001 '**' 0.01 '*' 0.05 '.' 0.1 ' ' 1
##
##                 exp(coef) exp(-coef) lower .95 upper .95
## Age_Group>50       1.1285     0.8862    0.9090    1.4009
## Age_Group40-49     0.9573     1.0446    0.7632    1.2008
## HIVSTATPositive    1.1996     0.8336    0.9263    1.5535
## HIVSTATUnknown     1.2677     0.7888    0.9692    1.6581
## TRTYes             0.7026     1.4234    0.5880    0.8394
##
## Concordance= 0.57  (se = 0.016 )
## Likelihood ratio test= 23.21  on 5 df,   p=3e-04
## Wald test            = 23.25  on 5 df,   p=3e-04
## Score (logrank) test = 23.43  on 5 df,   p=3e-04
```

```r
p <- ggsurvplot(
  survfit(nsstagestratcox),
  data = cervical,
  ggtheme = theme_minimal(),
  legend.title = "Stage / Diagnosis",
  risk.table = TRUE,
  xlab = "Months",
  font.main = c(22, "bold"),          # Title font
  font.x = c(20),                     # X-axis label
```

```
  font.y = c(20),                      # Y-axis label
  font.tickslab = c(18),             # Axis tick labels
  font.legend = c(22)                # Legend text
)


# Save the plot as a PNG file
ggsave("survival_plot.png", plot = p$plot, width = 20, height = 12, dpi = 300)
```

SIGNIFICANT

- TRTYes: The treatment group (TRTYes) is statistically significant with a p-value of 9.73e-05 (***), indicating a strong effect on survival.

NOT SIGNIFICANT

- Age_Group39-50: p-value = 0.9650
- Age_Group50+: p-value = 0.2142
- HIVSTATPositive: p-value = 0.1638
- HIVSTATUnknown: p-value = 0.0809

MODEL PERFORMANCE

- Concordance Index: 0.57 (SE = 0.016), indicating moderate discrimination ability of the model.
- Likelihood Ratio Test = 23.07, df = 5, p = 3e-04 (significant).
- Wald Test = 23.14, df = 5, p = 3e-04 (significant).
- Score (Logrank) Test = 23.32, df = 5, p = 3e-04 (significant).
  These results show that the model fits the data well overall.

NS - COX PROPORTIONAL HAZARDS MODEL Survival Curve Clean

Survival curve separated by Clinical vs. Histology

```
library(dplyr)
library(ggplot2)
library(tidyr)
library(survival)
library(survminer)

# Your existing code to clean data and fit the model
cervical$BAS <- ifelse(cervical$BAS == "Histology of Primary", "Histology", cervical$BAS)
cervical$STAGE[cervical$STAGE == "Stage not determined"] <- "Stage Unknown"

nsstagestratcox <- coxph(Surv(time2death, Censor) ~ Age_Group + HIVSTAT + TRT + strata(STAGE, BAS), data
fit <- survfit(nsstagestratcox)
```

```r
fit_df <- surv_summary(fit, data = cervical)

fit_df <- fit_df %>%
  separate(strata, into = c("STAGE", "BAS"), sep = ", ") %>%
  mutate(
    STAGE = gsub("STAGE=", "", STAGE),
    BAS = gsub("BAS=", "", BAS)
  )

# Find the last time point for each curve to place labels
label_positions <- fit_df %>%
  group_by(STAGE, BAS) %>%
  filter(time == max(time)) %>%
  ungroup()

# Plot with direct labels instead of legend
ggplot(fit_df, aes(x = time, y = surv, color = STAGE)) +
  geom_step(size = 1.2) +
  facet_wrap(~BAS) +
  geom_text(data = label_positions,
            aes(label = STAGE),
            hjust = -0.1,    # Nudges label slightly to the right of the line end
            vjust = 0.5,
            size = 5,
            show.legend = FALSE) +
  labs(
    x = "Months of follow-up",
    y = "Survival probability",
    color = NULL
  ) +
  theme_minimal(base_size = 14) +
  theme(
    legend.position = "none",  # Remove legend since we have labels on the plot
    strip.text = element_text(size = 16, face = "bold"),
    axis.title = element_text(size = 16),
    axis.text = element_text(size = 14)
  ) +
  coord_cartesian(xlim = c(0, max(fit_df$time) * 1.1))  # Extend x-axis to fit labels outside plot area
```

```
## Warning: Using `size` aesthetic for lines was deprecated in ggplot2 3.4.0.
## i Please use `linewidth` instead.
## This warning is displayed once every 8 hours.
## Call `lifecycle::last_lifecycle_warnings()` to see where this warning was
## generated.
```

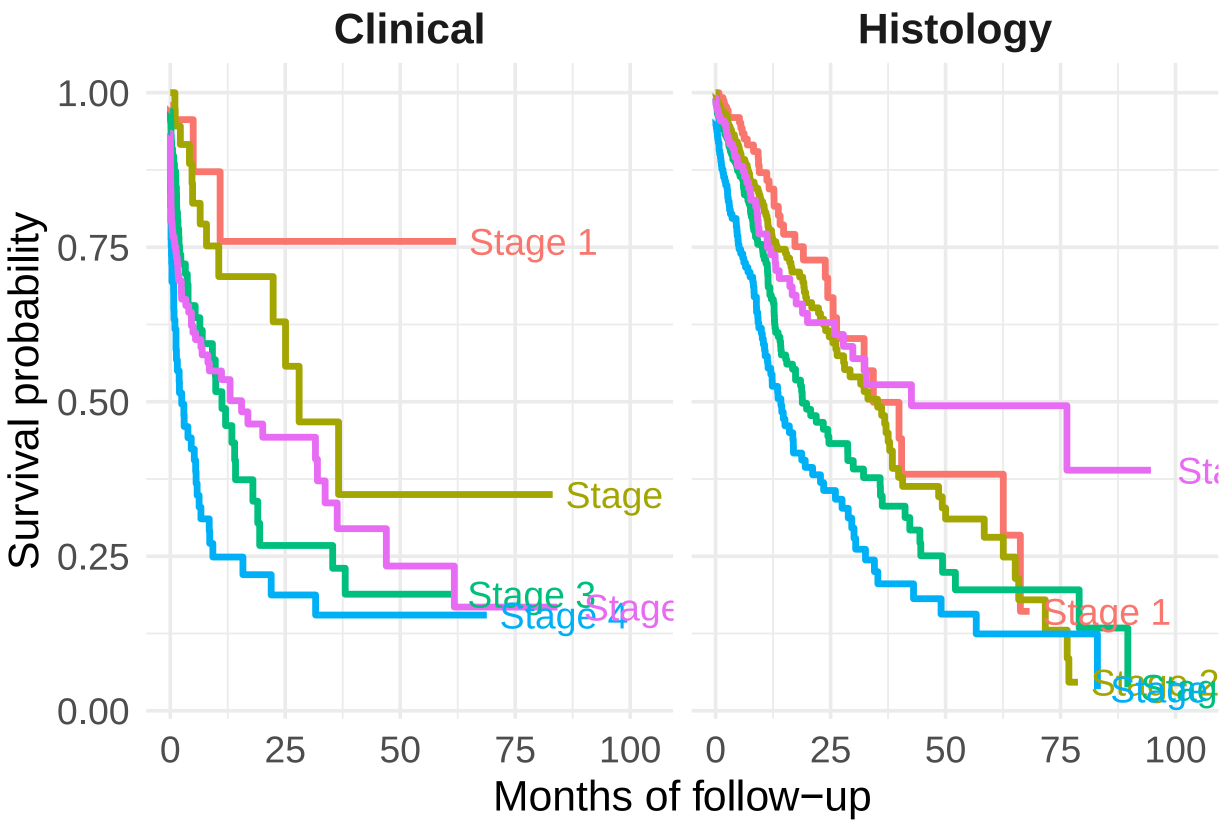


```r
# Lables on Side no overlap
library(ggrepel)

final_curve <- ggplot(fit_df, aes(x = time, y = surv, color = STAGE)) +
  geom_step(size = 1.2) +
  facet_wrap(~BAS) +
  geom_text_repel(data = label_positions,
                  aes(label = STAGE),
                  nudge_x = 5,        # shift labels right
                  direction = "y",
                  hjust = 0,
                  size = 7,
                  segment.color = NA, # no connecting lines
                  show.legend = FALSE) +
  labs(x = "Months of follow-up", y = "Survival probability", color = NULL) +
  theme_minimal(base_size = 16) +
  theme(
    legend.position = "none",
    strip.text = element_text(size = 20, face = "bold"),
    axis.title = element_text(size = 20),
    axis.text = element_text(size = 20)
  ) +
  coord_cartesian(xlim = c(0, max(fit_df$time) * 1.7))

ggsave("survival_plot_final.png", plot = final_curve, width = 20, height = 10, dpi = 300)
```

```
ggsave("survival_plot_final.tif",
       plot = final_curve,
       width = 20,
       height = 10,
       dpi = 300,
       device = "tiff")
```

NS - COX PROPORTIONAL HAZARDS MODEL FOREST PLOT

```
# Load libraries
library(survival)
library(forestplot)
```

```
## Loading required package: grid

## Loading required package: checkmate

## Loading required package: abind
```

```
library(survminer)
library(ragg)

model_summary <- summary(nsstagestratcox)

# Create data frame for forest plot
forest_data <- data.frame(
  Variable = rownames(model_summary$coefficients),
  HR = exp(model_summary$coefficients[, "coef"]),
  lower = model_summary$conf.int[, "lower .95"],
  upper = model_summary$conf.int[, "upper .95"],
  pval = model_summary$coefficients[, "Pr(>|z|)"]
)

# Replace variable names with clean, poster-friendly labels
forest_data$Variable <- c(
  "Age 39-50 vs <39",
  "Age >50 vs <39",
  "HIV Status: Positive",
  "HIV Status: Unknown",
  "Treatment Received: Yes"
)

# Create labels (you can customize this)
tabletext <- cbind(
  c("Variable", forest_data$Variable),
  c("Hazard Ratio", sprintf("%.2f", forest_data$HR)),
  c("95% CI", paste0(sprintf("%.2f", forest_data$lower), "-", sprintf("%.2f", forest_data$upper))),
  c("P-value", sprintf("%.3f", forest_data$pval))
)
```

```r
# Load ragg if not already
library(ragg)

# Save the plot to file
ragg::agg_png("/Users/citlallilopez/Documents/GRA/Eswatini - Cervical Cancer/forest_plot.png",
              width = 2400, height = 1200, res = 300)

forestplot(labeltext = tabletext,
           mean = c(NA, forest_data$HR),
           lower = c(NA, forest_data$lower),
           upper = c(NA, forest_data$upper),
           zero = 1,
           boxsize = 0.2,
           col = forestplot::fpColors(box = "royalblue", line = "darkblue", zero = "gray50"),
           xlab = "Hazard Ratio",
           title = "Forest Plot of Cox Model for Cervical Cancer-Specific Survival")

invisible(dev.off())
```

Creating bargraph to model survival distributions across stages based on diagnosis method

```r
# Load required libraries
library(survival)
library(survminer)
library(dplyr)
library(ggplot2)

# Extract survival estimates at 60 months from stratified Cox model
sf <- survfit(nsstagestratcox)
summary_60m <- summary(sf, times = 60)

# Create and clean survival data frame
surv_df <- data.frame(
  strata = summary_60m$strata,
  surv = summary_60m$surv,
  lower = summary_60m$lower,
  upper = summary_60m$upper
) %>%
  distinct(strata, .keep_all = TRUE)

# Manually assign clean labels (in desired order)
surv_df$Label <- c(
  "Stage 1 - Clinical", "Stage 1 - Histology",
  "Stage 2 - Clinical", "Stage 2 - Histology",
  "Stage 3 - Clinical", "Stage 3 - Histology",
  "Stage 4 - Clinical", "Stage 4 - Histology",
  "Stage Unknown - Clinical", "Stage Unknown - Histology"
)

# Reorder the factor levels so bars appear in correct order
label_order <- c(
  "Stage 1 - Clinical", "Stage 1 - Histology",
```

```r
  "Stage 2 - Clinical", "Stage 2 - Histology",
  "Stage 3 - Clinical", "Stage 3 - Histology",
  "Stage 4 - Clinical", "Stage 4 - Histology",
  "Stage Unknown - Clinical", "Stage Unknown - Histology"
)
surv_df$Label <- factor(surv_df$Label, levels = label_order)

# Manually assign Method (for fill color)
surv_df$Method <- ifelse(grepl("Histology", surv_df$Label), "Histology", "Clinical")

# Plot vertical bar chart
ggplot(surv_df, aes(x = Label, y = surv * 100, fill = Method)) +
  geom_bar(stat = "identity", width = 0.7) +
  geom_errorbar(aes(ymin = lower * 100, ymax = upper * 100), width = 0.2) +
  labs(
    title = "Estimated 5-Year Survival by Tumor Stage and Diagnosis Method",
    x = "",
    y = "5-Year Survival Probability (%)",
    fill = "Diagnosis Method"
  ) +
  scale_fill_manual(values = c("Clinical" = "#1f3b73", "Histology" = "#4B4B4B")) +
  theme_minimal(base_size = 14) +
  theme(
    axis.text.x = element_text(angle = 45, hjust = 1, size = 12),
    legend.position = "top",
    plot.title = element_text(hjust = 0.5)
  ) +
  ylim(0, 100)
```

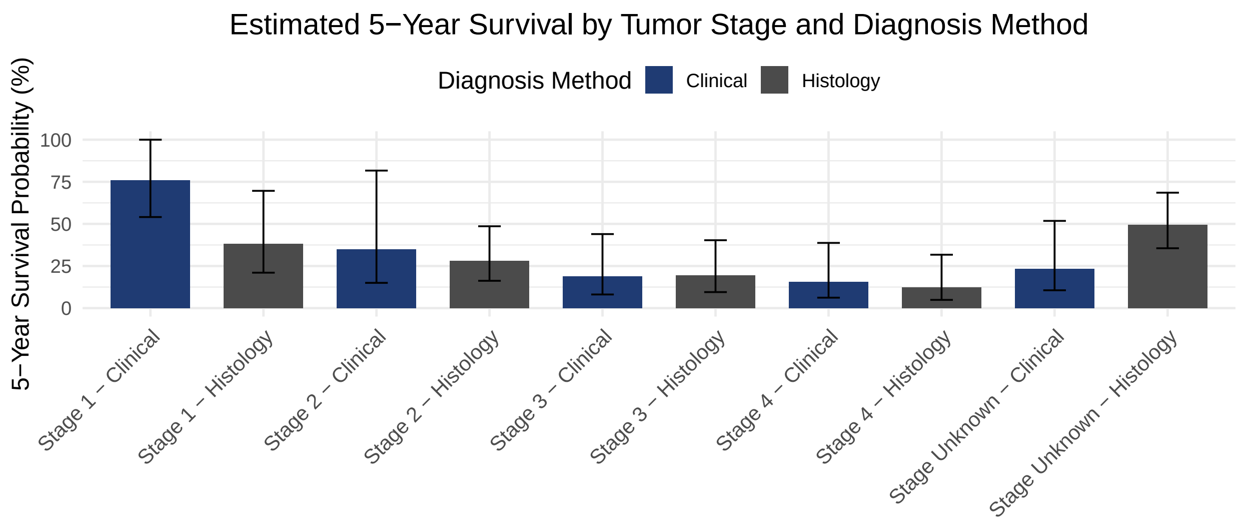


```r
ggsave("bargraph_survival.png", width = 10, height = 6, dpi = 300)
```

HYPOTHESIS CONFIRMED

- The significance of TRTYes confirms that treatment has a statistically significant impact on survival. However, other factors such as age group and HIV status were not significant predictors in this analysis.

NS - STAGE MODEL (STRAT: STAGE, BAS) - CHECKING ASSUMPTIONS

```
# Test PH assumption
nsstagestrat.ph <- cox.zph(nsstagestratcox)
nsstagestrat.ph
```

```
##            chisq df    p
## Age_Group 0.990  2 0.61
## HIVSTAT   1.213  2 0.55
## TRT       0.332  1 0.56
## GLOBAL    2.341  5 0.80
```

```
# Graph PH
ggcoxzph(stagestrat.ph)
```

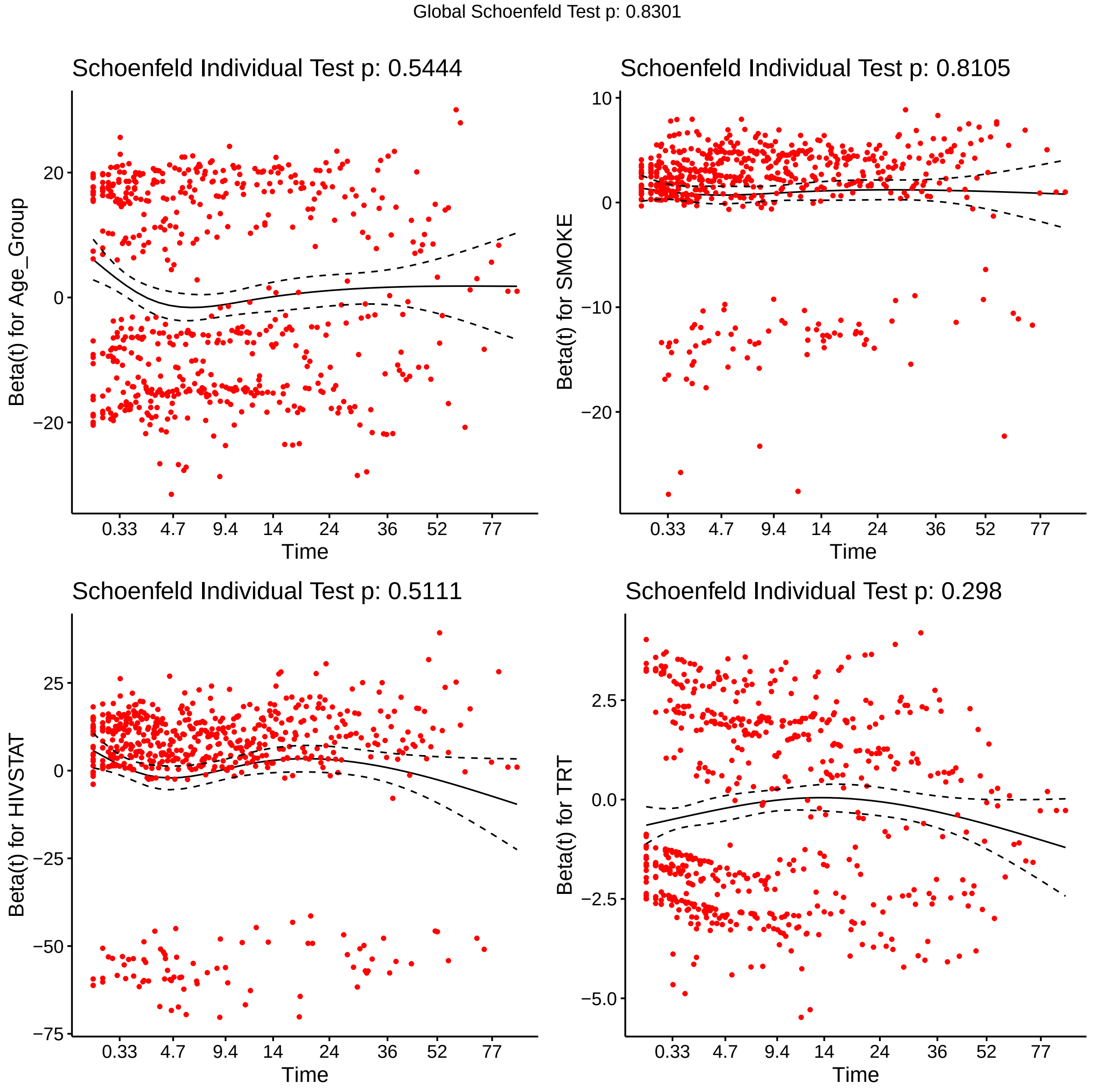

No violation of PH for individual covariates or the overall model (global test).

Stratification addressed the violation of the PH assumption as it controls for the time-dependent effect of Stage and Basis of diagnosis. Stratifying also assisted in addressing the mild PH violation for HIVSTAT and TRT.

NS - HIVxTRT - MULTIVARIATE COX PH

This model still stratifies by BAS and STAGE for consistency in testing our hypothesis as well as addressing the previous PH violation

```
# Create Interaction Term
cervical$HIV_TRT <- interaction(cervical$HIVSTAT, cervical$TRT)
```

```r
# COX PH Model
nsHIVxTRT <- coxph(Surv(time2death, Censor) ~ Age_Group + HIVSTAT + TRT + HIV_TRT + strata(STAGE, BAS),

summary(nsHIVxTRT)
```

```
## Call:
## coxph(formula = Surv(time2death, Censor) ~ Age_Group + HIVSTAT +
##     TRT + HIV_TRT + strata(STAGE, BAS), data = cervical)
##
##   n= 2349, number of events= 603
##
##                          coef exp(coef) se(coef)      z Pr(>|z|)
## Age_Group>50          0.11867   1.12600  0.11036  1.075   0.2822
## Age_Group40-49       -0.04368   0.95726  0.11561 -0.378   0.7055
## HIVSTATPositive       0.27068   1.31086  0.17118  1.581   0.1138
## HIVSTATUnknown        0.37804   1.45942  0.19638  1.925   0.0542 .
## TRTYes               -0.56786   0.56673  0.23304 -2.437   0.0148 *
## HIV_TRTPositive.No   -0.21796   0.80416  0.25706 -0.848   0.3965
## HIV_TRTUnknown.No    -0.29044   0.74794  0.27510 -1.056   0.2911
## HIV_TRTNegative.Yes        NA        NA  0.00000     NA       NA
## HIV_TRTPositive.Yes        NA        NA  0.00000     NA       NA
## HIV_TRTUnknown.Yes         NA        NA  0.00000     NA       NA
## ---
## Signif. codes:  0 '***' 0.001 '**' 0.01 '*' 0.05 '.' 0.1 ' ' 1
##
##                      exp(coef) exp(-coef) lower .95 upper .95
## Age_Group>50            1.1260     0.8881    0.9070    1.3979
## Age_Group40-49          0.9573     1.0447    0.7632    1.2007
## HIVSTATPositive         1.3109     0.7629    0.9372    1.8334
## HIVSTATUnknown          1.4594     0.6852    0.9932    2.1446
## TRTYes                  0.5667     1.7645    0.3589    0.8948
## HIV_TRTPositive.No      0.8042     1.2435    0.4859    1.3309
## HIV_TRTUnknown.No       0.7479     1.3370    0.4362    1.2824
## HIV_TRTNegative.Yes         NA         NA        NA        NA
## HIV_TRTPositive.Yes         NA         NA        NA        NA
## HIV_TRTUnknown.Yes          NA         NA        NA        NA
##
## Concordance= 0.572  (se = 0.016 )
## Likelihood ratio test= 24.32  on 7 df,   p=0.001
## Wald test            = 23.88  on 7 df,   p=0.001
## Score (logrank) test = 24.14  on 7 df,   p=0.001
```

```r
# Plot the baseline survival function
ggsurvplot(
  survfit(nsHIVxTRT),
  data = cervical,
  ggtheme = theme_minimal(),
  legend.title = "HIV × TRT Interaction",
  risk.table = TRUE
)
```

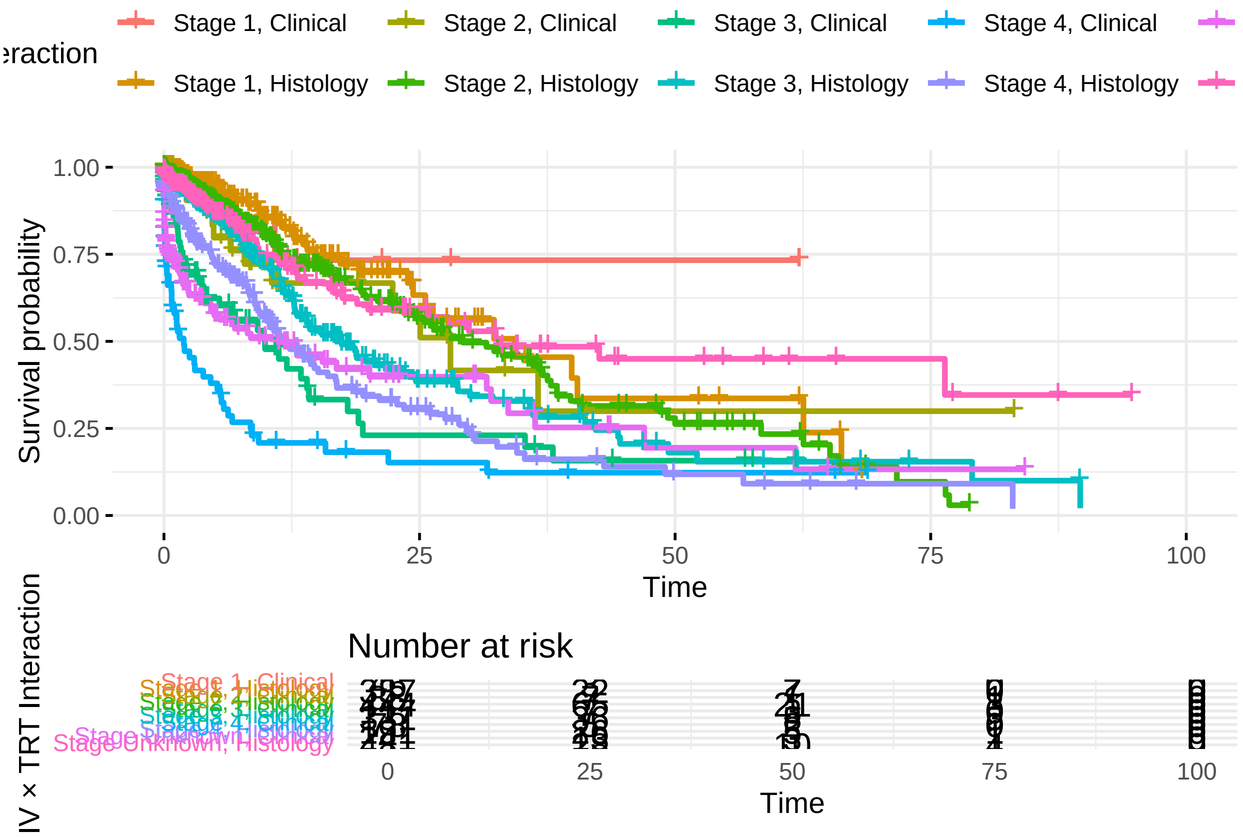

SIGNIFICANT

- TRTYes: Treatment group (TRTYes) is statistically significant with a p-value of 0.0146 (*), indicating a notable effect on survival.

NOT SIGNIFICANT

- Age_Group39-50: p-value = 0.9662
- Age_Group50+: p-value = 0.2207
- HIVSTATPositive: p-value = 0.1112
- HIVSTATUnknown: p-value = 0.0532 (marginally significant but not below 0.05).
- HIV_TRTPossible.No: p-value = 0.3945
- HIV_TRTUnknown.No: p-value = 0.2915

  Additional interactions (HIV_TRTNegative.Yes, HIV_TRTPositive.Yes, HIV_TRTUnknown.Yes) lack values, suggesting no effect or unestimable parameters.

MODEL PERFORMANCE

- Concordance Index: 0.572 (SE = 0.016), indicating moderate discrimination ability of the model.
- Likelihood Ratio Test = 24.18, df = 7, p = 0.001 (significant).

- Wald Test = 23.76, df = 7, p = 0.001 (significant).
- Score (Logrank) Test = 24.02, df = 7, p = 0.001 (significant).

These metrics show that the overall model fits well.

HYPOTHESIS CONFIRMED

- The treatment variable (TRTYes) remains statistically significant, confirming its effect on survival. However, other variables and interaction terms (e.g., age groups, HIV status) were not significant in this analysis. This indicates that treatment has the primary influence on the outcome among the factors analyzed.

NS - HIVxTRT - CHECKING ASSUMPTIONS

```r
# Test PH assumption
nsHIVxTRT.ph <- cox.zph(nsHIVxTRT)
nsHIVxTRT.ph
```

```
##             chisq df    p
## Age_Group 0.813  2 0.67
## HIVSTAT   1.613  2 0.45
## TRT       0.403  1 0.53
## HIV_TRT   3.703  2 0.16
## GLOBAL    4.659  7 0.70
```

```r
# Graph PH
ggcoxzph(nsHIVxTRT.ph)
```

Global Schoenfeld Test p: 0.7015

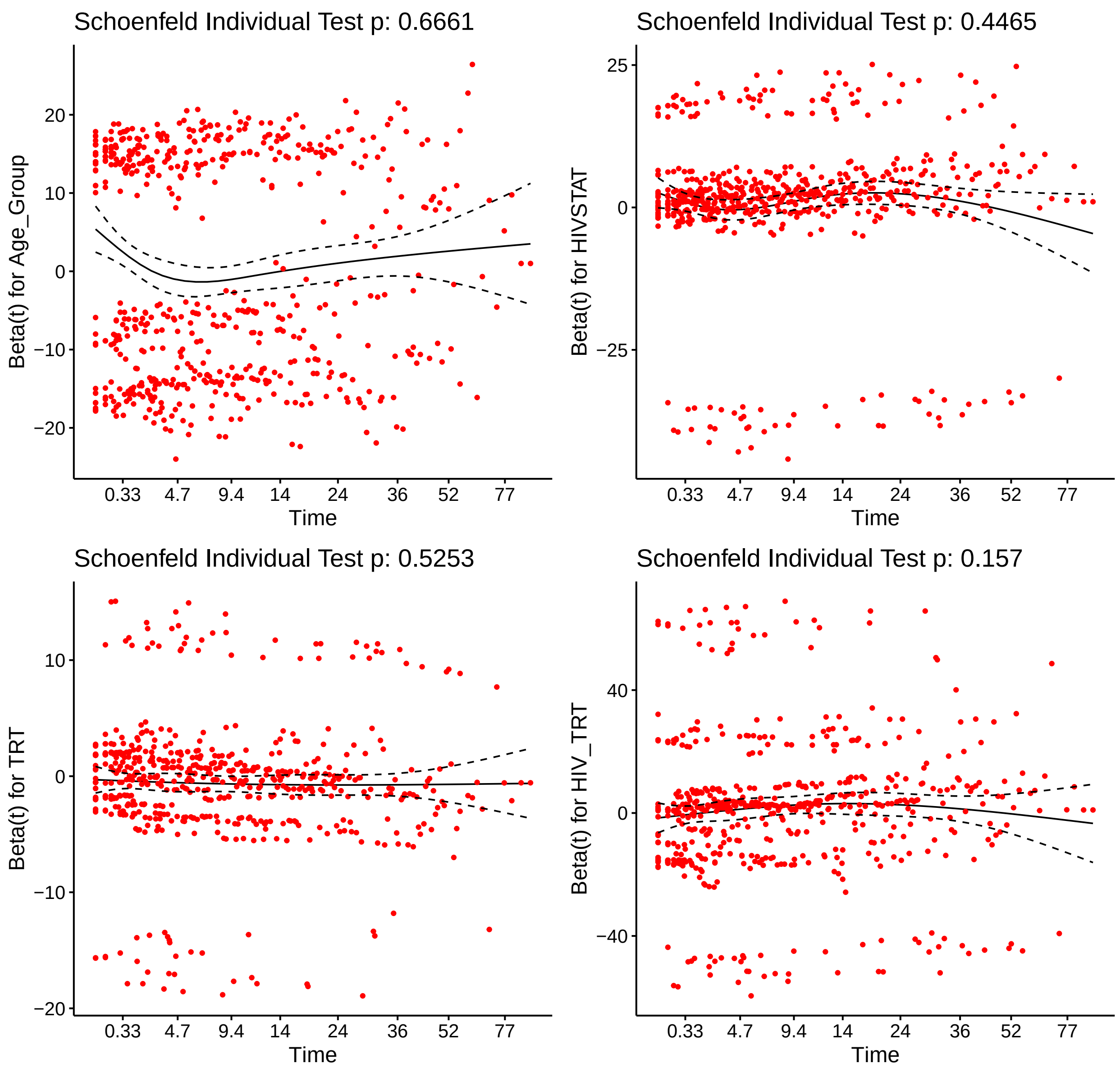

NO PH VIOLATIONS

NS - HIVxSTAGE - MULTIVARIATE COX PH

```
# Create intx term
cervical$HIV_STAGE <- interaction(cervical$HIVSTAT, cervical$STAGE)

# Cox PH Model
nsHIVxSTAGE <- coxph(Surv(time2death, Censor) ~ Age_Group  + HIVSTAT + TRT + HIV_STAGE + strata(STAGE, 

summary(nsHIVxSTAGE)
```

```
## Call:
## coxph(formula = Surv(time2death, Censor) ~ Age_Group + HIVSTAT +
##     TRT + HIV_STAGE + strata(STAGE, BAS), data = cervical)
##
##   n= 2349, number of events= 603
##
##                                     coef exp(coef) se(coef)      z Pr(>|z|)
## Age_Group>50                     0.10711   1.11306  0.11129  0.962  0.33581
## Age_Group40-49                  -0.04639   0.95467  0.11586 -0.400  0.68887
## HIVSTATPositive                  0.32986   1.39078  0.33504  0.985  0.32485
## HIVSTATUnknown                   0.27778   1.32020  0.32770  0.848  0.39663
## TRTYes                          -0.32755   0.72069  0.09235 -3.547  0.00039 ***
## HIV_STAGEPositive.Stage 1       -0.97172   0.37843  0.53339 -1.822  0.06849 .
## HIV_STAGEUnknown.Stage 1        -0.95557   0.38459  0.60684 -1.575  0.11533
## HIV_STAGENegative.Stage 2        0.06359   1.06566  0.46142  0.138  0.89039
## HIV_STAGEPositive.Stage 2       -0.10295   0.90217  0.29035 -0.355  0.72291
## HIV_STAGEUnknown.Stage 2              NA        NA  0.00000     NA       NA
## HIV_STAGENegative.Stage 3        0.02318   1.02345  0.42212  0.055  0.95621
## HIV_STAGEPositive.Stage 3        0.08083   1.08418  0.25963  0.311  0.75556
## HIV_STAGEUnknown.Stage 3              NA        NA  0.00000     NA       NA
## HIV_STAGENegative.Stage 4       -0.18960   0.82729  0.41048 -0.462  0.64415
## HIV_STAGEPositive.Stage 4       -0.44418   0.64135  0.26225 -1.694  0.09032 .
## HIV_STAGEUnknown.Stage 4              NA        NA  0.00000     NA       NA
## HIV_STAGENegative.Stage Unknown       NA        NA  0.00000     NA       NA
## HIV_STAGEPositive.Stage Unknown       NA        NA  0.00000     NA       NA
## HIV_STAGEUnknown.Stage Unknown        NA        NA  0.00000     NA       NA
## ---
## Signif. codes:  0 '***' 0.001 '**' 0.01 '*' 0.05 '.' 0.1 ' ' 1
##
##                                 exp(coef) exp(-coef) lower .95 upper .95
## Age_Group>50                       1.1131     0.8984    0.8949    1.3843
## Age_Group40-49                     0.9547     1.0475    0.7607    1.1980
## HIVSTATPositive                    1.3908     0.7190    0.7212    2.6819
## HIVSTATUnknown                     1.3202     0.7575    0.6945    2.5095
## TRTYes                             0.7207     1.3876    0.6014    0.8637
## HIV_STAGEPositive.Stage 1          0.3784     2.6425    0.1330    1.0765
## HIV_STAGEUnknown.Stage 1           0.3846     2.6001    0.1171    1.2634
## HIV_STAGENegative.Stage 2          1.0657     0.9384    0.4314    2.6326
## HIV_STAGEPositive.Stage 2          0.9022     1.1084    0.5107    1.5938
## HIV_STAGEUnknown.Stage 2               NA         NA        NA        NA
## HIV_STAGENegative.Stage 3          1.0235     0.9771    0.4475    2.3409
## HIV_STAGEPositive.Stage 3          1.0842     0.9224    0.6518    1.8034
## HIV_STAGEUnknown.Stage 3               NA         NA        NA        NA
## HIV_STAGENegative.Stage 4          0.8273     1.2088    0.3700    1.8495
## HIV_STAGEPositive.Stage 4          0.6413     1.5592    0.3836    1.0723
## HIV_STAGEUnknown.Stage 4               NA         NA        NA        NA
## HIV_STAGENegative.Stage Unknown        NA         NA        NA        NA
## HIV_STAGEPositive.Stage Unknown        NA         NA        NA        NA
## HIV_STAGEUnknown.Stage Unknown         NA         NA        NA        NA
##
## Concordance= 0.579  (se = 0.016 )
## Likelihood ratio test= 32.08  on 13 df,   p=0.002
## Wald test            = 32.77  on 13 df,   p=0.002
## Score (logrank) test = 33.38  on 13 df,   p=0.001
```

```r
# Plot the baseline survival function
ggsurvplot(
  survfit(nsHIVxSTAGE),
  data = cervical,
  ggtheme = theme_minimal(),
  legend.title = "HIV × STAGE Interaction",
  risk.table = TRUE
)
```

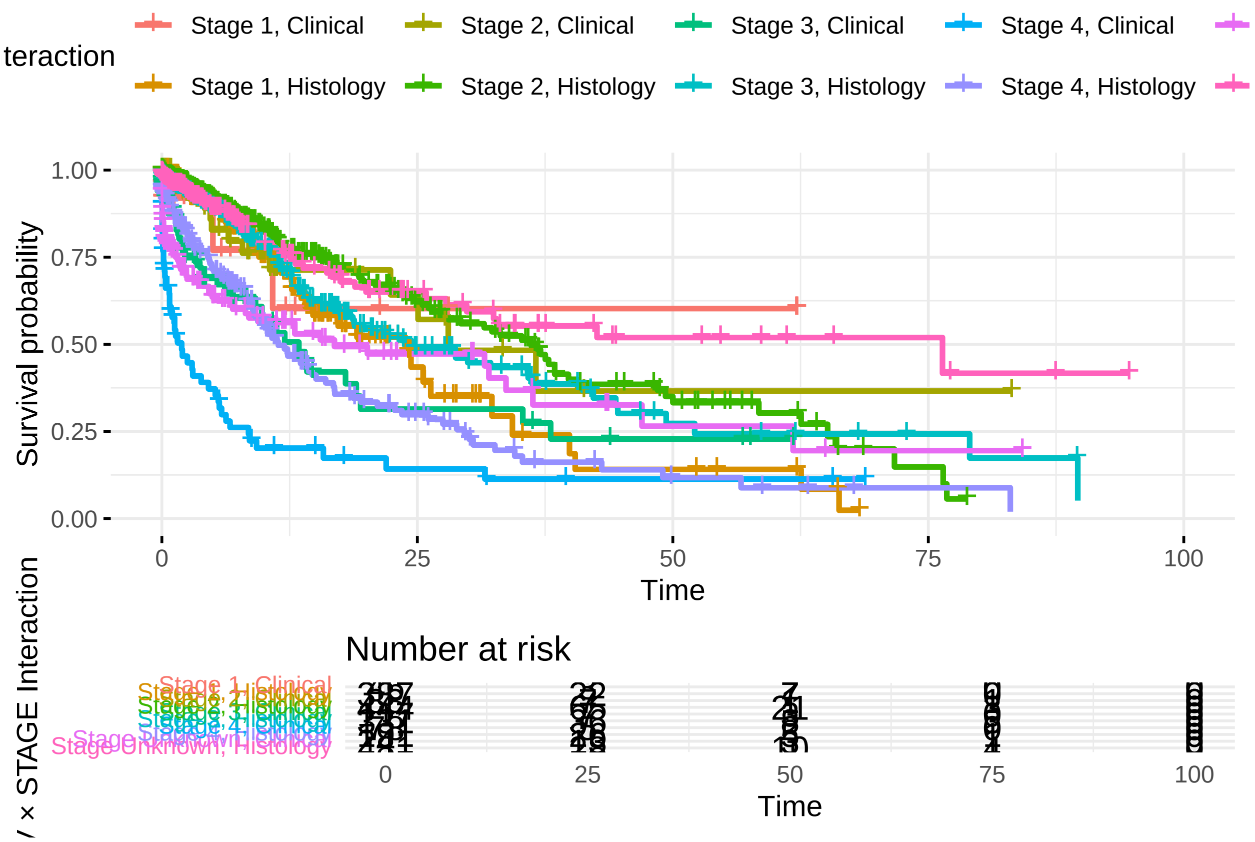

SIGNIFICANT

- TRTYes: Treatment (TRTYes) remains statistically significant with a p-value of 0.000375 (***), indicating a strong effect on survival.

NOT SIGNIFICANT

- Age_Group39-50: p-value = 0.950362
- Age_Group50+: p-value = 0.263948
- HIVSTATPositive: p-value = 0.321475
- HIVSTATUnknown: p-value = 0.395475
- Other interaction terms (e.g., HIV_STAGE combinations) are generally not significant with p-values above 0.05 or unestimable.

MODEL PERFORMANCE

- Concordance Index: 0.578 (SE = 0.016), indicating moderate discrimination ability of the model.
- Likelihood Ratio Test = 31.93, df = 13, p = 0.002 (significant).
- Wald Test = 32.65, df = 13, p = 0.002 (significant).
- Score (Logrank) Test= 33.25, df = 13, p = 0.002 (significant).

These results confirm the model fits the data well, though the concordance index suggests moderate predictive performance.

HYPOTHESIS CONFIRMED
The treatment variable (TRTYes) remains the most significant factor, demonstrating a clear and consistent impact on survival. However, age group, HIV status, and stage-specific interactions do not show strong or consistent significance, indicating limited additional predictive power from these variables.

HIVxSTAGE - CHECKING ASSUMPTIONS

```
# Test PH assumption
nsHIVxSTAGE.ph <- cox.zph(nsHIVxSTAGE)
nsHIVxSTAGE.ph
```

```
##            chisq df     p
## Age_Group  1.082  2 0.582
## HIVSTAT    0.946  2 0.623
## TRT        0.410  1 0.522
## HIV_STAGE 17.169  8 0.028
## GLOBAL    18.057 13 0.155
```

```
# Graph PH
ggcoxzph(nsHIVxSTAGE.ph)
```

```
## Warning: Removed 40 rows containing missing values or values outside the scale range
## (‘geom_line()‘).
```

```
## Warning: Removed 138 rows containing missing values or values outside the scale range
## (‘geom_point()‘).
```

```
## Warning: Removed 40 rows containing missing values or values outside the scale range
## (‘geom_line()‘).
## Removed 40 rows containing missing values or values outside the scale range
## (‘geom_line()‘).
```

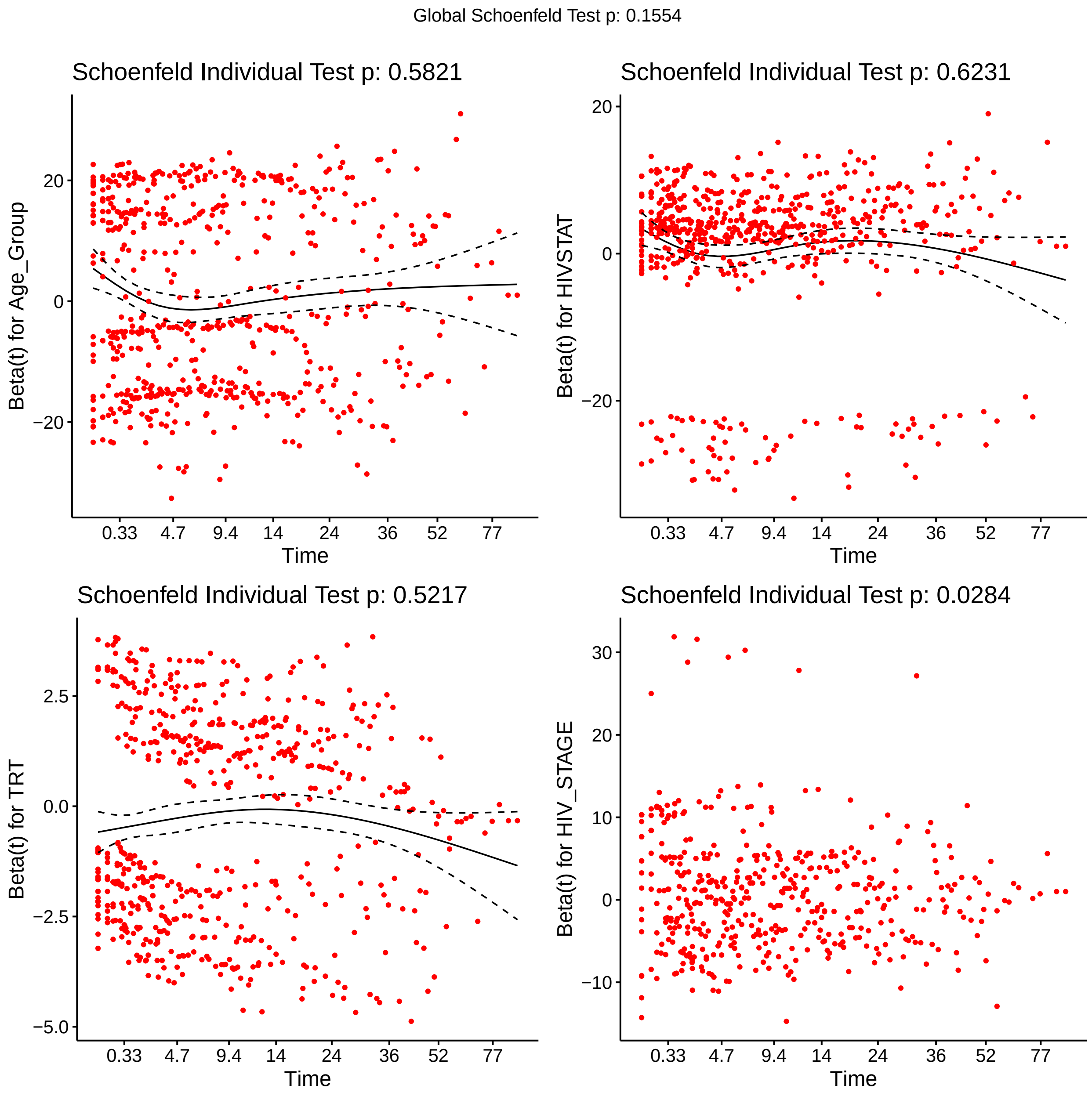

- NO PH VIOLATION

SENSITIVITY ANALYSIS

HIV Count by Age

```
# Cross-tabulation
table(cervical$Age_Group, cervical$HIVSTAT)
```

```
##
```

```
##         Negative Positive Unknown
##   <39         28      295     248
##   >50        181      318     541
##   40-49       44      401     293
```

```
# Copy Data set for sens analysis
cervical_sens <- cervical
```

Best/Worst case (All HIV cases treated as uniform)

```
# Re-run model removing HIV status
ns_alluni_model <- coxph(Surv(time2death, Censor) ~ Age_Group + TRT + strata(STAGE, BAS), data = cervica

summary(ns_alluni_model)
```

```
## Call:
## coxph(formula = Surv(time2death, Censor) ~ Age_Group + TRT +
##     strata(STAGE, BAS), data = cervical)
##
##   n= 2349, number of events= 603
##
##                    coef exp(coef) se(coef)      z Pr(>|z|)
## Age_Group>50    0.09416   1.09873  0.10681  0.882    0.378
## Age_Group40-49 -0.05389   0.94753  0.11550 -0.467    0.641
## TRTYes         -0.37504   0.68726  0.08905 -4.211 2.54e-05 ***
## ---
## Signif. codes:  0 '***' 0.001 '**' 0.01 '*' 0.05 '.' 0.1 ' ' 1
##
##                exp(coef) exp(-coef) lower .95 upper .95
## Age_Group>50      1.0987     0.9101    0.8912    1.3546
## Age_Group40-49    0.9475     1.0554    0.7556    1.1883
## TRTYes            0.6873     1.4551    0.5772    0.8183
##
## Concordance= 0.564  (se = 0.016 )
## Likelihood ratio test= 20.08  on 3 df,   p=2e-04
## Wald test            = 20.07  on 3 df,   p=2e-04
## Score (logrank) test = 20.22  on 3 df,   p=2e-04
```

```
summary(ns_alluni_model)$concordance
```

```
##          C      se(C)
## 0.56377961 0.01615493
```

Note: You omit HIVSTAT from the model since it no longer varies. Since there is no variation, it will not be informative to the regression. The goal is to see how the estimates change when HIV status is uniformly assumed to be positive.

```
#Compare to main model
# Summary of the main model
summary(nsstagestratcox)
```

```
## Call:
## coxph(formula = Surv(time2death, Censor) ~ Age_Group + HIVSTAT +
##     TRT + strata(STAGE, BAS), data = cervical)
##
##   n= 2349, number of events= 603
##
##                     coef exp(coef) se(coef)      z Pr(>|z|)
## Age_Group>50     0.12086   1.12846  0.11035  1.095 0.273433
## Age_Group40-49  -0.04359   0.95734  0.11562 -0.377 0.706155
## HIVSTATPositive  0.18200   1.19961  0.13191  1.380 0.167657
## HIVSTATUnknown   0.23721   1.26771  0.13699  1.732 0.083339 .
## TRTYes          -0.35303   0.70255  0.09082 -3.887 0.000101 ***
## ---
## Signif. codes:  0 '***' 0.001 '**' 0.01 '*' 0.05 '.' 0.1 ' ' 1
##
##                 exp(coef) exp(-coef) lower .95 upper .95
## Age_Group>50       1.1285     0.8862    0.9090    1.4009
## Age_Group40-49     0.9573     1.0446    0.7632    1.2008
## HIVSTATPositive    1.1996     0.8336    0.9263    1.5535
## HIVSTATUnknown     1.2677     0.7888    0.9692    1.6581
## TRTYes             0.7026     1.4234    0.5880    0.8394
##
## Concordance= 0.57  (se = 0.016 )
## Likelihood ratio test= 23.21  on 5 df,   p=3e-04
## Wald test            = 23.25  on 5 df,   p=3e-04
## Score (logrank) test = 23.43  on 5 df,   p=3e-04
```

- The hazard ratio for treatment (TRTYes) remains significant and fairly stable across models.
- A slight decrease in HR from 0.7022 to 0.6864 shows marginal strengthening of the treatment effect when assuming everyone is HIV+ or HIV–.
- The concordance index also slightly decreases from 0.570 to 0.563, suggesting a minor drop in model discrimination without HIVSTAT.

These results indicate that the effect of treatment on survival is robust to assumptions about HIV status even when it's entirely excluded from the model. This strengthens confidence in the treatment finding, while still acknowledging potential residual confounding due to missing HIV data.

Using Prevalence rate to create positive and negative cases from the unknown group

```r
# Calculate the prevalence rate

# Check frequency of each category
table(cervical$HIVSTAT)
```

```
##
## Negative Positive  Unknown
##      253     1014     1082
```

```r
# Filter to only known HIV status
known_hiv <- cervical %>% filter(HIVSTAT %in% c("Positive", "Negative"))

# Calculate prevalence
hiv_prev <- mean(known_hiv$HIVSTAT == "Positive")
hiv_prev
```

```
## [1] 0.8003157
```

```r
set.seed(2025)  # For reproducibility

# Copy the dataset
cervical$HIVSTAT_prev <- cervical$HIVSTAT  # Start with original values

# Get indices of unknowns
unknown_idx <- which(cervical$HIVSTAT == "Unknown")

# Sample based on calculated prevalence
assigned <- sample(c("Positive", "Negative"),
                   size = length(unknown_idx),
                   replace = TRUE,
                   prob = c(hiv_prev, 1 - hiv_prev))

# Assign the imputed values
cervical$HIVSTAT_prev[unknown_idx] <- assigned

# Convert to factor (optional but recommended)
cervical$HIVSTAT_prev <- factor(cervical$HIVSTAT_prev, levels = c("Negative", "Positive"))

# Check new distribution
table(cervical$HIVSTAT_prev)
```

```
##
## Negative Positive
##      482     1867
```

```r
#Run prevalence sensitivity model
sens_prev_model <- coxph(Surv(time2death, Censor) ~ Age_Group + HIVSTAT_prev + TRT + strata(STAGE, BAS)
summary(sens_prev_model)
```

```
## Call:
## coxph(formula = Surv(time2death, Censor) ~ Age_Group + HIVSTAT_prev +
##     TRT + strata(STAGE, BAS), data = cervical)
##
##   n= 2349, number of events= 603
##
##                          coef exp(coef) se(coef)      z Pr(>|z|)
## Age_Group>50          0.10721   1.11316  0.10935  0.980    0.327
## Age_Group40-49       -0.05133   0.94996  0.11558 -0.444    0.657
## HIVSTAT_prevPositive  0.05639   1.05801  0.10304  0.547    0.584
## TRTYes               -0.37421   0.68784  0.08902 -4.204 2.63e-05 ***
## ---
```

```
## Signif. codes:  0 '***' 0.001 '**' 0.01 '*' 0.05 '.' 0.1 ' ' 1
##
##                      exp(coef) exp(-coef) lower .95 upper .95
## Age_Group>50            1.1132     0.8983    0.8984    1.3792
## Age_Group40-49          0.9500     1.0527    0.7574    1.1915
## HIVSTAT_prevPositive    1.0580     0.9452    0.8645    1.2948
## TRTYes                  0.6878     1.4538    0.5777    0.8189
##
## Concordance= 0.565  (se = 0.016 )
## Likelihood ratio test= 20.38  on 4 df,   p=4e-04
## Wald test            = 20.4  on 4 df,   p=4e-04
## Score (logrank) test = 20.55  on 4 df,   p=4e-04
```

- The hazard ratio for treatment (TRTYes) remains significant and fairly stable when comparing the main model to the prevalence-imputed model.
- A slight decrease in HR from 0.7022 to 0.6871 indicates a marginal strengthening of the treatment effect after imputing HIV status based on the observed prevalence rate (80% positive).
- The concordance index also shows minimal change from 0.570 to 0.565, suggesting only a slight reduction in the model's discriminatory power.

These results indicate that the effect of treatment on survival is robust to assumptions regarding missing HIV status, even when unknown cases are probabilistically assigned using population-level prevalence. This reinforces the validity of the treatment effect while accounting for potential bias from incomplete HIV data.

OVERALL TAKEAWAY

To assess the robustness of our findings to missing HIV status data, we conducted a series of sensitivity analyses. First, we ran models assuming all individuals with unknown HIV status were either HIV-positive or HIV-negative; in both cases, HIV status was excluded from the model due to lack of variability. We then performed a single imputation using the observed HIV prevalence among known cases (80%) to randomly assign HIV status to individuals with missing values. Across all models, the effect of treatment (TRTYes) on survival remained statistically significant and consistent in magnitude (HR range: 0.686–0.702), with concordance indices ranging from 0.563 to 0.570. These results suggest that our primary findings are robust to different assumptions regarding HIV status, including complete-case, uniform, and prevalence-based imputation strategies.

Unknown as Negative

```r
# Create a new variable
cervical_sens$HIVSTAT_allneg <- cervical_sens$HIVSTAT
cervical_sens$HIVSTAT_allneg[cervical_sens$HIVSTAT_allneg == "Unknown"] <- "Negative"

# Confirm the change
table(cervical_sens$HIVSTAT_allneg)
```

```
##
## Negative Positive
##     1335     1014
```

```r
# Fit the Cox model
sens_allneg_model <- coxph(Surv(time2death, Censor) ~ Age_Group + HIVSTAT_allneg + TRT + strata(STAGE, B

# model summary
summary(sens_allneg_model)
```

```
## Call:
## coxph(formula = Surv(time2death, Censor) ~ Age_Group + HIVSTAT_allneg +
##     TRT + strata(STAGE, BAS), data = cervical_sens)
##
##   n= 2349, number of events= 603
##
##                             coef exp(coef) se(coef)      z Pr(>|z|)
## Age_Group>50             0.09951   1.10463  0.10997  0.905    0.366
## Age_Group40-49          -0.05355   0.94786  0.11551 -0.464    0.643
## HIVSTAT_allnegPositive   0.01816   1.01832  0.08877  0.205    0.838
## TRTYes                  -0.37754   0.68554  0.08988 -4.200 2.67e-05 ***
## ---
## Signif. codes:  0 '***' 0.001 '**' 0.01 '*' 0.05 '.' 0.1 ' ' 1
##
##                         exp(coef) exp(-coef) lower .95 upper .95
## Age_Group>50               1.1046     0.9053    0.8905    1.3703
## Age_Group40-49             0.9479     1.0550    0.7558    1.1887
## HIVSTAT_allnegPositive     1.0183     0.9820    0.8557    1.2118
## TRTYes                     0.6855     1.4587    0.5748    0.8176
##
## Concordance= 0.565  (se = 0.016 )
## Likelihood ratio test= 20.12  on 4 df,   p=5e-04
## Wald test            = 20.12  on 4 df,   p=5e-04
## Score (logrank) test = 20.26  on 4 df,   p=4e-04
```

Unknown as Positive

```r
# Copy the dataset
cervical_sens$HIVSTAT_allpos <- cervical_sens$HIVSTAT

# Recode all "Unknown" values as "Positive"
cervical_sens$HIVSTAT_allpos[cervical_sens$HIVSTAT_allpos == "Unknown"] <- "Positive"

# Confirm counts
table(cervical_sens$HIVSTAT_allpos)
```

```
##
## Negative Positive
##      253     2096
```

```r
sens_allpos_model <- coxph(Surv(time2death, Censor) ~ Age_Group + HIVSTAT_allpos + TRT + strata(STAGE, B

summary(sens_allpos_model)
```

```
## Call:
## coxph(formula = Surv(time2death, Censor) ~ Age_Group + HIVSTAT_allpos +
##     TRT + strata(STAGE, BAS), data = cervical_sens)
##
##   n= 2349, number of events= 603
##
##                             coef exp(coef) se(coef)      z Pr(>|z|)
## Age_Group>50             0.13118   1.14017  0.10886  1.205    0.228
## Age_Group40-49          -0.04424   0.95672  0.11561 -0.383    0.702
## HIVSTAT_allposPositive   0.20536   1.22797  0.12519  1.640    0.101
## TRTYes                  -0.36299   0.69559  0.08911 -4.074 4.63e-05 ***
## ---
## Signif. codes:  0 '***' 0.001 '**' 0.01 '*' 0.05 '.' 0.1 ' ' 1
##
##                         exp(coef) exp(-coef) lower .95 upper .95
## Age_Group>50               1.1402     0.8771    0.9211    1.4113
## Age_Group40-49             0.9567     1.0452    0.7627    1.2000
## HIVSTAT_allposPositive     1.2280     0.8144    0.9608    1.5694
## TRTYes                     0.6956     1.4376    0.5841    0.8283
##
## Concordance= 0.57  (se = 0.016 )
## Likelihood ratio test= 22.89  on 4 df,   p=1e-04
## Wald test            = 22.86  on 4 df,   p=1e-04
## Score (logrank) test = 23.03  on 4 df,   p=1e-04
```

SENSITIVITY MODELS COMPARISON TABLE

```r
# Helper function to extract model info
extract_model_info <- function(model, model_name) {
  s <- summary(model)
  coefs <- s$coefficients
  conf <- s$conf.int
  concordance <- round(s$concordance[1], 4)  # Extract and round the concordance index

  # Create a data frame for each variable in the model
  df <- data.frame(
    Model = model_name,
    Variable = rownames(coefs),
    HR = round(coefs[, "exp(coef)"], 4),
    CI_Lower = round(conf[, "lower .95"], 4),
    CI_Upper = round(conf[, "upper .95"], 4),
    P_Value = format(round(coefs[, "Pr(>|z|)"], 4), nsmall = 4, scientific = FALSE),
    Concordance = concordance,
    stringsAsFactors = FALSE
  )

  return(df)
}


# Create tables for each model
main_model_info <- extract_model_info(nsstagestratcox, "Main Model")
```

```r
prev_model_info <- extract_model_info(sens_prev_model, "Prevalence-Imputed")
neg_model_info  <- extract_model_info(sens_allneg_model, "All Unknown = Negative")
pos_model_info  <- extract_model_info(sens_allpos_model, "All Unknown = Positive")
uni_model_info  <- extract_model_info(ns_alluni_model, "Uniform HIV (Omit HIVSTAT)")

# Combine all into one table
sens_model_comparison <- rbind(
  main_model_info,
  prev_model_info,
  neg_model_info,
  pos_model_info,
  uni_model_info
)

# View or save the table
print(sens_model_comparison)
```

```
##                                             Model              Variable     HR
## Age_Group>50                           Main Model          Age_Group>50 1.1285
## Age_Group40-49                         Main Model        Age_Group40-49 0.9573
## HIVSTATPositive                        Main Model       HIVSTATPositive 1.1996
## HIVSTATUnknown                         Main Model        HIVSTATUnknown 1.2677
## TRTYes                                 Main Model                TRTYes 0.7026
## Age_Group>501                  Prevalence-Imputed          Age_Group>50 1.1132
## Age_Group40-491                Prevalence-Imputed        Age_Group40-49 0.9500
## HIVSTAT_prevPositive           Prevalence-Imputed   HIVSTAT_prevPositive 1.0580
## TRTYes1                        Prevalence-Imputed                TRTYes 0.6878
## Age_Group>502              All Unknown = Negative          Age_Group>50 1.1046
## Age_Group40-492            All Unknown = Negative        Age_Group40-49 0.9479
## HIVSTAT_allnegPositive     All Unknown = Negative HIVSTAT_allnegPositive 1.0183
## TRTYes2                    All Unknown = Negative                TRTYes 0.6855
## Age_Group>503              All Unknown = Positive          Age_Group>50 1.1402
## Age_Group40-493            All Unknown = Positive        Age_Group40-49 0.9567
## HIVSTAT_allposPositive     All Unknown = Positive HIVSTAT_allposPositive 1.2280
## TRTYes3                    All Unknown = Positive                TRTYes 0.6956
## Age_Group>504          Uniform HIV (Omit HIVSTAT)          Age_Group>50 1.0987
## Age_Group40-494        Uniform HIV (Omit HIVSTAT)        Age_Group40-49 0.9475
## TRTYes4                Uniform HIV (Omit HIVSTAT)                TRTYes 0.6873
##                        CI_Lower CI_Upper P_Value Concordance
## Age_Group>50             0.9090   1.4009  0.2734      0.5703
## Age_Group40-49           0.7632   1.2008  0.7062      0.5703
## HIVSTATPositive          0.9263   1.5535  0.1677      0.5703
## HIVSTATUnknown           0.9692   1.6581  0.0833      0.5703
## TRTYes                   0.5880   0.8394  0.0001      0.5703
## Age_Group>501            0.8984   1.3792  0.3269      0.5652
## Age_Group40-491          0.7574   1.1915  0.6569      0.5652
## HIVSTAT_prevPositive     0.8645   1.2948  0.5842      0.5652
## TRTYes1                  0.5777   0.8189  0.0000      0.5652
## Age_Group>502            0.8905   1.3703  0.3655      0.5647
## Age_Group40-492          0.7558   1.1887  0.6430      0.5647
## HIVSTAT_allnegPositive   0.8557   1.2118  0.8379      0.5647
## TRTYes2                  0.5748   0.8176  0.0000      0.5647
## Age_Group>503            0.9211   1.4113  0.2282      0.5695
```

```
## Age_Group40-493           0.7627   1.2000  0.7019      0.5695
## HIVSTAT_allposPositive    0.9608   1.5694  0.1009      0.5695
## TRTYes3                   0.5841   0.8283  0.0000      0.5695
## Age_Group>504             0.8912   1.3546  0.3780      0.5638
## Age_Group40-494           0.7556   1.1883  0.6408      0.5638
## TRTYes4                   0.5772   0.8183  0.0000      0.5638
```

```r
# write.csv(full_model_table, "sensitivity_model_comparison.csv", row.names = FALSE)
```
